# Compressed deepfake detection via GA-LASSO selection of deep features and machine learning models

**DOI:** 10.1038/s41598-025-34733-6

**Published:** 2026-03-23

**Authors:** Abdel Motalib Lagsoun, Oussama khouili, Aissam Bekkari, Noura Boudra, Mustapha Oujaoura, Abdelilah Jraifi, Saïd Ech-chadi, Mustapha Hedabou

**Affiliations:** 1 Mathematics, Informatics & Communication Systems Laboratory (MISCOM), National School of Applied Sciences of Safi, Cadi Ayyad University, Marrakech, 40000 Morocco; 2 LTI Laboratory, National School of Applied Sciences, Chouaib Doukkali University, El Jadida, 24000 Morocco; 3 Laboratory of Complex Systems Modeling, National School of Applied Sciences of Marrakech, Cadi Ayyad University, Marrakech, 40000 Morocco; 4 College of Computing, Mohammed VI Polytechnic University (UM6P), Ben Guerir, 43150 Morocco

**Keywords:** Deepfake detection, Genetic algorithm, Lasso regularization, Feature selection, Machine, Computational biology and bioinformatics, Engineering, Mathematics and computing

## Abstract

In an era of advanced synthetic media, deepfake detection is challenged by high-dimensional feature spaces, compression artifacts, and poor generalization. This paper proposes a hybrid feature-selection framework combining genetic algorithms (GA) with LASSO regularization to reduce redundancy in ResNet50 embeddings from 2048 to 120–170 features (>90% reduction). Experiments on FaceForensics++ (FF++) and Celeb-DF v2 under C0, C23, and C40 compression show improved accuracy, efficiency, and robustness. In single-seed evaluations, the method achieves AUC = 99.48 and 97.11% accuracy (KNN, Deepfakes C23) and remains competitive under cross-dataset and cross-manipulation testing. On Celeb-DF v2 with harsh C40 compression, SVM achieves AUC = 78.74, outperforming many end-to-end models. Multi-seed analysis shows consistent top-tier performance across datasets (e.g., FF++ C40: 85.2% accuracy; Celeb-DF C0: 88.7%). GA+LASSO maintains accuracy comparable to GA while substantially reducing computational cost, particularly under heavy compression (Celeb-DF C40). Overall, the proposed framework enhances accuracy, generalization, and stability while reducing feature dimensionality and computational cost, offering a lightweight and robust deepfake detection solution suited to real-world media conditions.

## Introduction

In recent years, high-performance computing breakthroughs have enabled the creation of highly sophisticated deepfake creation techniques^1^. These synthetic media have the ability to produce convincingly realistic fake images, videos, and audio, raising critical issues for public trust, privacy, and security^2^. Abuse scenarios range from political speech manipulation and celebrity face swaps to undermining digital evidence, with real-world instances showing their potential impact. In particular, in 2019, an AI voice impersonation scammed a UK company out of $243,000^3^, and in 2023, a manipulated image of an explosion near the Pentagon caused brief market volatility before being debunked^4^. In response to these threats, researchers have explored deep learning (DL) approaches such as automatic feature extraction, traditional machine learning with handcrafted features, and frequency domain forensic methods^5,6^. Cutting-edge multimodal and ensemble methods fuse audio–visual cues, temporal discrepancies, and transformer-based architectures for improved robustness^7^. However, most methods do not generalize across manipulation types and media qualities, and many have high annotated data and computational expenses^8^. Although hardware accelerators such as GPUs and FPGAs have enabled real-time inference, the environmental footprint of large DL models remains a growing concern^9^. In this paper, we present a hybrid detector using **ResNet-50** features with **GA+LASSO** selection, evaluated via **XGBoost, logistic regression, KNN, SVM and random forest**. Focusing on compression robustness, our method shows superior accuracy and cross-dataset performance on **FF++** and **Celeb-DF v2** versusthe state-of-the-art methods.

This work offers the following key contributions:

- Hybrid detection framework combining ResNet-50 deep feature extraction with traditional ML classifiers, improving both generalizability and interpretability.
- Two-stage feature selection (GA + LASSO) to drastically reduce dimensionality while preserving discriminative power.
- Robust performance in high compression and cross-dataset settings, outperforming state-of-the-art baselines on FF++ and Celeb-DF v2.

The paper is structured as follows. Section 2 reviews related work on data compression and classification. Section 3 details the proposed methodology, including feature extraction, compression, and classifiers. Section 4 presents experimental results across compression levels. Section 5 discusses the findings, limitations, and future directions. Section 6 highlights the limitations of the specific method. Finally, Section 7 summarizes key findings and suggests future research.

## Related work

### Recent deepfake detection methods

Deepfakes, facilitated by advances in GANs and deep neural networks, are increasing the difficulty of distinguishing between artificial and authentic media, with implications for misinforcement, privacy, and digital forensics^10^. The detection mechanisms vary from traditional machine learning to deep learning models, including CNN-based models such as MesoNet^11^ and frequency-domain models such as FreqNet^12^, which detect subtle forgery patterns. Multimodal approaches have also been shown, leveraging visual–audio discrepancies for enhanced robustness^13^. In addition to that, feature selection has become a vital factor in maximizing detection efficiency and generalization. Moreover, hierarchically optimized handcrafted and deep feature-based hybrid methods have been shown to have competitive performance on benchmark datasets such as Celeb-DF v2, FF++, and DFDC^14^. Other works have aimed at interpretable and efficient architectures, combining wavelet-inspired spatiotemporal descriptors and sparse CNN classifiers and displaying robust cross-dataset performance for various compression settings^15^. The FDINet59^16^, for example, a 59-layer Fake Dense Inception Network, was developed to detect such deepfakes using datasets prepared with MTCNN. It achieved up to 94.95% accuracy on deepfakes generated by autoencoders and GANs. This demonstrates its potential to help prevent the dissemination of deceptive content online. This study proposes LSTM-AE-DRDE^17^, a framework combining attention-enhanced LSTM with contrastive learning and residual difference encoding to capture temporal and contextual audio cues. Using diverse features such as MFCC, prosodic, wavelet, and glottal parameters, the model achieved high accuracies across five benchmark datasets (95–97%) and an overall ROC-AUC of 98%, outperforming eleven state-of-the-art methods. Feature-wise ablation studies further confirmed the robustness and reliability of the approach.

### Machine learning-based deepfake detection

Machine learning approaches have also been shown to function under cross-dataset and low-quality scenarios^18^. Methods range from CNN pipelines optimized for computational cost^19^ to supervised models incorporating facial landmarks for inconsistency detection, such as abnormal blinking or misaligned features^20^. Studies comparing CNN models to old classifiers such as KNN and SVM have demonstrated better accuracy and robustness with the former^21^. Notably, hybrid systems where CNN feature extraction is combined with traditional classifiers such as random forests or SVMs have further enhanced detection performance on public datasets such as FF++ and Celeb-DF^22^. This work^23^ introduces a deepfake detection pipeline combining ResNet50 feature extraction with UMAP dimensionality reduction for efficient classification. Tested on FaceForensics++ (C23, C40), it achieved state-of-the-art results, with Random Forest reaching 97.3% accuracy/99.1 AUC and KNN 96.5% accuracy/99.1 AUC.

### Heuristic and metaheuristic feature selection techniques in deepfake detection

Nature-inspired metaheuristics such as (the GA) and particle swarm optimization (PSO) have been applied for feature selection and hyperparameter tuning. This method enhances detection precision at a reduced computational expense^24^. Hybrid optimization methods based on more than one metaheuristic, such as ACO–PSO^25^ or Coot Bird Optimization (CBO)^26^, have shown better performance in terms of accuracy and resilience. Moreover, PSO-based feature selection has also been shown to significantly reduce feature dimensionality while improving classification efficiency on benchmarks such as OpenForensics^27^.

## Methodology

**Fig. 1:**
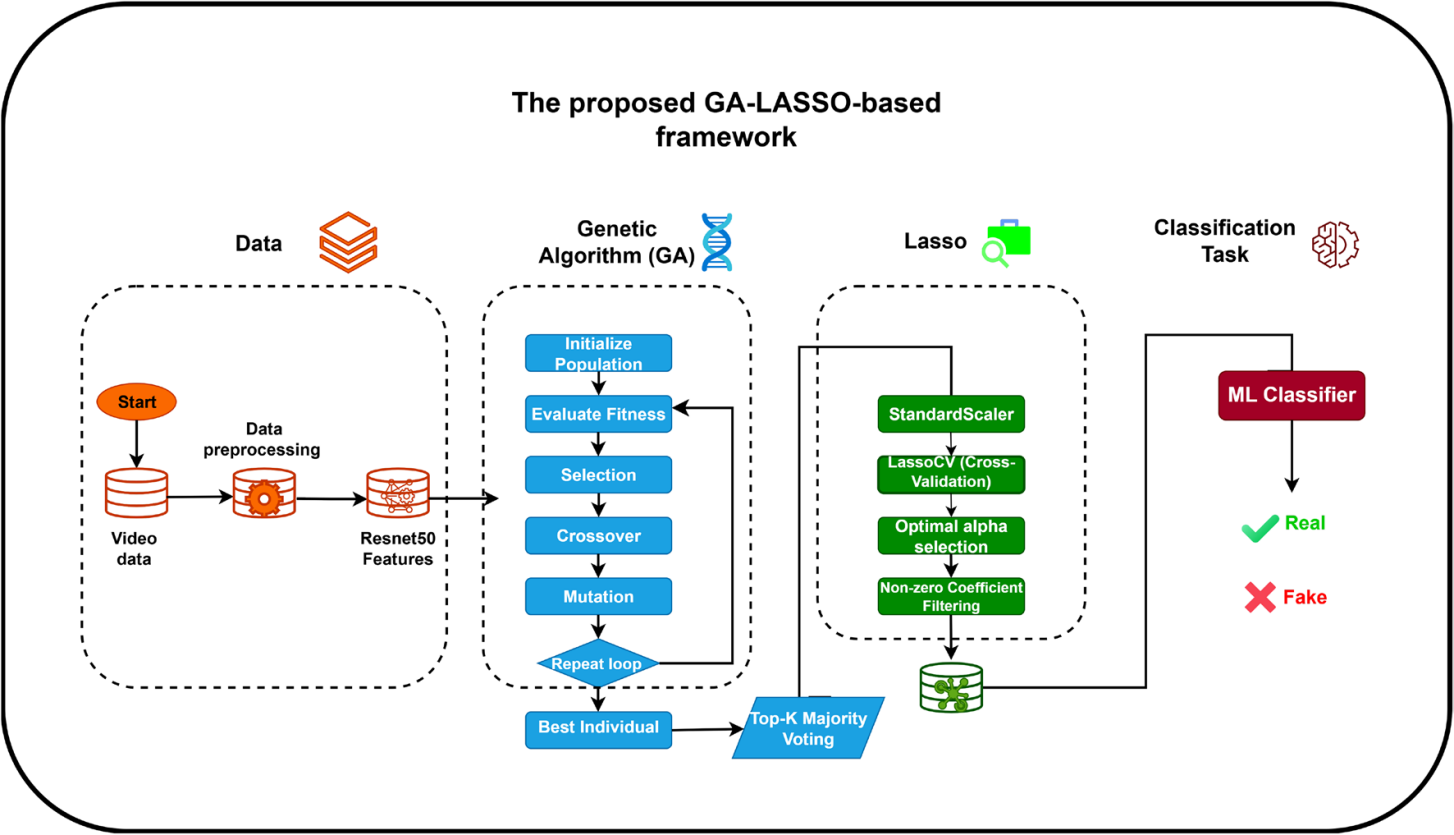
Flowchart of the proposed hybrid deepfake detection pipeline combining ResNet50 feature extraction, the (GA) and LASSO-based feature selection, and final classification.

We propose a hybrid pipeline for deepfake detection that combines heuristic and statistical feature selection methods, as illustrated in Figure 1 and detailed in Algorithm 1. Initially, features are extracted using ResNet50, processed in batches, and aggregated into a unified feature matrix. A (GA) is applied to identify a compact subset of discriminative characteristics by optimizing the classification accuracy through a random forest model as the fitness function. Subsequently, the LASSO regression further refines the selected features by removing those with near-zero coefficients, which results in a reduction of dimensionality while preserving informative content. The resulting feature subset is then used to train multiple classifiers, whose performance is evaluated on a held-out test set using accuracy, AUC and confusion matrix metrics. This approach exploits the complementary strengths of evolutionary search and sparse modeling to effectively handle high-dimensional feature spaces and enhance deepfake detection performance.

### Feature extraction

ResNet50 is a prominent 50-layer deep architecture in the ResNet family, consisting of 49 convolutional layers and a single fully connected layer. The residual network architecture was introduced to counter the vanishing gradient problem by means of skip connections that allow gradient flow in very deep networks. Hence, ResNet50 can learn more complex and detailed image features than shallower models can.

We opted fo ResNet50 since it has been widely established to be effective in various computer vision tasks, particularly in recognizing patterns in various types of datasets. Specifically, we utilized ResNet50 to extract feature vectors from images after stripping its last classification layer. The images are preprocessed by cropping facial areas to ResNet50 for feature extraction. We intentionally skip further preprocessing operations to avoid possible distortions, since our goal is to capture faint features related to deepfake artifacts.

**Algorithm 1:**
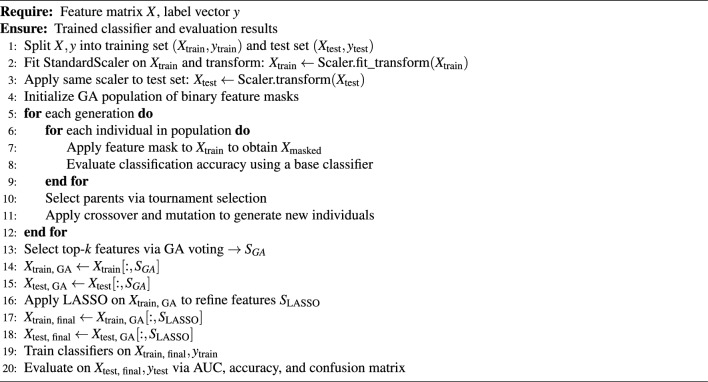
Hybrid GA+LASSO Feature Selection and Classification.

### Genetic algorithm for feature selection

High-dimensional feature spaces are a common characteristic of deepfake detection systems, especially those incorporating deep neural representations or handcrafted features across multiple modalities. While rich in information, such representations often contain redundant or irrelevant features that may impair classifier performance and increase computational cost. To mitigate these challenges, we propose a wrapper-based feature selection strategy leveraging a (GA). The (GA) facilitates efficient exploration of the combinatorial feature space, aiming to identify a compact, discriminative subset of features that maximizes classification accuracy.

Each candidate solution in the GA is represented as a binary chromosome:

1 $$\begin{aligned} \textbf{z} = [z_1, z_2, \ldots , z_d] \in \{0,1\}^d, \end{aligned}$$

where $d$ denotes the total number of features, and where $z_j = 1$ implies that the $j$-th feature is selected, while $z_j = 0$ indicates its exclusion. This binary encoding is well-suited for subset selection tasks and allows seamless integration with genetic operators such as crossover and mutation.

The initial population $\mathcal {P}^{(0)}$ of size $N_p$ is generated randomly. Each bit in a chromosome is initialized according to a Bernoulli distribution:

2 $$\begin{aligned} z_j \sim \text {Bernoulli}(0.5), \quad \forall j \in \{1, \ldots , d\}, \end{aligned}$$

ensuring unbiased sampling across the search space in the initial generation.

To evaluate the quality of a solution $\textbf{z}$, we employ a random forest classifier trained on the feature subset defined by $\textbf{z}$. Specifically, for a given training dataset $\textbf{X} \in \mathbb {R}^{n \times d}$, we extract the reduced feature matrix:

3 $$\begin{aligned} \textbf{X}_{\textbf{z}} = \{ \textbf{x}_i^{\textbf{z}} \in \mathbb {R}^{\Vert \textbf{z}\Vert _0} \mid i = 1, \ldots , n \}, \end{aligned}$$

where $\Vert \textbf{z}\Vert _0 = \sum _{j=1}^d \mathbb {I}[z_j = 1]$ is the number of selected features. The performance of the classifier is quantified in terms of accuracy on a held-out validation set. To cast this as a minimization problem compatible with GA optimization, we define the fitness function as:

4 $$\begin{aligned} \text {Fitness}(\textbf{z}) = 1 - \text {Accuracy}(\textbf{z}). \end{aligned}$$

Parent selection is performed via binary tournament selection, which is a method that balances selective pressure and population diversity. In each tournament, two chromosomes are selected randomly from the current population, and the one with the lower fitness (i.e., higher accuracy) is chosen to participate in reproduction.

Offspring generation is performed via single-point crossover, applied with probability $p_c$. Given two parents $\textbf{z}^{(1)}$ and $\textbf{z}^{(2)}$, a crossover point $k \in \{1, \ldots , d-1\}$ is sampled uniformly, and the offspring are created as:

5 $$\begin{aligned} & \textbf{z}^{\text {child}_1} = [z_1^{(1)}, \ldots , z_k^{(1)}, z_{k+1}^{(2)}, \ldots , z_d^{(2)}], \end{aligned}$$

6 $$\begin{aligned} & \textbf{z}^{\text {child}_2} = [z_1^{(2)}, \ldots , z_k^{(2)}, z_{k+1}^{(1)}, \ldots , z_d^{(1)}]. \end{aligned}$$

To preserve genetic diversity and prevent stagnation, mutation is applied to each gene in the offspring chromosomes independently with probability $p_m$. The mutation operation simply flips the bit value:

7 $$\begin{aligned} z_j := 1 - z_j, \quad \forall j \text { such that } \text {Uniform}(0,1) < p_m. \end{aligned}$$

The evolutionary cycle comprising fitness evaluation, parent selection, crossover, and mutation is repeated for $G$ generations or until a convergence criterion is met (e.g., no improvement over successive generations). Upon completion, the algorithm selects the top-$k$ individuals (i.e., those with the lowest fitness) for consensus-based feature aggregation.

To further increase robustness and reduce the effect of randomness, we adopt a majority voting strategy across the top-$k$ individuals. For each feature index $j \in \{1, \ldots , d\}$, we compute the support count:

8 $$\begin{aligned} s_j = \sum _{l=1}^{k} z_j^{(l)}, \end{aligned}$$

and include feature $j$ in the final subset if:

9 $$\begin{aligned} s_j \ge \tau \cdot k, \end{aligned}$$

where $\tau \in [0,1]$ is a user-defined threshold controlling the strictness of the majority vote.

The final selected feature set is denoted as:

10 $$\begin{aligned} \mathcal {F}_{\text {GA}} = \left\{ j \mid s_j \ge \tau \cdot k \right\} . \end{aligned}$$

This GA-based framework offers several advantages: it scales to high-dimensional spaces, adapts dynamically to the search landscape, and promotes selection of robust, generalizable feature subsets. By directly integrating classifier performance into the fitness function, the method aligns feature selection with the end task of deepfake classification, yielding improved accuracy and reduced model complexity.

### LASSO-based feature refinement

To further refine the feature subset obtained from the GA stage and remove redundant or weakly informative features, we apply the least absolute shrinkage and selection operato (LASSO). LASSO performs both variable selection and regularization within a linear model framework, promoting sparsity in the solution.

Let $\textbf{X}_{\text {GA}} \in \mathbb {R}^{n \times d'}$ denote the reduced feature matrix from the GA stage, where $d' \ll d$. We first normalize the features:

11 $$\begin{aligned} \tilde{\textbf{X}}_{\text {GA}} = \frac{\textbf{X}_{\text {GA}} - \boldsymbol{\mu }}{\boldsymbol{\sigma }} \end{aligned}$$

where $\boldsymbol{\mu }$ and $\boldsymbol{\sigma }$ represent the column-wise means and standard deviations, respectively.

The LASSO optimization objective is as follows:

12 $$\begin{aligned} \hat{\boldsymbol{\beta }} = \arg \min _{\boldsymbol{\beta }} \left\{ \frac{1}{2n} \left\| \textbf{y} - \tilde{\textbf{X}}_{\text {GA}} \boldsymbol{\beta } \right\| _2^2 + \alpha \Vert \boldsymbol{\beta } \Vert _1 \right\} \end{aligned}$$

where $\alpha> 0$ is the regularization parameter controlling sparsity. A larger $\alpha$ enforces stronger shrinkage, potentially eliminating more features.

To determine $\alpha$, we perform 5-fold cross-validation over a logarithmic grid:

13 $$\begin{aligned} \alpha \in \{ \alpha _1, \alpha _2, \ldots , \alpha _T \} \end{aligned}$$

and select the value that minimizes the mean squared error on the validation folds.

The final selected features are as follows:

14 $$\begin{aligned} \mathcal {F}_{\text {LASSO}} = \{ j \mid \hat{\beta }_j \ne 0 \} \end{aligned}$$

and the corresponding refined feature matrix is as follows :

15 $$\begin{aligned} \textbf{X}_{\text {final}} = \tilde{\textbf{X}}_{\text {GA}}[:, \mathcal {F}_{\text {LASSO}}] \end{aligned}$$

By combining the GA and LASSO, our framework achieves both global feature search and local sparsity refinement, leading to a compact, interpretable, and high-performing representation for deepfake classification.

### Classifier

After feature extraction and feature selection have been completed, the resulting data are structured into a unified feature matrix suitable for classification. Given that deepfake detection is the central objective of this study, this stage focuses on evaluating various classifiers to determine which model achieves the most accurate and robust performance.

We evaluated five classification algorithms: random forest, XGBoost, logistic regression, KNN, and SVM. Each was selected on the basis of its effectiveness in handling high-dimensional data and its established performance in classification tasks. Random forest and XGBoost, as ensemble methods, offer strong generalization capabilities and resistance to overfitting. Logistic regression provides a linear baseline model that is interpretable and efficient. KNN, using a neighborhood-based approach, was applied for its simplicity and non-parametric nature. SVM was used with a radial basis function kernel to capture non-linear relationships in the feature space.

## Experiments

### Dataset

Experiments were conducted on two benchmark deepfake detection datasets: FF++^28^ and Celeb-DF v2^29^. FF++ contains over 1,000 manipulated videos using multiple methods (Deepfakes^30^, Face2Face^31^, FaceSwap^32^, NeuralTextures^33^, and FaceShifter^34^), with C23 and C40 compression levels, split 70/30 for training/testing. Celeb-DF v2 includes 590 real and 5,639 high-quality fake videos, which are further compressed to C40 for robustness evaluation.

### Implementation details

The experiments were conducted on a standard laptop (Intel Core i7-12700H, 32GB RAM, NVIDIA RTX 3070 Ti) using Python 3.10.4 with TensorFlow/Keras 2.10.0 and scikit-learn. The GA completed in 6.15 hours (50 generations, selecting 1,050 features), whereas LASSO refinement ($\alpha$ = 0.01) took 1.61 seconds to reduce the number of features to 132. Five classifiers were evaluated with standard configurations: XGBoost (use_label_encoder=False, eval_metric=’logloss’), logistic regression (max_iter=1000), KNN (n_neighbors=5), and SVM (RBF kernel, probability=True). This setup demonstrated efficient feature selection and robust classification across different learning paradigms without requiring high-performance systems.

**Table 1:** Feature Extraction and Selection Results on FF++ C23 and C40 Compressed Videos.

**Dataset**	**Res Features**	**GA Best Fitness Score**	**GA Selected**	**LASSO Selected**
**C23 Compression**				
Deepfake	(29692, 2048)	0.9146	997	143
Face2Face	(29665, 2048)	0.8325	1078	171
FaceShifter	(29682, 2048)	0.8850	1083	163
FaceSwap	(26679, 2048)	0.8671	1061	159
NeuralTextures	(26674, 2048)	0.7742	1034	124
**C40 Compression**				
Deepfake	(10000, 2048)	0.7780	1014	144
Face2Face	(10000, 2048)	0.6803	1021	142
FaceShifter	(9975, 2048)	0.7778	989	132
FaceSwap	(9985, 2048)	0.7186	1010	136
NeuralTextures	(9990, 2048)	0.6266	1054	119

**Table 2:** Feature Extraction and Selection Results on Celeb-DF v2 C0 and C40 Compressed Videos.

**Dataset**	**Res Features (Train)**	**Res Features (Test)**	**GA Fitness**	**GA Selected**	**LASSO Selected**
Celeb-DF V2 (C0)	(30000, 2048)	(3000, 2048)	0,8373	1016	135
Celeb-DF V2 (C40)	(30000, 2048)	(3000, 2048)	0,7476	1032	113

**Table 3:** Performance (AUC/Accuracy) of different classifiers across datasets under C23 and C40 compression.

**Dataset**	**Random Forest**	**SVM**	**KNN**	**Logistic Regression**	**XGBoost**
**C23 Compression**					
Deepfakes	0.9706/0.9073	0.9591/0.8945	**0.9948/0.9711**	0.9418/0.8693	0.9803/0.9252
Face2Face	0.9143/0.8307	0.9296/0.8598	0.9156/0.8324	0.9047/0.8263	**0.9390/0.8642**
FaceSwap	0.9463/0.8656	0.9493/0.8827	**0.9888/0.9558**	0.9292/0.8548	0.9678/0.9048
NeuralTextures	0.8781/0.7918	0.8788/0.8039	0.8627/0.7852	0.8575/0.7840	**0.9028/0.8242**
FaceShifter	0.9586/0.8919	0.9568/0.8926	**0.9880/0.9500**	0.9445/0.8760	0.9761/0.9218
**C40 Compression**					
Deepfakes	0.8598/0.7787	0.8821/0.8027	0.8685/0.7980	0.8712/0.7960	**0.8879/0.8043**
Face2Face	0.7532/0.6840	0.7784/0.7033	0.6136/0.5700	0.7826/0.7020	**0.8879/0.8043**
FaceSwap	0.7216/0.7977	0.8108/0.7390	0.7736/0.7163	0.7910/0.7190	**0.8161/0.7433**
NeuralTextures	0.6887/0.6346	0.7168/0.6590	0.5395/0.5269	**0.7229/0.6597**	0.6932/0.6333
FaceShifter	0.8578/0.7725	0.8745/0.7905	0.8007/0.7337	0.8671/0.7848	**0.8836/0.7942**

**Table 4:** Cross-manipulation detection performance (AUC/Accuracy) on FF++ C23 compression. Each cell reports results when training on the specified manipulation and testing on another.

**Train**	**Test**	**XGBoost**	**Logistic Reg**	**KNN**	**SVM**	**RandForest**
Deepfakes	Face2Face	0.7681/0.5967	0.6508/0.5721	0.5779/0.5244	0.6773/0.5702	0.9665/0.6110
	FaceShifter	0.7960/0.6251	0.6438/0.5695	0.8187/0.6787	0.6843/0.5858	0.9713/0.6456
	FaceSwap	0.5525/0.5929	0.3475/0.5179	0.8528/0.7480	0.4085/0.5444	0.8994/0.6086
	NeuralTextures	0.8268/0.6621	0.7074/0.6416	0.5633/0.5683	0.7445/0.6481	0.9773/0.6675
Face2Face	Deepfakes	0.8581/0.7277	0.7357/0.6631	0.7746/0.6890	0.7562/0.6762	0.9953/0.7488
	FaceShifter	0.6610/0.5937	0.5097/0.5047	0.6981/0.6321	0.5343/0.5351	0.9755/0.6264
	FaceSwap	0.6923/0.6571	0.5855/0.5840	0.7037/0.6632	0.5934/0.6052	0.9690/0.6636
	NeuralTextures	0.7999/0.6821	0.6687/0.6196	0.7463/0.6908	0.6993/0.6387	0.9831/0.7003
FaceShifter	Deepfakes	0.8530/0.6778	0.6382/0.5814	0.9035/0.7877	0.6734/0.5963	0.9909/0.7049
	Face2Face	0.6598/0.5586	0.5131/0.5049	0.6030/0.5375	0.5380/0.5135	0.9481/0.5795
	FaceSwap	0.6160/0.6082	0.4453/0.5314	0.8021/0.7018	0.4566/0.5344	0.9404/0.6240
	NeuralTextures	0.7774/0.6414	0.6301/0.6010	0.5963/0.5832	0.6531/0.6095	0.9711/0.6662
FaceSwap	Deepfakes	0.6723/0.5669	0.3992/0.4761	0.9194/0.7877	0.4722/0.5038	0.9666/0.5539
	Face2Face	0.7382/0.5875	0.6185/0.5533	0.5893/0.5294	0.6380/0.5580	0.9602/0.5731
	FaceShifter	0.6474/0.5539	0.4721/0.4872	0.7999/0.6458	0.4795/0.4986	0.9558/0.5459
	NeuralTextures	0.5228/0.5748	0.4302/0.5210	0.5193/0.5591	0.4282/0.5336	0.9011/0.5729
NeuralTextures	Deepfakes	0.8705/0.7317	0.7022/0.6362	0.7648/0.6899	0.7376/0.6567	0.9966/0.7283
	Face2Face	0.6540/0.7899	0.6376/0.5756	0.7605/0.6789	0.6639/0.5904	0.9887/0.6590
	FaceShifter	0.8056/0.6812	0.6314/0.5789	0.7288/0.6622	0.6700/0.6035	0.9876/0.6900
	FaceSwap	0.5056/0.5906	0.3841/0.4997	0.5511/0.5815	0.3800/0.5144	0.9162/0.5944

**Table 5:** Cross-manipulation detection performance (AUC/Accuracy) on FF++ C40 compression. Each cell reports results when training on the specified manipulation and testing on another.

**Train**	**Test**	**XGBoost**	**Logistic Reg**	**KNN**	**SVM**	**RandForest**
Deepfakes	Face2Face	0.6248/0.5616	0.6177/0.5613	0.5828/0.5419	0.6200/0.5623	0.6161/0.5676
	FaceShifter	0.7111/0.6349	0.7049/0.6350	0.7340/0.6497	0.7173/0.6348	0.6992/0.6362
	FaceSwap	0.6105/0.5626	0.5422/0.5213	0.7727/0.6880	0.5651/0.5376	0.5990/0.5618
	NeuralTextures	0.5895/0.5504	0.5980/0.5549	0.5623/0.5276	0.5963/0.5494	0.5854/0.5466
Face2Face	Deepfakes	0.6601/0.6058	0.6328/0.5974	0.6129/0.5840	0.6465/0.6076	0.6569/0.6085
	FaceShifter	0.6114/0.5771	0.5832/0.5548	0.6036/0.5804	0.5983/0.5603	0.6166/0.5747
	FaceSwap	0.5917/0.5538	0.5373/0.5240	0.5800/0.5619	0.5543/0.5370	0.6021/0.5541
	NeuralTextures	0.6977/0.6250	0.6183/0.5737	0.6385/0.6053	0.6292/0.5812	0.7265/0.6278
FaceShifter	Deepfakes	0.7296/0.6589	0.7178/0.6509	0.7553/0.6925	0.7295/0.6588	0.7109/0.6478
	Face2Face	0.5859/0.5489	0.5627/0.5300	0.5809/0.5468	0.5692/0.5375	0.5962/0.5523
	FaceSwap	0.6177/0.5759	0.5840/0.5485	0.6916/0.6354	0.5979/0.5525	0.6174/0.5684
	NeuralTextures	0.5904/0.5464	0.5839/0.5408	0.5729/0.5439	0.5904/0.5448	0.5953/0.5586
FaceSwap	Deepfakes	0.6574/0.6090	0.5861/0.5634	0.7994/0.7306	0.6281/0.5902	0.6593/0.6120
	Face2Face	0.5867/0.5507	0.5455/0.5261	0.5616/0.5421	0.5624/0.5341	0.6061/0.5515
	FaceShifter	0.6356/0.5902	0.6230/0.5840	0.7016/0.6423	0.6424/0.5967	0.6481/0.6018
	NeuralTextures	0.5518/0.5229	0.5157/0.5082	0.5429/0.5237	0.5205/0.5114	0.5708/0.5273
NeuralTextures	Deepfakes	0.6081/0.5731	0.6042/0.5704	0.5761/0.5557	0.6197/0.5814	0.6387/0.5947
	Face2Face	0.7317/0.6576	0.6435/0.6008	0.6552/0.6192	0.6582/0.6120	0.7591/0.6663
	FaceShifter	0.6201/0.5848	0.6404/0.5994	0.6037/0.5833	0.6591/0.6147	0.6286/0.5891
	FaceSwap	0.5482/0.5262	0.5136/0.5104	0.5460/0.5391	0.5100/0.5064	0.5767/0.5371

#### Feature extraction and selection efficiency

**Fig. 2:**
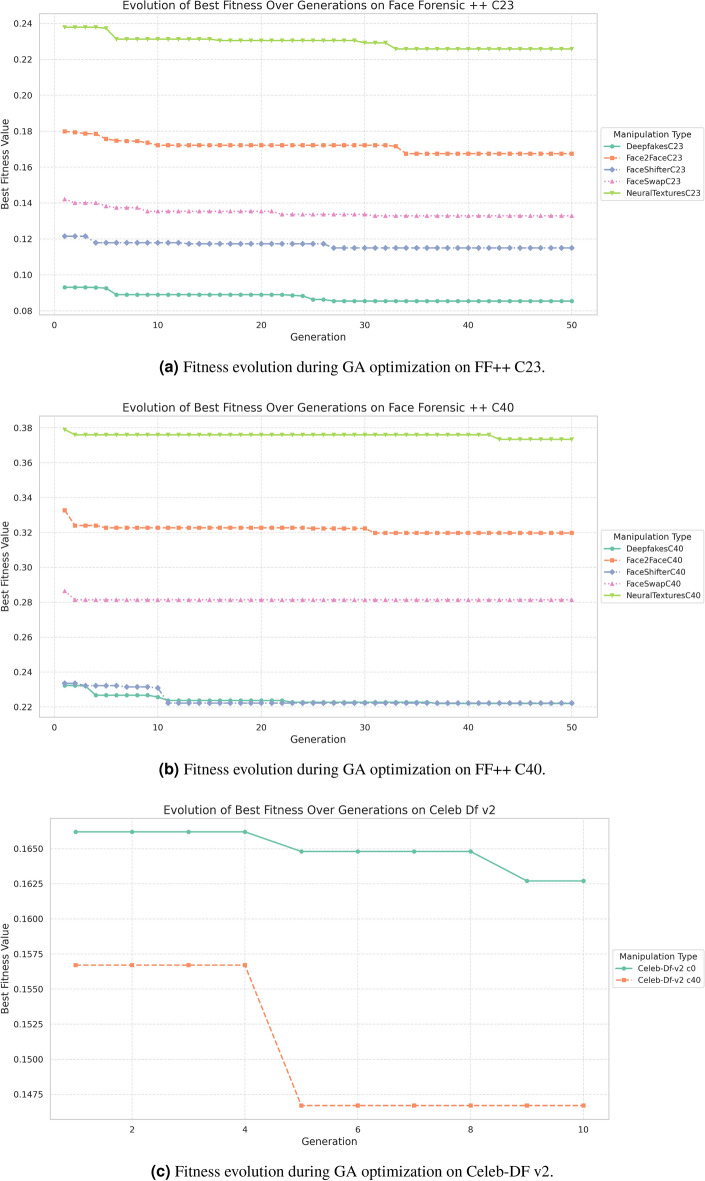
Comparison of Genetic Algorithm (GA) fitness score evolution across FF++ (C23, C40) and Celeb-DF v2 (C0, C40) datasets. The fitness improves over generations as the algorithm searches for optimal feature subsets.

Tables 1 and 2 present the feature extraction/selection results. ResNet-50 extracts (2048-D) deep semantic features, compressed via GA + LASSO. GA selected 997–1083 features (fitness: 0.9146 for Deepfakes C23, 0.8373 for Celeb-DF v2 C0), further pruned to 119–171 features by LASSO. As shown in Figure 2, the GA’s fast convergence (10–50 genes), with stable fitness for high-quality (C23) videos but lower scores under heavy compression (C40, e.g., NeuralTextures). Celeb-DF v2 optimization uses 10 genes because of the rapid convergence of FF++.

For Celeb-DF v2, fitness dropped from 0.1662 (C0) to 0.1567 (C40), highlighting compression’s impact of comparison on feature separability. GA+LASSO achieves robust dimensionality reduction, lowering computational costs and improving interpretability without sacrificing accuracy.

#### Classifier performance and robustness to compression

**Fig. 3:**
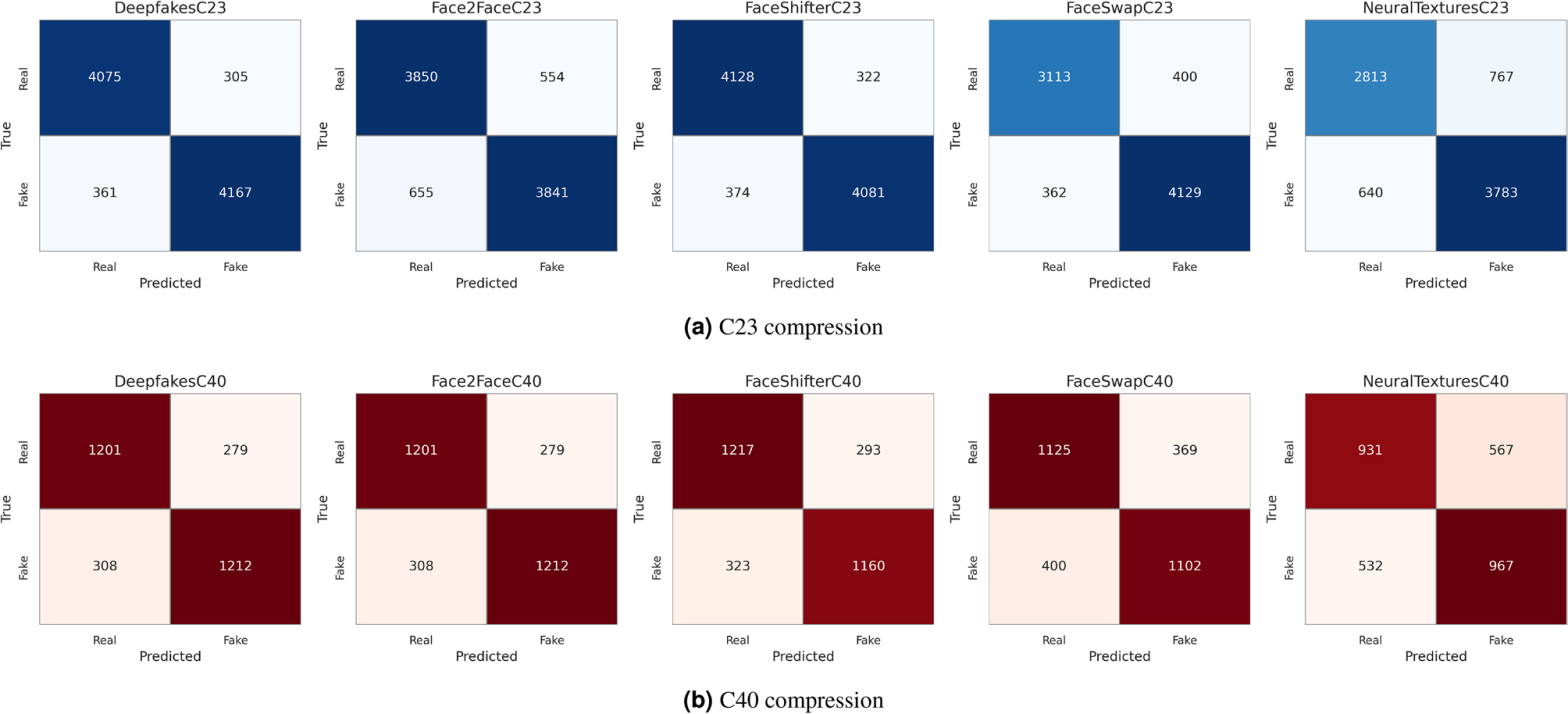
Confusion matrices for XGBoost classifier under C23 and C40 compression settings.

**Fig. 4:**
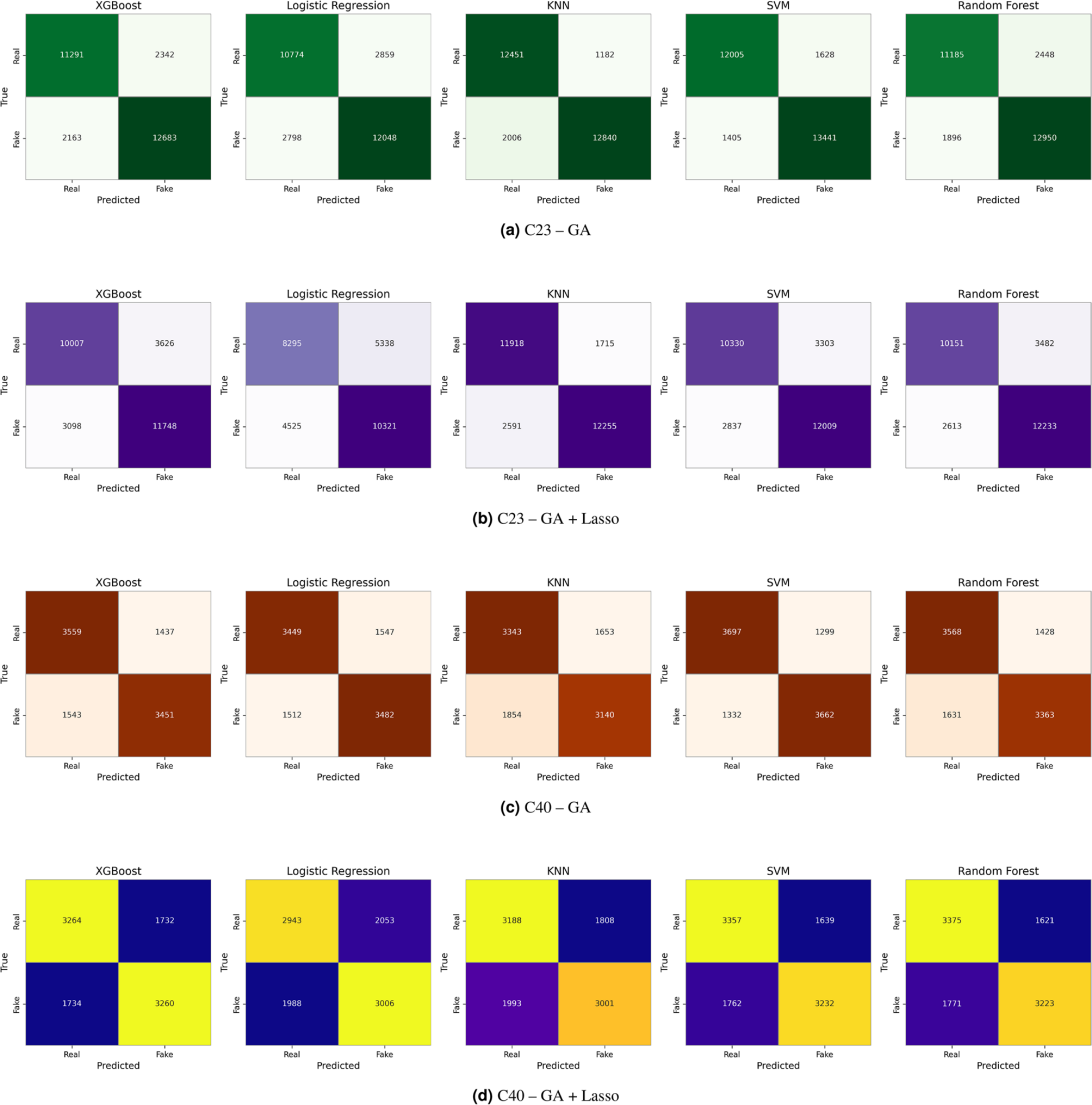
Confusion matrices for five classifiers under different feature selection configurations: GA and GA+Lasso, across C23 and C40 compression levels in the FF++ dataset. Each figure shows results for XGBoost, Logistic Regression, KNN, SVM, and Random Forest.

Table 3 shows that the KNN method excels on the C23 data (DF: 99.48/97.11 AUC/Acc), whereas XGBoost and random forest dominate FS/F2F classification. Under C40 compression, the three-based methods maintain robustness (XGBoost: 0.8879/0.8043 for DF), outperforming SVM and Logistic Regression. XGBoost confusion matrices (Figs. 3-4) demonstrate strong C23 performance (DF: 4075R/4167F correct), but increased C40 errors (NT: 567R/532F wrong), confirming the compression resilience of the tree model for bandwidth-limited applications.

#### Cross-manipulation generalization capability

The cross-manipulation evaluation (Tables 4 and 5) demonstrates the method’s generalizability across forgery types. For C23 compression, the random forest model showed superior transferability, achieving 0.9713/0.6456 AUC/accuracy (DF$\rightarrow$FSh) and 97.73/66.75 (DF$\rightarrow$NT), with F2F-trained models similarly maintaining AUCs above 96% for FS and NT. Under C40 compression, performance declined but remained competitive, with random forest and XGBoost providing the most robust results (e.g., NT$\rightarrow$F2F: 75.91/66.63). These results confirm the pipeline’s effectiveness across manipulation types and compression levels, supporting real-world deployment in various data conditions.

**Table 6:** Performance of various methods across datasets and compression levels. Accuracy (Acc) and AUC scores are reported.

**Method**	**FF++ C23**	**FF++ C40**	**Celeb-DF v2 C0**	**Celeb-DF v2 C40**
MPNN^35^	−/94.21	−/76.31	−/−	−/−
F3-Net^36^	99.3/98.95	95.8/93.02	−/−	−/−
HiFE (Xception)^37^	−/92.83	−/71.84	−/96.64	−/−
TALL-Swin^38^	99.87/98.65	94.57/92.82	−/−	−/−
ViViT^39^	−/−	−/−	−/87.17	−/−
MfDfD (RGB)^15^	99.09/99.81	99.16/99.64	72.23/91.60	69.48/78.20
**Ours (GA) + XGBoost**	92.18/84.18	77.54/70.17	93.63/87.03	57.53/50.90
**Ours (GA) + Logistic Reg**	88.11/80.14	76.83/69.38	90.53/82.47	75.37/68.27
**Ours (GA) + KNN**	96.00/88.81	70.57/64.89	87.54/81.00	51.78/51.00
**Ours (GA) + SVM**	95.53/89.35	81.85/73.66	92.09/85.00	78.74/71.47
**Ours (GA) + RandForest**	92.74/84.75	77.00/76.98	90.09/81.93	56.77/48.33
**Ours (GA+LASSO) +XGBoost**	84.75/76.39	70.89/65.31	92.26/85.33	60.54/55.97
**Ours (GA+LASSO) +Logistic Reg**	70.83/65.37	63.20/59.55	86.86/78.97	76.43/69.47
**Ours (GA+LASSO) +KNN**	93.05/84.88	66.25/61.95	88.53/82.90	55.96/55.30
**Ours (GA+LASSO) +SVM**	86.46/78.44	71.75/65.96	90.15/82.10	76.84/69.57
**Ours (GA+LASSO) + RandForest**	87.21/78.60	72.29/66.05	90.76/82.63	58.42/48.33

#### Evaluation of GA and GA+Lasso feature selection

**Fig. 5:**
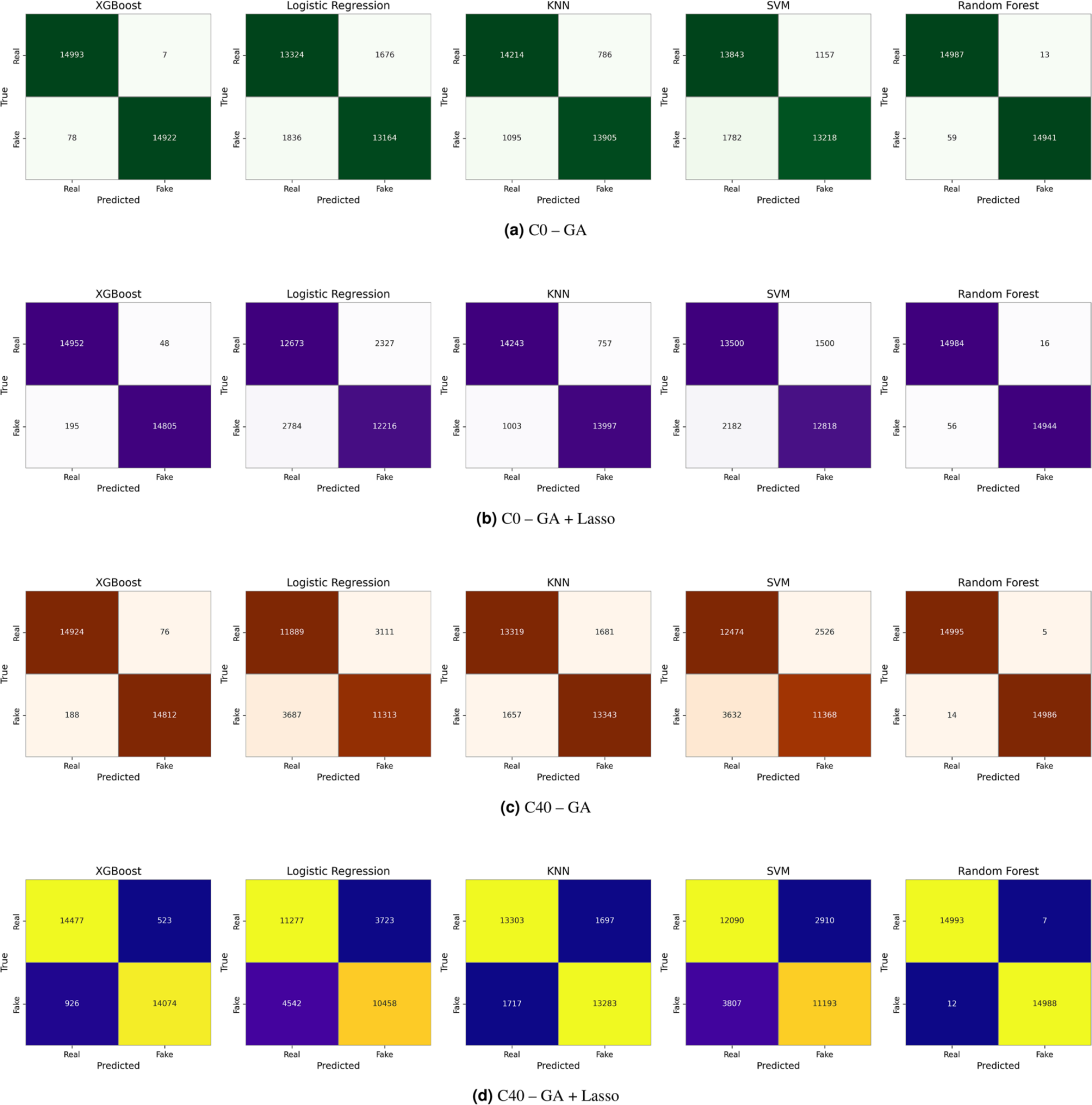
Confusion matrices for five classifiers under different feature selection configurations: GA and GA+Lasso, across C0 and C40 compression levels in the Celeb-DF v2 dataset. Each figure shows results for XGBoost, Logistic Regression, KNN, SVM, and Random Forest.

Table 6 compares five classifiers using the GA and GA+LASSO feature selection methods across datasets. GA consistently outperformed GA+LASSO, with KNN and SVM achieving the top C23 results (96.00/88.81 and 95.53/89.35 AUC/accuracy for FF++, respectively). This advantage persisted in Celeb-DF v2 C0 (GA+XGBoost: 93.63/87.03 vs GA+LASSO: 92.26/85.33). Under C40 compression, the GA was superior (FF++: SVM 81.85/73.66 vs GA+LASSO logistic regression 63.20/59.55; Celeb-DF: SVM 78.74/71.47 vs 76.84/69.57). While GA+LASSO enforces sparsity, it removes subtle discriminative patterns, whereas GA preserves higher-dimensional structure, yielding cleaner decision boundaries (Figs. 4-5 for details).

#### Comparison with SOTA deepfake detection methods

Our method was benchmarked against SOTA approaches (MPNN^35^, F3-Net^36^, etc.) as shown in Table 6. While deep models such as TALL-Swin achieved near-perfect FF++ C23 performance (99.87 AUC), our GA+KNN delivered competitive results (0.9599 AUC/0.8881 accuracy). The gap widened under C40 compression (MfDfD: 99.16 AUC vs our GA+SVM: 0.8185/0.7366). On Celeb-DF v2, GA+XGBoost (0.9363/0.8703) approached ViViT (87.17%) and MfDfD (91.60%), with GA+SVM even surpassing MfDfD in terms of C40 accuracy (71.47% vs 78.20%). Notably, GA+LASSO+Logistic Regression showed promise in high-compression scenarios (69.47% accuracy). This demonstrates that our method’s balanced performance, offering interpretability with cross-dataset robustness, particularly under compression.

### Deep analysis research

To validate the proposed method, we perform a rigorous multi-seed evaluation that incorporates a diverse set of feature-extraction strategies and classification models. Ten classifiers are assessed with fixed random seeds and standardized training (the scaler fitted only on the training split) to ensure reproducibility: RandomForest (n_estimators=200, max_depth=10, random_state=42), ExtraTrees (n_estimators=200, max_depth=10, random_state=42), GradientBoosting (n_estimators=150, learning_rate=0.05, max_depth=3), AdaBoost (n_estimators=100), XGBoost (use_label_encoder=False, eval_metric=’logloss’, n_estimators=200, learning_rate=0.05, random_state=42), SVM-RBF (C=1.0, gamma=’scale’, probability=True), KNN (n_neighbors=5), LogisticRegression (solver=’liblinear’, C=1.0, max_iter=2000), MLP (hidden_layer_sizes=(100,), max_iter=1000, random_state=42), and DecisionTree (max_depth=10, random_state=42). Feature selection is performed using eight modular strategies: a Genetic Algorithm (GA) with population_size=20, generations=20, mutation_rate=0.1, crossover_rate=0.8, $\tau$=0.5 and majority voting over the top-*k* individuals; LASSO (LassoCV with alphas=np.logspace(−3,1,50), cv=5); SelectKBest (mutual_info_classif, k=50); RFE (LogisticRegression estimator, n_features_to_select=50); ExtraTrees importance (n_estimators=100, selecting the top 50 features); and hybrid variants that refine the output of a primary selector using LASSO (e.g., ga+lasso). A no-selection baseline utilizing all 2048 ResNet50 features is included. All feature-selection and tuning steps are applied strictly on the training partition and repeated across multiple random seeds to compute confidence intervals and assess the stability of the proposed approach.

#### Theoretical time complexity

Table 7 shows the theoretical time complexity of the feature selection methods. RFE is the most computationally demanding method, requiring up to 2,048 classifier retrainings, followed by GA with 400 evaluations (20 generations $\times$ 20 population). LASSO refinement adds $\sim$2,500 iterations but operates on reduced features, keeping the added cost moderate. In contrast, KBest achieves high efficiency with $O(N \times D \times \log N)$ complexity, while the baseline (no FS) remains fastest at $O(N \times D)$. Overall, the complexity ranking (*RFE* > *GA* > *ExtraTrees* > *KBest* > *None*) highlights a clear trade-off between computational cost and feature selection sophistication, across 270 complete pipeline runs.

**Table 7:** Method Complexity Rankings (Worst to Best).

**Rank**	**Method**	**Computational Complexity/Notes**
1	**RFE Methods (Most Expensive)**	*rfe:* $O(D \times C_{clf}) \approx 2{,}048$ classifier trainings
		*rfe+lasso:* $O(D \times C_{clf} + K \times I \times N \times D^2)$
2	**GA Methods (Very Expensive)**	*ga:* $O(G \times P \times C_{clf}) = 400$ classifier trainings
		*ga+lasso:* $O(G \times P \times C_{clf} + K \times I \times N \times D^2)$
3	**ExtraTrees Methods (Expensive)**	*extratrees:* $O(T \times N \times D \times \log N)$ (100 trees)
		*extratrees+lasso:* Add $K \times I \times N \times k^2$ for LASSO
4	**KBest Methods (Fast)**	*kbest:* $O(N \times D \times \log N) \approx 20M$ operations
		*kbest+lasso:* $O(N \times D \times \log N + K \times I \times N \times k^2)$
5	**None (Fastest)**	*none:* $O(N \times D)$ (scaling only)

Where: S = 3, seeds (random initializations); S = 9, methods (feature selection strategies); G = 20, GA generations; P = 20, GA population size; N ≈ 10000, samples (dataset size); D = 2048, features (ResNet50 output); K = 5, cross-validation folds; I ≈ 500, iterations (LASSO solver).

#### Runtime and feature reduction assessment of selection algorithms

Table 8 shows clear differences in the computational cost of feature-selection methods. RFE and RFE+LASSO are by far the most expensive, requiring several hours per run, whereas GA-based methods offer a more balanced compromise with moderate runtimes and substantial feature reduction. In contrast, filter-based approaches such as KBest and ExtraTrees operate within seconds while consistently selecting 45–50 features.

**Table 8:** Execution Time and Number of Selected Features Across All Datasets.

Method	FF++ C40		FF++ C23		Celeb-DF C0		Celeb-DF C40	
	Time (s)	Feat.	Time (s)	Feat.	Time (s)	Feat.	Time (s)	Feat.
rfe+lasso	36924.1	50	31845.2	50	53019.6	50	50572.0	50
rfe	36566.9	50	31626.1	50	53242.6	50	50213.6	50
ga	4461.2	1025	11471.4	1024	24946.1	1049	25492.4	1049
ga+lasso	4460.6	516	11554.2	721	24989.7	720	25574.1	759
kbest+lasso	50.3	42	101.6	47	248.5	48	249.7	45
kbest	50.2	50	100.5	50	246.5	50	247.2	50
extratrees+lasso	5.7	44	15.7	47	31.1	48	31.9	45
extratrees	5.7	50	14.0	50	29.2	50	29.6	50

**Fig. 6:**
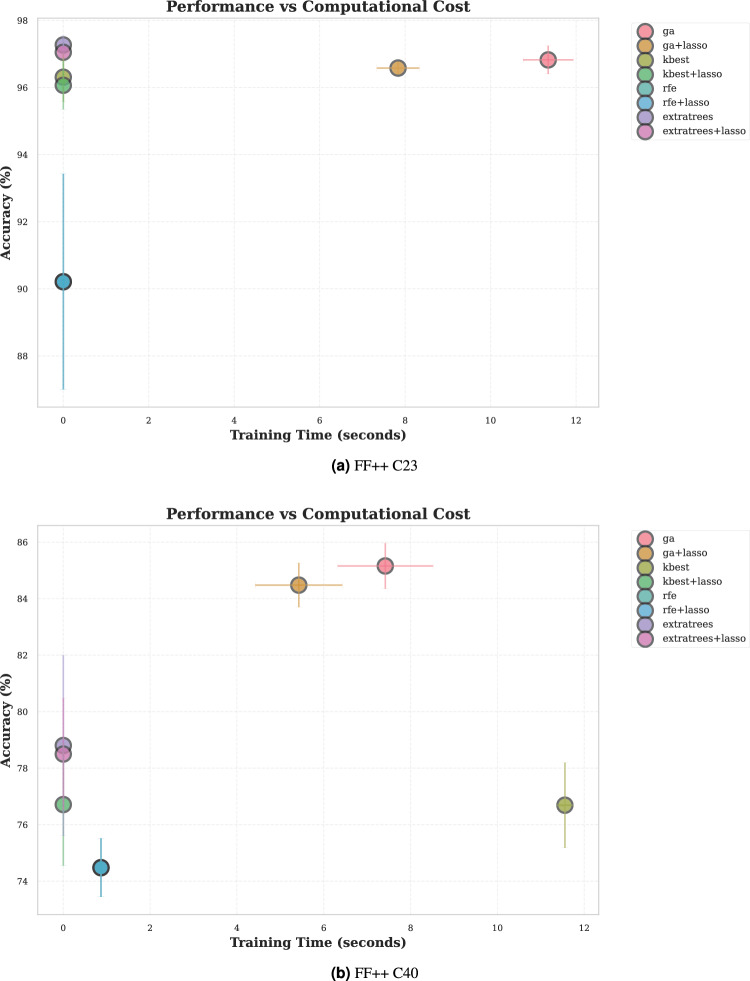
Training Time vs. Accuracy for FF++ dataset under different compression levels.

**Fig. 7:**
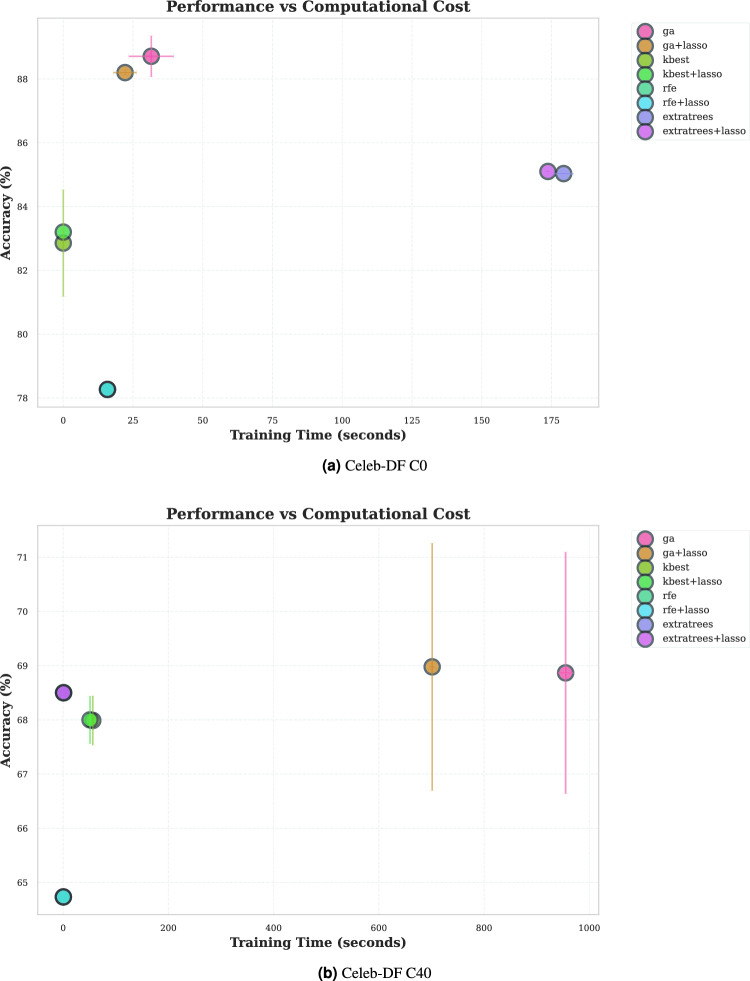
Training Time vs. Accuracy for Celeb-DF dataset under different compression levels.

Figure 6 and 7 further highlights the differences in classifier training time across datasets. For FF++ C23, GA+LASSO and GA require roughly 8 s and 12 s, respectively, while all other methods execute near 0 s. A similar trend appears on FF++ C40, where GA+LASSO takes about 6 s and the remaining methods remain almost instantaneous. On Celeb-DF C0, GA and GA+LASSO require around 25 s, whereas ExtraTrees and ExtraTrees+LASSO exhibit the highest cost at approximately 175 s. Celeb-DF C40 shows the most extreme case, with GA alone reaching nearly 950 s. These results confirm that GA-based pipelines incur moderate additional training cost compared with lightweight selectors, but remain significantly faster than the slowest tree-based classifiers.

#### Statistical significance analysis

Tables 9 and 10 compare feature selection methods across FaceForensics++ (C40/C23) and Celeb-DF (C40/C0), revealing dataset- and quality-dependent behavior.

On FaceForensics++, ga+lasso achieves minimal losses (0.41–0.69%) with no significance on C40. On Celeb-DF C40 (challenging, 56% baseline), tree-based methods paradoxically *improve* performance (+4%), suggesting feature selection removes noise-dominated features from highly degraded data. However, this benefit disappears in Celeb-DF C0 (clean, 82% baseline), where RFE methods suffer severe degradation (8.3%, $p<0.01$).

In contrast, **ga+lasso demonstrates remarkable stability**: <0.7% loss across all four conditions, with statistical equivalence to baseline in three (FaceForensics++ C40, Celeb-DF C40/C0). This consistency across datasets with 56–85% baseline accuracy and varying compression validates GA-based selection as uniquely robust for real-world deepfake detection where data quality fluctuates unpredictably.

**Table 9:** Statistical Significance Comparison Across Compression Levels.

**Method**	**C40 (High Compression)**					**C23 (Low Compression)**				
	**Acc**	**AUC**	**F1**	$\Delta$%	**Sig.**	**Acc**	**AUC**	**F1**	$\Delta$%	**Sig.**
ga+lasso	$\downarrow 0.139$	$\downarrow 0.134$	$\downarrow 0.154$	−0.41%	0/3	$\downarrow <0.001$***	$\downarrow <0.001$***	$\downarrow <0.001$***	−0.69%	3/3
ga	$\downarrow <0.001$***	$\downarrow <0.001$***	$\downarrow <0.001$***	−0.79%	3/3	$\downarrow <0.001$***	$\downarrow <0.001$***	$\downarrow <0.001$***	−0.79%	3/3
extratrees	$\downarrow 0.010$**	$\downarrow 0.011$*	$\downarrow 0.007$**	−2.65%	3/3	$\downarrow 0.001$**	$\downarrow 0.009$**	$\downarrow <0.001$***	−2.80%	3/3
extratrees+lasso	$\downarrow 0.008$**	$\downarrow 0.008$**	$\downarrow 0.007$**	−2.80%	3/3	$\downarrow <0.001$***	$\downarrow 0.006$**	$\downarrow <0.001$***	−2.90%	3/3
kbest	$\downarrow <0.001$***	$\downarrow <0.001$***	$\downarrow <0.001$***	−8.07%	3/3	$\downarrow <0.001$***	$\downarrow <0.001$***	$\downarrow <0.001$***	−4.49%	3/3
kbest+lasso	$\downarrow <0.001$***	$\downarrow <0.001$***	$\downarrow <0.001$***	−8.22%	3/3	$\downarrow <0.001$***	$\downarrow <0.001$***	$\downarrow <0.001$***	−4.68%	3/3
rfe	$\downarrow <0.001$***	$\downarrow <0.001$***	$\downarrow <0.001$***	−8.23%	3/3	$\downarrow <0.001$***	$\downarrow <0.001$***	$\downarrow <0.001$***	−7.01%	3/3
rfe+lasso	$\downarrow <0.001$***	$\downarrow <0.001$***	$\downarrow <0.001$***	−8.24%	3/3	$\downarrow <0.001$***	$\downarrow <0.001$***	$\downarrow <0.001$***	−7.03%	3/3

* $$p<0.05$$, ** $$p<0.01$$, *** $$p<0.001$$ (paired t-test).

$\downarrow$ indicates performance decrease vs baseline (no feature selection).

$\Delta$% = average percentage change across Accuracy, AUC, and F1.

Sig. = number of metrics with $p<0.05$ out of 3 total metrics.

Baseline performance: C40 = 0.71% accuracy, C23 = 0.85% accuracy.

**Table 10:** Statistical Significance Comparison Across Compression Levels celeb DF dataset.

**Method**	**C40 (High Compression)**					**C0 (without Compression)**				
	**Acc**	**AUC**	**F1**	$\Delta$%	**Sig.**	**Acc**	**AUC**	**F1**	$\Delta$%	**Sig.**
extratrees+lasso	$\downarrow 0.081$	$\downarrow 0.100$	$\downarrow 0.087$	+4.01%	0/3	$\downarrow 0.308$	$\downarrow 0.343$	$\downarrow 0.306$	−1.90%	0/3
extratrees	$\downarrow 0.068$	$\downarrow 0.087$	$\downarrow 0.076$	+3.86%	0/3	$\downarrow 0.315$	$\downarrow 0.329$	$\downarrow 0.325$	−1.93%	0/3
kbest+lasso	$\downarrow 0.145$	$\downarrow 0.218$	$\downarrow 0.400$	+2.86%	0/3	$\downarrow 0.058$	$\downarrow 0.050$	$\downarrow 0.063$	−4.18%	0/3
kbest	$\downarrow 0.144$	$\downarrow 0.234$	$\downarrow 0.385$	+2.56%	0/3	$\downarrow 0.065$	$\downarrow 0.063$	$\downarrow 0.072$	−3.86%	0/3
rfe+lasso	$\downarrow 0.824$	$\downarrow 0.685$	$\downarrow 0.744$	+0.14%	0/3	$\downarrow 0.001$**	$\downarrow 0.001$**	$\downarrow 0.001$**	−8.31%	3/3
rfe	$\downarrow 0.832$	$\downarrow 0.679$	$\downarrow 0.735$	+0.13%	0/3	$\downarrow 0.001$**	$\downarrow 0.001$**	$\downarrow 0.001$**	−8.35%	3/3
ga+lasso	$\downarrow 0.840$	$\downarrow 0.342$	$\downarrow 0.633$	−0.30%	0/3	$\downarrow 0.084$	$\downarrow 0.224$	$\downarrow 0.107$	−0.65%	0/3
ga	$\downarrow 0.726$	$\downarrow 0.155$	$\downarrow 0.350$	−0.38%	0/3	$\downarrow 0.043$*	$\downarrow 0.173$	$\downarrow 0.041$*	−0.75%	2/3

* $$p<0.05$$, ** $$p<0.01$$, *** $$p<0.001$$ (paired t-test).

$\downarrow$ indicates performance decrease vs baseline (no feature selection).

$\Delta$% = average percentage change across Accuracy, AUC, and F1.

Sig. = number of metrics with $p<0.05$ out of 3 total metrics.

Baseline performance: C40 = 0.56% accuracy, C0 = 0.82% accuracy.

**Table 11:** Unified Performance Summary of Feature Selection Methods Across All Datasets (Mean ± 95% CI).

**Dataset**	**Method**	**Acc (%)**	**AUC**	**F1**	**Time (s)**
**FF++ c40**	extratrees	$78.80 \pm 3.20$	$0.861 \pm 0.018$	$0.783 \pm 0.037$	0.0
	extratrees+lasso	$78.50 \pm 1.98$	$0.858 \pm 0.024$	$0.781 \pm 0.023$	0.0
	**ga**	$85.16 \pm 0.80$	$0.926 \pm 0.012$	$0.851 \pm 0.009$	7.4
	ga+lasso	$84.48 \pm 0.78$	$0.917 \pm 0.017$	$0.845 \pm 0.011$	5.4
	kbest	$76.69 \pm 1.51$	$0.841 \pm 0.018$	$0.768 \pm 0.016$	11.6
	kbest+lasso	$76.71 \pm 2.16$	$0.831 \pm 0.017$	$0.763 \pm 0.026$	0.0
	rfe	$74.48 \pm 1.04$	$0.822 \pm 0.020$	$0.742 \pm 0.013$	0.9
	rfe+lasso	$74.48 \pm 1.04$	$0.822 \pm 0.020$	$0.742 \pm 0.013$	0.9
**FF++ c23**	**extratrees**	$97.27 \pm 0.27$	$0.995 \pm 0.002$	$0.972 \pm 0.003$	0.0
	extratrees+lasso	$97.05 \pm 0.17$	$0.995 \pm 0.003$	$0.970 \pm 0.002$	0.0
	ga	$96.82 \pm 0.42$	$0.993 \pm 0.001$	$0.968 \pm 0.004$	11.3
	ga+lasso	$96.58 \pm 0.19$	$0.993 \pm 0.002$	$0.966 \pm 0.002$	7.8
	kbest	$96.31 \pm 0.73$	$0.992 \pm 0.001$	$0.962 \pm 0.008$	0.0
	kbest+lasso	$96.07 \pm 0.72$	$0.991 \pm 0.002$	$0.960 \pm 0.008$	0.0
	rfe	$90.21 \pm 3.21$	$0.961 \pm 0.020$	$0.901 \pm 0.032$	0.0
	rfe+lasso	$90.21 \pm 3.21$	$0.961 \pm 0.020$	$0.901 \pm 0.032$	0.0
**Celeb-DF c0**	extratrees	$85.03 \pm 0.00$	$0.919 \pm 0.000$	$0.847 \pm 0.000$	179.3
	extratrees+lasso	$85.10 \pm 0.00$	$0.917 \pm 0.000$	$0.848 \pm 0.000$	173.7
	**ga**	$88.71 \pm 0.64$	$0.945 \pm 0.010$	$0.889 \pm 0.006$	31.5
	ga+lasso	$88.20 \pm 0.08$	$0.945 \pm 0.003$	$0.884 \pm 0.001$	22.1
	kbest	$82.86 \pm 1.67$	$0.894 \pm 0.002$	$0.824 \pm 0.017$	0.0
	kbest+lasso	$83.20 \pm 0.00$	$0.895 \pm 0.006$	$0.827 \pm 0.001$	0.0
	rfe	$78.27 \pm 0.00$	$0.868 \pm 0.000$	$0.784 \pm 0.000$	15.9
	rfe+lasso	$78.27 \pm 0.00$	$0.868 \pm 0.000$	$0.784 \pm 0.000$	15.8
**Celeb-DF c40**	extratrees	$68.50 \pm 0.00$	$0.751 \pm 0.000$	$0.667 \pm 0.000$	0.8
	extratrees+lasso	$68.50 \pm 0.00$	$0.751 \pm 0.000$	$0.666 \pm 0.000$	0.8
	ga	$68.87 \pm 2.23$	$0.753 \pm 0.010$	$0.670 \pm 0.027$	955.0
	**ga+lasso**	$68.98 \pm 2.28$	$0.755 \pm 0.015$	$0.672 \pm 0.020$	701.3
	kbest	$67.99 \pm 0.46$	$0.744 \pm 0.005$	$0.655 \pm 0.006$	56.3
	kbest+lasso	$68.00 \pm 0.44$	$0.744 \pm 0.006$	$0.655 \pm 0.003$	51.0
	rfe	$64.73 \pm 0.00$	$0.697 \pm 0.000$	$0.631 \pm 0.000$	0.3
	rfe+lasso	$64.73 \pm 0.00$	$0.697 \pm 0.000$	$0.631 \pm 0.000$	0.5

Bold values denote best performance per dataset.

As shown in Table 11 (see Tables in Subsection 4.4 for all results), GA alone achieves the highest performance in most evaluation settings. However, the GA+LASSO variant remains valuable because it produces a substantial reduction in feature dimensionality and computation time while maintaining nearly identical accuracy, AUC, and F1. Notably, under the most challenging condition (Celeb-DF C40), GA+LASSO slightly outperforms GA, indicating that the LASSO refinement improves robustness under strong compression. Thus, although GA provides marginally higher peak scores in several scenarios, the hybrid GA+LASSO stage offers a more balanced trade-off between accuracy, sparsity, and efficiency.

**Fig. 8:**
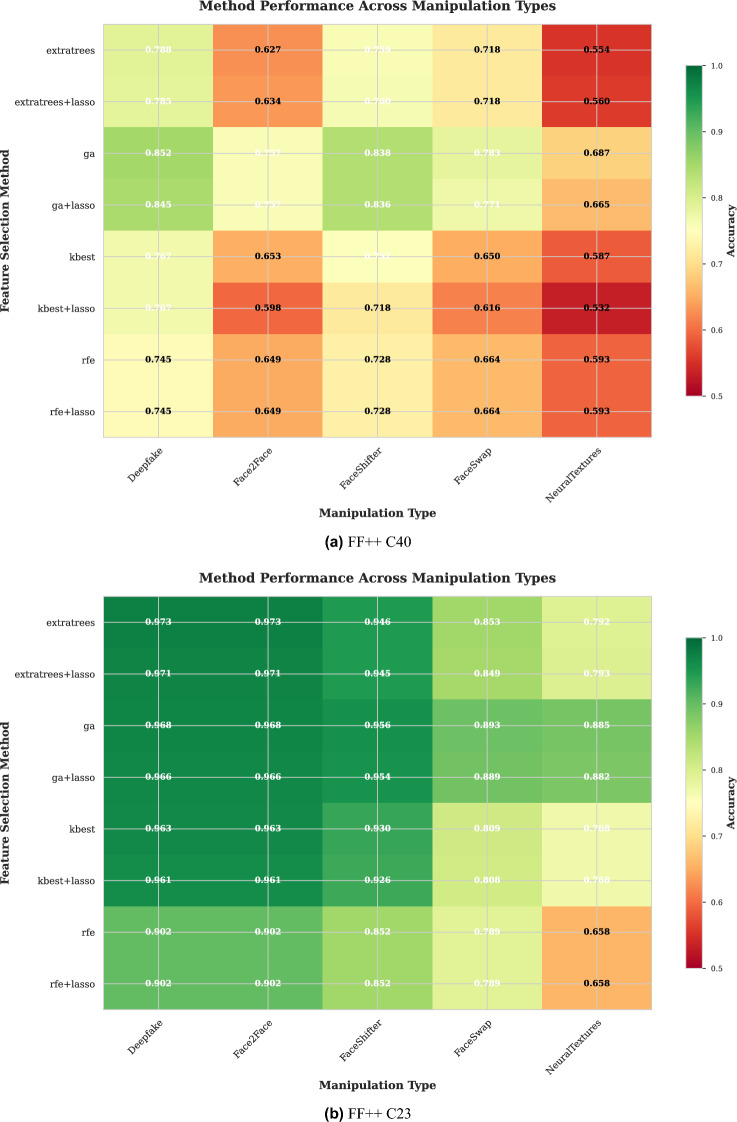
Manipulation-wise performance heatmaps for FF++ dataset.

**Fig. 9:**
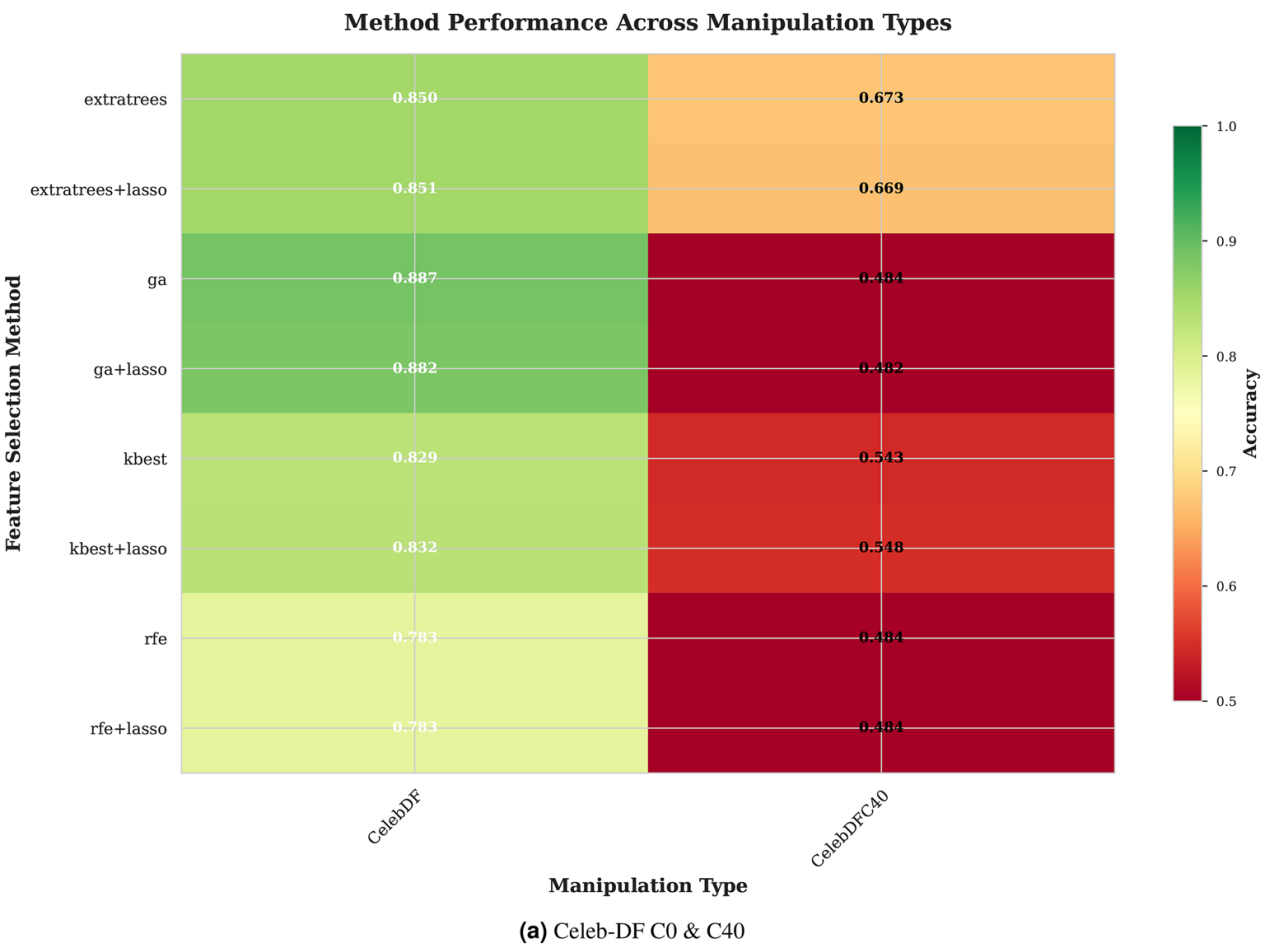
Manipulation-wise performance heatmaps for Celeb-DF dataset.

Figure 8 and 9 further clarifies these trends across individual manipulation types. In FF++ C23, ExtraTrees achieves the highest accuracy on *DeepFake*, *Face2Face*, and *FaceShifter* (0.973, 0.973, 0.946), whereas GA yields the best results on *FaceSwap* and *NeuralTextures* (0.893 and 0.885). Under heavier compression in FF++ C40, GA and GA+LASSO dominate, particularly on *DeepFake* and *FaceShifter* (approximately 0.85 and 0.83). On Celeb-DF (C0), both GA and GA+LASSO again provide the strongest overall performance (around 0.88). Finally, in the most compressed setting, Celeb-DF C40, ExtraTrees delivers the best accuracy (approximately 0.673). These observations reinforce the complementary strengths of GA, GA+LASSO, and tree-based models under different compression regimes.

### Complete set of feature-selection tables

To support the detailed analysis of feature-selection strategies, Tables 12, 13, 14, 15, 16, 17, 18, 19, 20, 21, 22, 23, 24, 25, 26, 27, 28, 29, 30, 31, 32, 33, 34 and 35 present the complete results obtained using GA, ExtraTrees, K-Best, and RFE methods, with and without Lasso regularization, across both compression levels (C40 and C23) of the FF++ dataset. Each table reports the classification performance in terms of Accuracy, AUC, F1-score, Precision, and Recall (all expressed with their confidence intervals), as well as the computational time (in seconds). These results provide deeper insight into the trade-off between performance and computational complexity for each feature-selection configuration.

**Table 12:** Performance Results for ga Feature Selection (FF++ c40 dataset).

**Classifier**	**Manipulation**	**Acc (%)**	**AUC**	**F1**	**Precision**	**Recall**	**Time (s)**
*AdaBoost*							
	Deepfakes	$71.20 \pm 0.72$	$0.788 \pm 0.004$	$0.710 \pm 0.002$	$0.716 \pm 0.022$	$0.704 \pm 0.026$	37.25
	Face2Face	$64.30 \pm 2.59$	$0.702 \pm 0.030$	$0.631 \pm 0.019$	$0.653 \pm 0.035$	$0.610 \pm 0.015$	36.67
	FaceShifter	$72.91 \pm 2.04$	$0.807 \pm 0.025$	$0.724 \pm 0.024$	$0.737 \pm 0.017$	$0.712 \pm 0.034$	37.07
	FaceSwap	$65.08 \pm 2.04$	$0.705 \pm 0.035$	$0.643 \pm 0.031$	$0.658 \pm 0.022$	$0.629 \pm 0.058$	38.04
	NeuralTextures	$61.59 \pm 1.60$	$0.664 \pm 0.010$	$0.609 \pm 0.005$	$0.620 \pm 0.028$	$0.599 \pm 0.033$	37.42
*DecisionTree*							
	Deepfakes	$62.20 \pm 1.15$	$0.622 \pm 0.011$	$0.619 \pm 0.011$	$0.624 \pm 0.012$	$0.613 \pm 0.013$	8.17
	Face2Face	$57.04 \pm 3.00$	$0.570 \pm 0.030$	$0.568 \pm 0.043$	$0.571 \pm 0.028$	$0.564 \pm 0.062$	8.18
	FaceShifter	$62.17 \pm 1.51$	$0.622 \pm 0.015$	$0.617 \pm 0.008$	$0.624 \pm 0.021$	$0.610 \pm 0.005$	8.47
	FaceSwap	$56.99 \pm 0.34$	$0.570 \pm 0.003$	$0.565 \pm 0.008$	$0.572 \pm 0.002$	$0.558 \pm 0.014$	8.12
	NeuralTextures	$54.02 \pm 1.00$	$0.540 \pm 0.010$	$0.539 \pm 0.019$	$0.540 \pm 0.010$	$0.539 \pm 0.036$	7.98
*ExtraTrees*							
	Deepfakes	$76.76 \pm 1.20$	$0.851 \pm 0.003$	$0.764 \pm 0.018$	$0.776 \pm 0.002$	$0.753 \pm 0.037$	3.49
	Face2Face	$66.89 \pm 0.96$	$0.733 \pm 0.005$	$0.662 \pm 0.013$	$0.676 \pm 0.011$	$0.648 \pm 0.024$	3.68
	FaceShifter	$76.12 \pm 1.20$	$0.847 \pm 0.015$	$0.756 \pm 0.016$	$0.772 \pm 0.009$	$0.741 \pm 0.030$	3.46
	FaceSwap	$72.40 \pm 0.92$	$0.798 \pm 0.004$	$0.713 \pm 0.015$	$0.743 \pm 0.011$	$0.686 \pm 0.029$	3.77
	NeuralTextures	$60.50 \pm 2.68$	$0.657 \pm 0.010$	$0.601 \pm 0.023$	$0.607 \pm 0.032$	$0.596 \pm 0.029$	3.91
*GradientBoosting*							
	Deepfakes	$76.80 \pm 1.96$	$0.846 \pm 0.011$	$0.768 \pm 0.016$	$0.768 \pm 0.027$	$0.768 \pm 0.006$	199.98
	Face2Face	$68.19 \pm 1.89$	$0.751 \pm 0.013$	$0.675 \pm 0.016$	$0.690 \pm 0.026$	$0.662 \pm 0.018$	196.55
	FaceShifter	$76.73 \pm 0.62$	$0.849 \pm 0.008$	$0.764 \pm 0.006$	$0.774 \pm 0.010$	$0.755 \pm 0.011$	197.88
	FaceSwap	$70.14 \pm 2.49$	$0.772 \pm 0.028$	$0.697 \pm 0.029$	$0.708 \pm 0.021$	$0.686 \pm 0.041$	204.95
	NeuralTextures	$63.96 \pm 2.82$	$0.697 \pm 0.019$	$0.640 \pm 0.023$	$0.639 \pm 0.033$	$0.640 \pm 0.019$	201.60
*KNN*							
	Deepfakes	$72.23 \pm 0.84$	$0.789 \pm 0.013$	$0.735 \pm 0.007$	$0.703 \pm 0.015$	$0.770 \pm 0.022$	0.01
	Face2Face	$54.92 \pm 1.16$	$0.568 \pm 0.005$	$0.515 \pm 0.016$	$0.558 \pm 0.020$	$0.478 \pm 0.040$	0.01
	FaceShifter	$67.09 \pm 1.66$	$0.725 \pm 0.011$	$0.653 \pm 0.023$	$0.690 \pm 0.017$	$0.621 \pm 0.035$	0.02
	FaceSwap	$65.65 \pm 1.82$	$0.707 \pm 0.024$	$0.657 \pm 0.007$	$0.657 \pm 0.031$	$0.657 \pm 0.026$	0.02
	NeuralTextures	$50.05 \pm 3.24$	$0.504 \pm 0.025$	$0.474 \pm 0.042$	$0.500 \pm 0.036$	$0.451 \pm 0.048$	0.02
*Logistic*							
	Deepfakes	$78.21 \pm 1.98$	$0.861 \pm 0.018$	$0.783 \pm 0.015$	$0.780 \pm 0.031$	$0.786 \pm 0.008$	4.20
	Face2Face	$72.13 \pm 2.08$	$0.793 \pm 0.015$	$0.720 \pm 0.027$	$0.724 \pm 0.013$	$0.716 \pm 0.044$	3.50
	FaceShifter	$79.12 \pm 0.22$	$0.869 \pm 0.004$	$0.790 \pm 0.003$	$0.795 \pm 0.003$	$0.785 \pm 0.007$	3.66
	FaceSwap	$70.66 \pm 0.58$	$0.780 \pm 0.025$	$0.705 \pm 0.009$	$0.709 \pm 0.005$	$0.700 \pm 0.019$	3.93
	NeuralTextures	$65.83 \pm 0.65$	$0.713 \pm 0.015$	$0.657 \pm 0.006$	$0.659 \pm 0.009$	$0.656 \pm 0.009$	3.54
*MLP*							
	Deepfakes	$85.16 \pm 0.80$	$0.926 \pm 0.012$	$0.851 \pm 0.009$	$0.854 \pm 0.008$	$0.848 \pm 0.019$	7.42
	Face2Face	$75.69 \pm 1.86$	$0.832 \pm 0.024$	$0.754 \pm 0.020$	$0.763 \pm 0.019$	$0.746 \pm 0.026$	8.45
	FaceShifter	$83.78 \pm 0.59$	$0.915 \pm 0.005$	$0.837 \pm 0.011$	$0.840 \pm 0.015$	$0.834 \pm 0.035$	6.44
	FaceSwap	$78.28 \pm 2.08$	$0.858 \pm 0.024$	$0.784 \pm 0.020$	$0.781 \pm 0.025$	$0.786 \pm 0.024$	7.33
	NeuralTextures	$68.72 \pm 2.29$	$0.747 \pm 0.021$	$0.689 \pm 0.014$	$0.685 \pm 0.036$	$0.692 \pm 0.022$	9.06
*RandomForest*							
	Deepfakes	$75.83 \pm 1.66$	$0.840 \pm 0.009$	$0.757 \pm 0.019$	$0.762 \pm 0.016$	$0.751 \pm 0.027$	15.60
	Face2Face	$66.51 \pm 1.86$	$0.731 \pm 0.015$	$0.654 \pm 0.016$	$0.677 \pm 0.023$	$0.632 \pm 0.010$	15.76
	FaceShifter	$76.03 \pm 0.55$	$0.844 \pm 0.008$	$0.756 \pm 0.010$	$0.769 \pm 0.009$	$0.745 \pm 0.026$	15.53
	FaceSwap	$70.54 \pm 2.95$	$0.775 \pm 0.020$	$0.699 \pm 0.033$	$0.716 \pm 0.029$	$0.682 \pm 0.040$	16.52
	NeuralTextures	$61.74 \pm 1.32$	$0.666 \pm 0.016$	$0.610 \pm 0.028$	$0.621 \pm 0.005$	$0.600 \pm 0.050$	16.52
*SVM-RBF*							
	Deepfakes	$82.56 \pm 2.13$	$0.904 \pm 0.006$	$0.824 \pm 0.024$	$0.831 \pm 0.016$	$0.817 \pm 0.035$	121.64
	Face2Face	$74.19 \pm 2.66$	$0.824 \pm 0.023$	$0.740 \pm 0.024$	$0.745 \pm 0.043$	$0.736 \pm 0.043$	136.32
	FaceShifter	$82.77 \pm 0.49$	$0.908 \pm 0.001$	$0.826 \pm 0.003$	$0.834 \pm 0.019$	$0.818 \pm 0.019$	118.09
	FaceSwap	$76.23 \pm 2.12$	$0.842 \pm 0.031$	$0.763 \pm 0.018$	$0.761 \pm 0.029$	$0.765 \pm 0.010$	140.07
	NeuralTextures	$66.57 \pm 0.63$	$0.737 \pm 0.012$	$0.664 \pm 0.011$	$0.667 \pm 0.016$	$0.661 \pm 0.035$	145.08
*XGBoost*							
	Deepfakes	$81.03 \pm 1.49$	$0.892 \pm 0.014$	$0.811 \pm 0.011$	$0.809 \pm 0.028$	$0.812 \pm 0.012$	4.49
	Face2Face	$70.56 \pm 2.23$	$0.780 \pm 0.009$	$0.704 \pm 0.016$	$0.709 \pm 0.036$	$0.699 \pm 0.027$	4.74
	FaceShifter	$79.89 \pm 0.08$	$0.882 \pm 0.008$	$0.795 \pm 0.007$	$0.811 \pm 0.021$	$0.780 \pm 0.032$	4.38
	FaceSwap	$73.25 \pm 0.39$	$0.811 \pm 0.005$	$0.731 \pm 0.005$	$0.735 \pm 0.008$	$0.728 \pm 0.014$	4.78
	NeuralTextures	$64.04 \pm 0.71$	$0.697 \pm 0.008$	$0.644 \pm 0.012$	$0.638 \pm 0.021$	$0.650 \pm 0.046$	4.92
**Average**		**70.11**	**0.762**	**0.697**	−	−	−

Method: ga.

Values shown as Mean ± 95% Confidence Interval.

FaceForensics++ C40 compression level.

**Table 13:** Performance Results for ga+lasso Feature Selection (FF++ c40 dataset).

**Classifier**	**Manipulation**	**Acc (%)**	**AUC**	**F1**	**Precision**	**Recall**	**Time (s)**
*AdaBoost*							
	Deepfakes	$71.26 \pm 0.41$	$0.792 \pm 0.011$	$0.710 \pm 0.012$	$0.716 \pm 0.015$	$0.704 \pm 0.037$	21.87
	Face2Face	$64.77 \pm 2.57$	$0.700 \pm 0.029$	$0.640 \pm 0.027$	$0.654 \pm 0.026$	$0.626 \pm 0.027$	20.53
	FaceShifter	$72.58 \pm 2.49$	$0.803 \pm 0.020$	$0.724 \pm 0.028$	$0.728 \pm 0.020$	$0.720 \pm 0.037$	20.81
	FaceSwap	$64.80 \pm 2.40$	$0.706 \pm 0.026$	$0.642 \pm 0.051$	$0.653 \pm 0.011$	$0.632 \pm 0.101$	18.90
	NeuralTextures	$62.18 \pm 1.56$	$0.669 \pm 0.019$	$0.622 \pm 0.032$	$0.621 \pm 0.008$	$0.623 \pm 0.061$	12.69
*DecisionTree*							
	Deepfakes	$61.71 \pm 1.34$	$0.617 \pm 0.013$	$0.615 \pm 0.009$	$0.619 \pm 0.019$	$0.611 \pm 0.020$	4.65
	Face2Face	$58.06 \pm 1.89$	$0.581 \pm 0.019$	$0.576 \pm 0.014$	$0.582 \pm 0.021$	$0.570 \pm 0.010$	4.36
	FaceShifter	$61.51 \pm 1.55$	$0.615 \pm 0.016$	$0.614 \pm 0.013$	$0.616 \pm 0.017$	$0.612 \pm 0.010$	4.52
	FaceSwap	$57.01 \pm 1.73$	$0.570 \pm 0.017$	$0.568 \pm 0.023$	$0.571 \pm 0.017$	$0.565 \pm 0.036$	4.01
	NeuralTextures	$54.58 \pm 1.59$	$0.546 \pm 0.016$	$0.550 \pm 0.026$	$0.544 \pm 0.017$	$0.556 \pm 0.051$	2.65
*ExtraTrees*							
	Deepfakes	$77.59 \pm 0.54$	$0.860 \pm 0.005$	$0.773 \pm 0.002$	$0.784 \pm 0.015$	$0.762 \pm 0.011$	2.49
	Face2Face	$67.02 \pm 2.24$	$0.741 \pm 0.034$	$0.661 \pm 0.030$	$0.680 \pm 0.016$	$0.642 \pm 0.041$	2.58
	FaceShifter	$77.26 \pm 1.24$	$0.852 \pm 0.008$	$0.768 \pm 0.013$	$0.783 \pm 0.015$	$0.755 \pm 0.016$	2.40
	FaceSwap	$72.35 \pm 1.30$	$0.798 \pm 0.014$	$0.715 \pm 0.014$	$0.739 \pm 0.022$	$0.692 \pm 0.027$	2.41
	NeuralTextures	$62.28 \pm 1.60$	$0.677 \pm 0.020$	$0.619 \pm 0.012$	$0.625 \pm 0.028$	$0.613 \pm 0.038$	1.87
*GradientBoosting*							
	Deepfakes	$76.37 \pm 0.72$	$0.845 \pm 0.007$	$0.764 \pm 0.010$	$0.763 \pm 0.006$	$0.765 \pm 0.020$	117.50
	Face2Face	$68.13 \pm 0.50$	$0.747 \pm 0.008$	$0.677 \pm 0.007$	$0.686 \pm 0.003$	$0.668 \pm 0.012$	109.65
	FaceShifter	$76.75 \pm 0.50$	$0.848 \pm 0.011$	$0.764 \pm 0.008$	$0.775 \pm 0.003$	$0.753 \pm 0.017$	111.50
	FaceSwap	$69.21 \pm 2.19$	$0.765 \pm 0.026$	$0.690 \pm 0.023$	$0.696 \pm 0.021$	$0.683 \pm 0.026$	101.80
	NeuralTextures	$64.02 \pm 1.72$	$0.697 \pm 0.018$	$0.641 \pm 0.021$	$0.639 \pm 0.019$	$0.644 \pm 0.037$	68.09
*KNN*							
	Deepfakes	$73.54 \pm 1.38$	$0.808 \pm 0.009$	$0.745 \pm 0.011$	$0.719 \pm 0.016$	$0.773 \pm 0.008$	0.01
	Face2Face	$55.71 \pm 0.27$	$0.580 \pm 0.008$	$0.522 \pm 0.025$	$0.567 \pm 0.011$	$0.484 \pm 0.052$	0.01
	FaceShifter	$68.31 \pm 1.95$	$0.739 \pm 0.011$	$0.666 \pm 0.023$	$0.704 \pm 0.019$	$0.631 \pm 0.028$	0.01
	FaceSwap	$67.30 \pm 1.60$	$0.729 \pm 0.022$	$0.674 \pm 0.015$	$0.672 \pm 0.021$	$0.676 \pm 0.026$	0.01
	NeuralTextures	$51.00 \pm 3.28$	$0.519 \pm 0.032$	$0.480 \pm 0.037$	$0.511 \pm 0.038$	$0.452 \pm 0.049$	0.01
*Logistic*							
	Deepfakes	$78.68 \pm 2.80$	$0.867 \pm 0.019$	$0.786 \pm 0.028$	$0.788 \pm 0.028$	$0.784 \pm 0.029$	2.43
	Face2Face	$72.73 \pm 3.03$	$0.797 \pm 0.016$	$0.725 \pm 0.034$	$0.731 \pm 0.024$	$0.719 \pm 0.044$	2.22
	FaceShifter	$79.28 \pm 1.20$	$0.875 \pm 0.002$	$0.791 \pm 0.009$	$0.797 \pm 0.023$	$0.785 \pm 0.011$	2.52
	FaceSwap	$71.09 \pm 1.82$	$0.784 \pm 0.023$	$0.707 \pm 0.022$	$0.717 \pm 0.014$	$0.698 \pm 0.029$	2.09
	NeuralTextures	$66.66 \pm 0.21$	$0.723 \pm 0.012$	$0.663 \pm 0.008$	$0.669 \pm 0.011$	$0.658 \pm 0.027$	1.57
*MLP*							
	Deepfakes	$84.48 \pm 0.78$	$0.917 \pm 0.017$	$0.845 \pm 0.011$	$0.846 \pm 0.008$	$0.843 \pm 0.030$	5.43
	Face2Face	$75.73 \pm 1.47$	$0.828 \pm 0.017$	$0.756 \pm 0.009$	$0.760 \pm 0.028$	$0.753 \pm 0.013$	6.83
	FaceShifter	$83.60 \pm 0.44$	$0.910 \pm 0.006$	$0.834 \pm 0.004$	$0.845 \pm 0.007$	$0.822 \pm 0.003$	4.41
	FaceSwap	$77.14 \pm 3.80$	$0.848 \pm 0.035$	$0.771 \pm 0.042$	$0.773 \pm 0.030$	$0.769 \pm 0.054$	5.05
	NeuralTextures	$66.52 \pm 0.68$	$0.721 \pm 0.008$	$0.663 \pm 0.016$	$0.666 \pm 0.007$	$0.660 \pm 0.036$	5.27
*RandomForest*							
	Deepfakes	$76.56 \pm 1.61$	$0.848 \pm 0.014$	$0.762 \pm 0.015$	$0.774 \pm 0.029$	$0.751 \pm 0.027$	12.22
	Face2Face	$67.29 \pm 1.25$	$0.737 \pm 0.004$	$0.663 \pm 0.016$	$0.684 \pm 0.011$	$0.643 \pm 0.022$	11.81
	FaceShifter	$76.62 \pm 1.37$	$0.847 \pm 0.017$	$0.763 \pm 0.016$	$0.774 \pm 0.010$	$0.752 \pm 0.021$	11.40
	FaceSwap	$70.73 \pm 3.07$	$0.779 \pm 0.021$	$0.700 \pm 0.031$	$0.718 \pm 0.034$	$0.683 \pm 0.029$	11.69
	NeuralTextures	$62.26 \pm 2.25$	$0.675 \pm 0.019$	$0.616 \pm 0.011$	$0.627 \pm 0.032$	$0.605 \pm 0.008$	9.28
*SVM-RBF*							
	Deepfakes	$82.98 \pm 2.76$	$0.906 \pm 0.009$	$0.828 \pm 0.028$	$0.836 \pm 0.029$	$0.821 \pm 0.028$	69.77
	Face2Face	$74.80 \pm 3.01$	$0.828 \pm 0.026$	$0.746 \pm 0.036$	$0.751 \pm 0.029$	$0.741 \pm 0.059$	76.53
	FaceShifter	$82.76 \pm 0.54$	$0.909 \pm 0.003$	$0.826 \pm 0.008$	$0.833 \pm 0.014$	$0.819 \pm 0.027$	65.68
	FaceSwap	$76.31 \pm 3.25$	$0.842 \pm 0.035$	$0.764 \pm 0.031$	$0.762 \pm 0.036$	$0.765 \pm 0.028$	69.67
	NeuralTextures	$67.52 \pm 1.10$	$0.739 \pm 0.001$	$0.675 \pm 0.023$	$0.675 \pm 0.004$	$0.675 \pm 0.049$	50.87
*XGBoost*							
	Deepfakes	$80.39 \pm 0.95$	$0.886 \pm 0.017$	$0.804 \pm 0.010$	$0.805 \pm 0.012$	$0.802 \pm 0.014$	2.42
	Face2Face	$70.99 \pm 0.41$	$0.781 \pm 0.012$	$0.710 \pm 0.010$	$0.710 \pm 0.024$	$0.709 \pm 0.045$	2.67
	FaceShifter	$79.64 \pm 1.15$	$0.881 \pm 0.007$	$0.793 \pm 0.009$	$0.808 \pm 0.021$	$0.778 \pm 0.003$	2.33
	FaceSwap	$73.65 \pm 0.34$	$0.810 \pm 0.004$	$0.735 \pm 0.002$	$0.741 \pm 0.006$	$0.728 \pm 0.003$	2.11
	NeuralTextures	$64.65 \pm 1.03$	$0.703 \pm 0.008$	$0.645 \pm 0.018$	$0.647 \pm 0.014$	$0.643 \pm 0.040$	1.49
**Average**		**70.37**	**0.765**	**0.700**	−	−	−

Method: ga+lasso.

Values shown as Mean ± 95% Confidence Interval.

FaceForensics++ C40 compression level.

**Table 14:** Performance Results for extratrees Feature Selection (FF++ c40 dataset).

**Classifier**	**Manipulation**	**Acc (%)**	**AUC**	**F1**	**Precision**	**Recall**	**Time (s)**
*AdaBoost*							
	Deepfakes	$69.82 \pm 0.75$	$0.777 \pm 0.012$	$0.697 \pm 0.004$	$0.699 \pm 0.019$	$0.696 \pm 0.023$	2.29
	Face2Face	$64.08 \pm 2.57$	$0.693 \pm 0.014$	$0.633 \pm 0.028$	$0.647 \pm 0.025$	$0.619 \pm 0.032$	2.32
	FaceShifter	$73.20 \pm 0.94$	$0.805 \pm 0.017$	$0.731 \pm 0.012$	$0.733 \pm 0.014$	$0.730 \pm 0.027$	2.30
	FaceSwap	$65.16 \pm 2.07$	$0.709 \pm 0.017$	$0.644 \pm 0.033$	$0.659 \pm 0.012$	$0.630 \pm 0.052$	2.31
	NeuralTextures	$62.38 \pm 1.81$	$0.672 \pm 0.014$	$0.616 \pm 0.032$	$0.628 \pm 0.021$	$0.606 \pm 0.064$	2.26
*DecisionTree*							
	Deepfakes	$63.91 \pm 1.54$	$0.639 \pm 0.015$	$0.637 \pm 0.017$	$0.641 \pm 0.017$	$0.632 \pm 0.024$	0.45
	Face2Face	$56.69 \pm 0.85$	$0.567 \pm 0.008$	$0.566 \pm 0.006$	$0.567 \pm 0.010$	$0.564 \pm 0.009$	0.48
	FaceShifter	$64.24 \pm 1.21$	$0.642 \pm 0.012$	$0.640 \pm 0.016$	$0.644 \pm 0.010$	$0.636 \pm 0.022$	0.45
	FaceSwap	$57.39 \pm 2.15$	$0.574 \pm 0.021$	$0.572 \pm 0.017$	$0.575 \pm 0.024$	$0.569 \pm 0.021$	0.45
	NeuralTextures	$54.58 \pm 1.82$	$0.546 \pm 0.018$	$0.547 \pm 0.027$	$0.545 \pm 0.016$	$0.550 \pm 0.037$	0.45
*ExtraTrees*							
	Deepfakes	$75.76 \pm 1.29$	$0.842 \pm 0.007$	$0.757 \pm 0.008$	$0.758 \pm 0.024$	$0.757 \pm 0.012$	0.86
	Face2Face	$68.04 \pm 5.21$	$0.747 \pm 0.031$	$0.670 \pm 0.061$	$0.692 \pm 0.047$	$0.650 \pm 0.074$	0.90
	FaceShifter	$77.07 \pm 0.67$	$0.853 \pm 0.010$	$0.765 \pm 0.011$	$0.784 \pm 0.013$	$0.747 \pm 0.028$	0.84
	FaceSwap	$71.24 \pm 3.32$	$0.788 \pm 0.038$	$0.702 \pm 0.032$	$0.729 \pm 0.039$	$0.676 \pm 0.026$	0.91
	NeuralTextures	$63.67 \pm 2.44$	$0.693 \pm 0.021$	$0.632 \pm 0.032$	$0.640 \pm 0.028$	$0.624 \pm 0.058$	0.93
*GradientBoosting*							
	Deepfakes	$73.89 \pm 2.28$	$0.818 \pm 0.012$	$0.739 \pm 0.019$	$0.740 \pm 0.029$	$0.737 \pm 0.011$	12.22
	Face2Face	$66.02 \pm 3.03$	$0.725 \pm 0.026$	$0.652 \pm 0.036$	$0.668 \pm 0.028$	$0.638 \pm 0.044$	12.16
	FaceShifter	$74.90 \pm 1.63$	$0.831 \pm 0.014$	$0.746 \pm 0.018$	$0.756 \pm 0.014$	$0.736 \pm 0.021$	12.15
	FaceSwap	$68.21 \pm 3.01$	$0.753 \pm 0.018$	$0.678 \pm 0.036$	$0.687 \pm 0.024$	$0.670 \pm 0.048$	12.25
	NeuralTextures	$63.94 \pm 1.53$	$0.695 \pm 0.010$	$0.638 \pm 0.029$	$0.640 \pm 0.005$	$0.637 \pm 0.054$	12.00
*KNN*							
	Deepfakes	$78.80 \pm 3.20$	$0.861 \pm 0.018$	$0.783 \pm 0.037$	$0.801 \pm 0.039$	$0.766 \pm 0.062$	0.00
	Face2Face	$62.70 \pm 2.19$	$0.674 \pm 0.008$	$0.596 \pm 0.022$	$0.650 \pm 0.027$	$0.551 \pm 0.019$	0.00
	FaceShifter	$75.89 \pm 1.82$	$0.831 \pm 0.006$	$0.748 \pm 0.018$	$0.782 \pm 0.024$	$0.717 \pm 0.017$	0.00
	FaceSwap	$71.76 \pm 2.73$	$0.785 \pm 0.048$	$0.707 \pm 0.028$	$0.735 \pm 0.029$	$0.682 \pm 0.027$	0.00
	NeuralTextures	$55.37 \pm 0.91$	$0.577 \pm 0.002$	$0.518 \pm 0.036$	$0.562 \pm 0.006$	$0.481 \pm 0.059$	0.00
*Logistic*							
	Deepfakes	$73.16 \pm 0.10$	$0.804 \pm 0.008$	$0.732 \pm 0.003$	$0.731 \pm 0.006$	$0.733 \pm 0.012$	0.87
	Face2Face	$65.99 \pm 2.96$	$0.715 \pm 0.027$	$0.653 \pm 0.033$	$0.666 \pm 0.029$	$0.641 \pm 0.037$	0.83
	FaceShifter	$74.80 \pm 1.21$	$0.825 \pm 0.019$	$0.746 \pm 0.015$	$0.751 \pm 0.011$	$0.741 \pm 0.028$	0.85
	FaceSwap	$66.97 \pm 0.88$	$0.736 \pm 0.025$	$0.669 \pm 0.012$	$0.670 \pm 0.008$	$0.668 \pm 0.019$	0.56
	NeuralTextures	$64.25 \pm 1.89$	$0.695 \pm 0.005$	$0.640 \pm 0.034$	$0.644 \pm 0.015$	$0.635 \pm 0.065$	0.48
*MLP*							
	Deepfakes	$76.23 \pm 2.46$	$0.841 \pm 0.024$	$0.763 \pm 0.027$	$0.760 \pm 0.020$	$0.767 \pm 0.034$	10.46
	Face2Face	$64.33 \pm 1.29$	$0.697 \pm 0.008$	$0.646 \pm 0.022$	$0.641 \pm 0.007$	$0.651 \pm 0.040$	17.35
	FaceShifter	$75.08 \pm 1.80$	$0.825 \pm 0.026$	$0.752 \pm 0.023$	$0.748 \pm 0.015$	$0.756 \pm 0.042$	14.15
	FaceSwap	$69.82 \pm 3.55$	$0.763 \pm 0.038$	$0.696 \pm 0.045$	$0.701 \pm 0.025$	$0.690 \pm 0.067$	17.10
	NeuralTextures	$59.96 \pm 0.72$	$0.634 \pm 0.015$	$0.602 \pm 0.017$	$0.598 \pm 0.004$	$0.607 \pm 0.034$	19.46
*RandomForest*							
	Deepfakes	$75.02 \pm 1.29$	$0.831 \pm 0.007$	$0.750 \pm 0.006$	$0.751 \pm 0.028$	$0.748 \pm 0.016$	4.11
	Face2Face	$67.37 \pm 2.08$	$0.738 \pm 0.023$	$0.663 \pm 0.026$	$0.685 \pm 0.018$	$0.643 \pm 0.036$	4.34
	FaceShifter	$75.91 \pm 0.82$	$0.839 \pm 0.021$	$0.753 \pm 0.009$	$0.771 \pm 0.008$	$0.736 \pm 0.009$	4.11
	FaceSwap	$70.55 \pm 2.92$	$0.777 \pm 0.033$	$0.699 \pm 0.032$	$0.715 \pm 0.027$	$0.684 \pm 0.037$	4.32
	NeuralTextures	$64.15 \pm 0.85$	$0.693 \pm 0.014$	$0.638 \pm 0.015$	$0.643 \pm 0.015$	$0.634 \pm 0.038$	4.35
*SVM-RBF*							
	Deepfakes	$78.47 \pm 1.11$	$0.863 \pm 0.005$	$0.786 \pm 0.019$	$0.782 \pm 0.016$	$0.790 \pm 0.050$	11.02
	Face2Face	$68.96 \pm 2.49$	$0.754 \pm 0.023$	$0.679 \pm 0.027$	$0.702 \pm 0.025$	$0.658 \pm 0.030$	13.27
	FaceShifter	$78.36 \pm 1.22$	$0.861 \pm 0.017$	$0.776 \pm 0.016$	$0.804 \pm 0.007$	$0.750 \pm 0.030$	10.26
	FaceSwap	$73.72 \pm 2.71$	$0.810 \pm 0.027$	$0.736 \pm 0.026$	$0.740 \pm 0.030$	$0.731 \pm 0.022$	12.93
	NeuralTextures	$64.89 \pm 0.70$	$0.702 \pm 0.001$	$0.649 \pm 0.021$	$0.648 \pm 0.007$	$0.649 \pm 0.048$	13.79
*XGBoost*							
	Deepfakes	$76.29 \pm 0.13$	$0.846 \pm 0.010$	$0.764 \pm 0.005$	$0.761 \pm 0.012$	$0.766 \pm 0.023$	0.30
	Face2Face	$67.63 \pm 3.39$	$0.740 \pm 0.032$	$0.674 \pm 0.033$	$0.679 \pm 0.036$	$0.670 \pm 0.031$	0.33
	FaceShifter	$76.62 \pm 1.94$	$0.846 \pm 0.017$	$0.762 \pm 0.020$	$0.776 \pm 0.020$	$0.749 \pm 0.020$	0.27
	FaceSwap	$70.92 \pm 1.60$	$0.775 \pm 0.018$	$0.706 \pm 0.018$	$0.715 \pm 0.015$	$0.697 \pm 0.022$	0.40
	NeuralTextures	$63.40 \pm 0.74$	$0.680 \pm 0.010$	$0.634 \pm 0.018$	$0.633 \pm 0.019$	$0.635 \pm 0.053$	0.31
**Average**		**68.83**	**0.748**	**0.684**	−	−	−

Method: extratrees.

Values shown as Mean ± 95% Confidence Interval.

FaceForensics++ C40 compression level.

**Table 15:** Performance Results for extratrees+lasso Feature Selection (FF++ c40 dataset).

**Classifier**	**Manipulation**	**Acc (%)**	**AUC**	**F1**	**Precision**	**Recall**	**Time (s)**
*AdaBoost*							
	Deepfakes	$69.97 \pm 0.41$	$0.778 \pm 0.008$	$0.697 \pm 0.005$	$0.703 \pm 0.015$	$0.691 \pm 0.024$	2.09
	Face2Face	$63.83 \pm 1.58$	$0.692 \pm 0.010$	$0.630 \pm 0.018$	$0.645 \pm 0.015$	$0.616 \pm 0.020$	2.00
	FaceShifter	$73.20 \pm 1.67$	$0.806 \pm 0.017$	$0.733 \pm 0.011$	$0.730 \pm 0.030$	$0.737 \pm 0.025$	2.00
	FaceSwap	$65.31 \pm 2.18$	$0.706 \pm 0.013$	$0.647 \pm 0.040$	$0.658 \pm 0.009$	$0.637 \pm 0.072$	2.11
	NeuralTextures	$62.10 \pm 0.76$	$0.670 \pm 0.010$	$0.617 \pm 0.026$	$0.623 \pm 0.018$	$0.612 \pm 0.065$	2.00
*DecisionTree*							
	Deepfakes	$63.91 \pm 2.72$	$0.639 \pm 0.027$	$0.638 \pm 0.023$	$0.640 \pm 0.031$	$0.637 \pm 0.015$	0.40
	Face2Face	$57.29 \pm 0.10$	$0.573 \pm 0.001$	$0.571 \pm 0.008$	$0.573 \pm 0.002$	$0.569 \pm 0.018$	0.40
	FaceShifter	$63.47 \pm 2.11$	$0.635 \pm 0.021$	$0.633 \pm 0.027$	$0.636 \pm 0.020$	$0.631 \pm 0.042$	0.38
	FaceSwap	$57.63 \pm 1.10$	$0.576 \pm 0.011$	$0.574 \pm 0.005$	$0.578 \pm 0.014$	$0.569 \pm 0.007$	0.41
	NeuralTextures	$54.50 \pm 1.04$	$0.545 \pm 0.010$	$0.542 \pm 0.025$	$0.545 \pm 0.011$	$0.540 \pm 0.048$	0.40
*ExtraTrees*							
	Deepfakes	$75.73 \pm 2.20$	$0.844 \pm 0.014$	$0.757 \pm 0.018$	$0.759 \pm 0.031$	$0.755 \pm 0.006$	0.79
	Face2Face	$67.73 \pm 2.41$	$0.746 \pm 0.021$	$0.666 \pm 0.029$	$0.690 \pm 0.021$	$0.643 \pm 0.037$	0.84
	FaceShifter	$77.04 \pm 0.91$	$0.852 \pm 0.009$	$0.764 \pm 0.013$	$0.786 \pm 0.005$	$0.743 \pm 0.027$	0.77
	FaceSwap	$70.62 \pm 3.62$	$0.783 \pm 0.038$	$0.697 \pm 0.040$	$0.719 \pm 0.034$	$0.676 \pm 0.046$	0.84
	NeuralTextures	$64.13 \pm 1.08$	$0.695 \pm 0.014$	$0.636 \pm 0.033$	$0.645 \pm 0.008$	$0.629 \pm 0.073$	0.87
*GradientBoosting*							
	Deepfakes	$73.73 \pm 2.91$	$0.818 \pm 0.018$	$0.737 \pm 0.027$	$0.737 \pm 0.032$	$0.737 \pm 0.024$	11.09
	Face2Face	$66.18 \pm 1.68$	$0.723 \pm 0.019$	$0.654 \pm 0.024$	$0.670 \pm 0.011$	$0.639 \pm 0.037$	10.56
	FaceShifter	$74.83 \pm 2.38$	$0.831 \pm 0.019$	$0.745 \pm 0.020$	$0.755 \pm 0.033$	$0.736 \pm 0.010$	10.56
	FaceSwap	$68.06 \pm 1.66$	$0.752 \pm 0.023$	$0.677 \pm 0.018$	$0.685 \pm 0.017$	$0.668 \pm 0.022$	11.26
	NeuralTextures	$64.04 \pm 2.27$	$0.694 \pm 0.022$	$0.640 \pm 0.035$	$0.640 \pm 0.013$	$0.640 \pm 0.059$	10.59
*KNN*							
	Deepfakes	$78.50 \pm 1.98$	$0.858 \pm 0.024$	$0.781 \pm 0.023$	$0.796 \pm 0.022$	$0.767 \pm 0.038$	0.00
	Face2Face	$63.42 \pm 1.59$	$0.679 \pm 0.003$	$0.609 \pm 0.012$	$0.655 \pm 0.025$	$0.569 \pm 0.014$	0.00
	FaceShifter	$76.02 \pm 2.14$	$0.828 \pm 0.010$	$0.749 \pm 0.022$	$0.784 \pm 0.027$	$0.717 \pm 0.021$	0.00
	FaceSwap	$71.84 \pm 4.45$	$0.780 \pm 0.054$	$0.707 \pm 0.047$	$0.737 \pm 0.047$	$0.679 \pm 0.047$	0.00
	NeuralTextures	$55.96 \pm 2.16$	$0.580 \pm 0.028$	$0.526 \pm 0.061$	$0.568 \pm 0.014$	$0.491 \pm 0.094$	0.00
*Logistic*							
	Deepfakes	$73.02 \pm 0.46$	$0.804 \pm 0.008$	$0.731 \pm 0.003$	$0.729 \pm 0.008$	$0.733 \pm 0.006$	0.93
	Face2Face	$66.06 \pm 2.99$	$0.715 \pm 0.026$	$0.654 \pm 0.032$	$0.667 \pm 0.030$	$0.641 \pm 0.035$	0.69
	FaceShifter	$74.67 \pm 0.83$	$0.824 \pm 0.018$	$0.744 \pm 0.012$	$0.751 \pm 0.005$	$0.738 \pm 0.024$	0.73
	FaceSwap	$66.94 \pm 1.16$	$0.736 \pm 0.024$	$0.669 \pm 0.015$	$0.670 \pm 0.010$	$0.668 \pm 0.024$	0.86
	NeuralTextures	$64.09 \pm 1.85$	$0.695 \pm 0.003$	$0.639 \pm 0.034$	$0.642 \pm 0.017$	$0.637 \pm 0.068$	0.54
*MLP*							
	Deepfakes	$75.36 \pm 1.94$	$0.836 \pm 0.014$	$0.753 \pm 0.023$	$0.755 \pm 0.012$	$0.750 \pm 0.034$	15.36
	Face2Face	$64.60 \pm 1.69$	$0.699 \pm 0.021$	$0.643 \pm 0.015$	$0.649 \pm 0.020$	$0.636 \pm 0.013$	20.10
	FaceShifter	$74.20 \pm 1.08$	$0.821 \pm 0.010$	$0.743 \pm 0.009$	$0.741 \pm 0.016$	$0.744 \pm 0.011$	16.90
	FaceSwap	$68.93 \pm 2.71$	$0.759 \pm 0.024$	$0.688 \pm 0.027$	$0.691 \pm 0.030$	$0.686 \pm 0.036$	19.18
	NeuralTextures	$59.39 \pm 0.96$	$0.629 \pm 0.009$	$0.597 \pm 0.016$	$0.592 \pm 0.008$	$0.602 \pm 0.029$	21.75
*RandomForest*							
	Deepfakes	$75.46 \pm 3.25$	$0.835 \pm 0.022$	$0.755 \pm 0.027$	$0.753 \pm 0.043$	$0.758 \pm 0.011$	3.56
	Face2Face	$67.42 \pm 2.43$	$0.737 \pm 0.020$	$0.664 \pm 0.039$	$0.685 \pm 0.011$	$0.645 \pm 0.064$	3.75
	FaceShifter	$75.69 \pm 2.29$	$0.839 \pm 0.013$	$0.751 \pm 0.025$	$0.770 \pm 0.021$	$0.732 \pm 0.032$	3.53
	FaceSwap	$70.32 \pm 2.15$	$0.772 \pm 0.026$	$0.697 \pm 0.023$	$0.712 \pm 0.021$	$0.683 \pm 0.025$	3.92
	NeuralTextures	$64.42 \pm 1.50$	$0.694 \pm 0.011$	$0.639 \pm 0.019$	$0.648 \pm 0.016$	$0.631 \pm 0.032$	3.88
*SVM-RBF*							
	Deepfakes	$78.47 \pm 1.39$	$0.859 \pm 0.002$	$0.786 \pm 0.021$	$0.781 \pm 0.012$	$0.792 \pm 0.049$	12.60
	Face2Face	$68.68 \pm 2.13$	$0.749 \pm 0.012$	$0.677 \pm 0.020$	$0.699 \pm 0.025$	$0.656 \pm 0.017$	13.79
	FaceShifter	$78.14 \pm 1.49$	$0.857 \pm 0.018$	$0.773 \pm 0.018$	$0.804 \pm 0.010$	$0.744 \pm 0.028$	11.41
	FaceSwap	$73.13 \pm 3.23$	$0.808 \pm 0.029$	$0.730 \pm 0.030$	$0.734 \pm 0.038$	$0.726 \pm 0.026$	14.33
	NeuralTextures	$64.63 \pm 0.87$	$0.700 \pm 0.005$	$0.645 \pm 0.012$	$0.647 \pm 0.025$	$0.644 \pm 0.049$	15.18
*XGBoost*							
	Deepfakes	$76.48 \pm 1.21$	$0.848 \pm 0.006$	$0.766 \pm 0.012$	$0.763 \pm 0.015$	$0.769 \pm 0.018$	0.27
	Face2Face	$67.41 \pm 0.71$	$0.736 \pm 0.014$	$0.673 \pm 0.004$	$0.675 \pm 0.011$	$0.671 \pm 0.004$	0.23
	FaceShifter	$77.02 \pm 1.38$	$0.846 \pm 0.012$	$0.766 \pm 0.021$	$0.779 \pm 0.002$	$0.755 \pm 0.042$	0.28
	FaceSwap	$69.99 \pm 1.95$	$0.772 \pm 0.017$	$0.698 \pm 0.023$	$0.704 \pm 0.016$	$0.692 \pm 0.032$	0.29
	NeuralTextures	$62.28 \pm 2.56$	$0.675 \pm 0.022$	$0.624 \pm 0.041$	$0.621 \pm 0.016$	$0.626 \pm 0.069$	0.38
**Average**		**68.72**	**0.747**	**0.683**	−	−	−

Method: extratrees+lasso.

Values shown as Mean ± 95% Confidence Interval.

FaceForensics++ C40 compression level.

**Table 16:** Performance Results for kbest Feature Selection (FF++ c40 dataset).

**Classifier**	**Manipulation**	**Acc (%)**	**AUC**	**F1**	**Precision**	**Recall**	**Time (s)**
*AdaBoost*							
	Deepfakes	$69.46 \pm 2.43$	$0.774 \pm 0.022$	$0.690 \pm 0.018$	$0.701 \pm 0.038$	$0.680 \pm 0.022$	2.17
	Face2Face	$61.63 \pm 1.87$	$0.663 \pm 0.024$	$0.607 \pm 0.025$	$0.622 \pm 0.016$	$0.592 \pm 0.033$	2.03
	FaceShifter	$70.88 \pm 1.77$	$0.774 \pm 0.007$	$0.706 \pm 0.012$	$0.713 \pm 0.026$	$0.698 \pm 0.002$	2.09
	FaceSwap	$60.58 \pm 1.33$	$0.648 \pm 0.002$	$0.606 \pm 0.017$	$0.606 \pm 0.013$	$0.606 \pm 0.027$	1.96
	NeuralTextures	$57.82 \pm 3.29$	$0.609 \pm 0.038$	$0.573 \pm 0.046$	$0.580 \pm 0.029$	$0.566 \pm 0.063$	1.93
*DecisionTree*							
	Deepfakes	$63.54 \pm 1.43$	$0.635 \pm 0.014$	$0.636 \pm 0.010$	$0.635 \pm 0.018$	$0.636 \pm 0.008$	0.41
	Face2Face	$55.34 \pm 2.16$	$0.553 \pm 0.022$	$0.554 \pm 0.025$	$0.553 \pm 0.021$	$0.555 \pm 0.034$	0.40
	FaceShifter	$61.82 \pm 1.92$	$0.618 \pm 0.019$	$0.618 \pm 0.025$	$0.618 \pm 0.016$	$0.618 \pm 0.035$	0.40
	FaceSwap	$55.33 \pm 0.81$	$0.553 \pm 0.008$	$0.548 \pm 0.016$	$0.555 \pm 0.007$	$0.541 \pm 0.025$	0.39
	NeuralTextures	$52.75 \pm 2.58$	$0.528 \pm 0.026$	$0.526 \pm 0.023$	$0.527 \pm 0.027$	$0.524 \pm 0.038$	0.40
*ExtraTrees*							
	Deepfakes	$74.77 \pm 1.74$	$0.833 \pm 0.012$	$0.745 \pm 0.013$	$0.753 \pm 0.029$	$0.738 \pm 0.017$	0.86
	Face2Face	$65.69 \pm 2.03$	$0.712 \pm 0.015$	$0.648 \pm 0.022$	$0.665 \pm 0.020$	$0.631 \pm 0.025$	0.93
	FaceShifter	$74.42 \pm 1.36$	$0.823 \pm 0.008$	$0.738 \pm 0.017$	$0.757 \pm 0.010$	$0.719 \pm 0.026$	0.86
	FaceSwap	$65.39 \pm 0.71$	$0.719 \pm 0.010$	$0.648 \pm 0.005$	$0.660 \pm 0.010$	$0.636 \pm 0.002$	0.96
	NeuralTextures	$58.85 \pm 3.15$	$0.626 \pm 0.020$	$0.583 \pm 0.042$	$0.590 \pm 0.029$	$0.577 \pm 0.059$	0.99
*GradientBoosting*							
	Deepfakes	$72.76 \pm 2.42$	$0.807 \pm 0.034$	$0.727 \pm 0.027$	$0.728 \pm 0.022$	$0.727 \pm 0.039$	11.45
	Face2Face	$63.06 \pm 2.99$	$0.684 \pm 0.028$	$0.623 \pm 0.025$	$0.636 \pm 0.035$	$0.610 \pm 0.018$	10.73
	FaceShifter	$72.68 \pm 0.61$	$0.806 \pm 0.005$	$0.723 \pm 0.004$	$0.733 \pm 0.010$	$0.713 \pm 0.003$	10.99
	FaceSwap	$62.69 \pm 1.32$	$0.678 \pm 0.004$	$0.625 \pm 0.019$	$0.629 \pm 0.010$	$0.620 \pm 0.029$	10.26
	NeuralTextures	$58.18 \pm 0.24$	$0.624 \pm 0.030$	$0.577 \pm 0.013$	$0.583 \pm 0.005$	$0.572 \pm 0.030$	10.15
*KNN*							
	Deepfakes	$76.38 \pm 1.89$	$0.832 \pm 0.010$	$0.760 \pm 0.022$	$0.772 \pm 0.016$	$0.749 \pm 0.033$	0.00
	Face2Face	$58.86 \pm 0.77$	$0.624 \pm 0.012$	$0.559 \pm 0.017$	$0.602 \pm 0.005$	$0.522 \pm 0.027$	0.00
	FaceShifter	$71.64 \pm 2.51$	$0.778 \pm 0.019$	$0.701 \pm 0.026$	$0.741 \pm 0.030$	$0.665 \pm 0.027$	0.00
	FaceSwap	$62.16 \pm 5.85$	$0.659 \pm 0.070$	$0.617 \pm 0.050$	$0.626 \pm 0.067$	$0.608 \pm 0.048$	0.00
	NeuralTextures	$53.49 \pm 1.20$	$0.546 \pm 0.020$	$0.509 \pm 0.017$	$0.539 \pm 0.016$	$0.482 \pm 0.037$	0.00
*Logistic*							
	Deepfakes	$72.07 \pm 1.06$	$0.792 \pm 0.020$	$0.720 \pm 0.021$	$0.722 \pm 0.017$	$0.718 \pm 0.052$	0.64
	Face2Face	$62.94 \pm 1.37$	$0.679 \pm 0.024$	$0.621 \pm 0.009$	$0.636 \pm 0.021$	$0.607 \pm 0.018$	0.43
	FaceShifter	$72.00 \pm 1.23$	$0.799 \pm 0.011$	$0.715 \pm 0.010$	$0.728 \pm 0.024$	$0.703 \pm 0.023$	0.65
	FaceSwap	$61.28 \pm 0.57$	$0.654 \pm 0.007$	$0.612 \pm 0.006$	$0.614 \pm 0.012$	$0.610 \pm 0.024$	0.37
	NeuralTextures	$58.55 \pm 4.20$	$0.622 \pm 0.056$	$0.574 \pm 0.050$	$0.590 \pm 0.041$	$0.559 \pm 0.059$	0.36
*MLP*							
	Deepfakes	$73.71 \pm 1.20$	$0.813 \pm 0.024$	$0.737 \pm 0.012$	$0.737 \pm 0.015$	$0.737 \pm 0.017$	12.59
	Face2Face	$61.38 \pm 1.76$	$0.654 \pm 0.019$	$0.616 \pm 0.025$	$0.612 \pm 0.022$	$0.620 \pm 0.055$	17.15
	FaceShifter	$71.39 \pm 1.25$	$0.778 \pm 0.023$	$0.712 \pm 0.012$	$0.716 \pm 0.013$	$0.709 \pm 0.013$	15.07
	FaceSwap	$60.99 \pm 0.24$	$0.650 \pm 0.018$	$0.614 \pm 0.012$	$0.608 \pm 0.004$	$0.621 \pm 0.029$	20.05
	NeuralTextures	$55.12 \pm 1.94$	$0.565 \pm 0.031$	$0.554 \pm 0.037$	$0.550 \pm 0.017$	$0.558 \pm 0.065$	19.06
*RandomForest*							
	Deepfakes	$74.22 \pm 1.70$	$0.822 \pm 0.025$	$0.741 \pm 0.014$	$0.745 \pm 0.030$	$0.738 \pm 0.027$	3.88
	Face2Face	$64.39 \pm 2.61$	$0.703 \pm 0.029$	$0.633 \pm 0.028$	$0.653 \pm 0.027$	$0.615 \pm 0.029$	3.90
	FaceShifter	$73.29 \pm 1.74$	$0.812 \pm 0.011$	$0.725 \pm 0.021$	$0.746 \pm 0.013$	$0.706 \pm 0.030$	3.74
	FaceSwap	$64.24 \pm 1.43$	$0.700 \pm 0.006$	$0.637 \pm 0.016$	$0.647 \pm 0.014$	$0.628 \pm 0.019$	3.90
	NeuralTextures	$59.11 \pm 2.18$	$0.630 \pm 0.010$	$0.579 \pm 0.047$	$0.596 \pm 0.012$	$0.563 \pm 0.078$	3.88
*SVM-RBF*							
	Deepfakes	$76.69 \pm 1.51$	$0.841 \pm 0.018$	$0.768 \pm 0.016$	$0.765 \pm 0.019$	$0.771 \pm 0.031$	11.55
	Face2Face	$65.28 \pm 0.82$	$0.710 \pm 0.015$	$0.646 \pm 0.013$	$0.659 \pm 0.007$	$0.634 \pm 0.024$	14.14
	FaceShifter	$75.20 \pm 0.56$	$0.829 \pm 0.009$	$0.743 \pm 0.008$	$0.770 \pm 0.008$	$0.719 \pm 0.015$	11.78
	FaceSwap	$65.04 \pm 1.08$	$0.707 \pm 0.026$	$0.655 \pm 0.014$	$0.647 \pm 0.027$	$0.663 \pm 0.052$	14.08
	NeuralTextures	$58.74 \pm 2.70$	$0.628 \pm 0.033$	$0.577 \pm 0.043$	$0.591 \pm 0.022$	$0.563 \pm 0.062$	14.95
*XGBoost*							
	Deepfakes	$75.49 \pm 1.58$	$0.834 \pm 0.028$	$0.755 \pm 0.016$	$0.755 \pm 0.017$	$0.756 \pm 0.015$	0.26
	Face2Face	$63.41 \pm 2.38$	$0.683 \pm 0.024$	$0.633 \pm 0.028$	$0.635 \pm 0.022$	$0.630 \pm 0.037$	0.25
	FaceShifter	$74.08 \pm 1.16$	$0.817 \pm 0.010$	$0.735 \pm 0.016$	$0.751 \pm 0.012$	$0.721 \pm 0.030$	0.25
	FaceSwap	$63.18 \pm 2.24$	$0.685 \pm 0.025$	$0.631 \pm 0.025$	$0.633 \pm 0.021$	$0.628 \pm 0.028$	0.29
	NeuralTextures	$57.36 \pm 1.15$	$0.602 \pm 0.007$	$0.571 \pm 0.022$	$0.574 \pm 0.012$	$0.569 \pm 0.041$	0.34
**Average**		**65.19**	**0.702**	**0.647**	−	−	−

Method: kbest.

Values shown as Mean ± 95% Confidence Interval.

FaceForensics++ C40 compression level.

**Table 17:** Performance Results for kbest+lasso Feature Selection (FF++ c40 dataset).

**Classifier**	**Manipulation**	**Acc (%)**	**AUC**	**F1**	**Precision**	**Recall**	**Time (s)**
*AdaBoost*							
	Deepfakes	$69.69 \pm 2.76$	$0.774 \pm 0.026$	$0.691 \pm 0.028$	$0.704 \pm 0.030$	$0.678 \pm 0.028$	2.01
	Face2Face	$62.03 \pm 2.49$	$0.663 \pm 0.028$	$0.611 \pm 0.033$	$0.626 \pm 0.021$	$0.598 \pm 0.044$	1.68
	FaceShifter	$70.15 \pm 1.81$	$0.773 \pm 0.005$	$0.702 \pm 0.017$	$0.701 \pm 0.020$	$0.702 \pm 0.015$	1.89
	FaceSwap	$60.04 \pm 1.73$	$0.644 \pm 0.005$	$0.603 \pm 0.044$	$0.599 \pm 0.004$	$0.608 \pm 0.088$	1.44
	NeuralTextures	$58.11 \pm 3.63$	$0.613 \pm 0.044$	$0.575 \pm 0.047$	$0.583 \pm 0.033$	$0.568 \pm 0.060$	1.62
*DecisionTree*							
	Deepfakes	$63.14 \pm 1.97$	$0.631 \pm 0.020$	$0.631 \pm 0.008$	$0.632 \pm 0.029$	$0.630 \pm 0.017$	0.39
	Face2Face	$56.08 \pm 2.45$	$0.561 \pm 0.024$	$0.562 \pm 0.026$	$0.561 \pm 0.026$	$0.564 \pm 0.038$	0.33
	FaceShifter	$62.42 \pm 1.43$	$0.624 \pm 0.014$	$0.624 \pm 0.013$	$0.624 \pm 0.015$	$0.624 \pm 0.010$	0.36
	FaceSwap	$55.43 \pm 5.58$	$0.554 \pm 0.056$	$0.550 \pm 0.045$	$0.556 \pm 0.059$	$0.544 \pm 0.038$	0.28
	NeuralTextures	$53.05 \pm 0.96$	$0.531 \pm 0.010$	$0.534 \pm 0.024$	$0.530 \pm 0.009$	$0.538 \pm 0.044$	0.33
*ExtraTrees*							
	Deepfakes	$74.81 \pm 3.44$	$0.830 \pm 0.019$	$0.747 \pm 0.026$	$0.751 \pm 0.053$	$0.744 \pm 0.007$	0.80
	Face2Face	$64.82 \pm 0.70$	$0.708 \pm 0.006$	$0.639 \pm 0.014$	$0.656 \pm 0.002$	$0.623 \pm 0.025$	0.84
	FaceShifter	$74.70 \pm 1.03$	$0.823 \pm 0.014$	$0.740 \pm 0.012$	$0.760 \pm 0.013$	$0.721 \pm 0.019$	0.80
	FaceSwap	$64.41 \pm 0.34$	$0.710 \pm 0.002$	$0.637 \pm 0.006$	$0.651 \pm 0.002$	$0.623 \pm 0.010$	0.86
	NeuralTextures	$59.04 \pm 1.70$	$0.629 \pm 0.012$	$0.583 \pm 0.036$	$0.593 \pm 0.009$	$0.573 \pm 0.061$	0.91
*GradientBoosting*							
	Deepfakes	$72.91 \pm 1.61$	$0.807 \pm 0.029$	$0.730 \pm 0.015$	$0.727 \pm 0.022$	$0.734 \pm 0.023$	10.61
	Face2Face	$63.20 \pm 2.08$	$0.682 \pm 0.023$	$0.623 \pm 0.015$	$0.639 \pm 0.027$	$0.608 \pm 0.009$	8.87
	FaceShifter	$72.59 \pm 1.13$	$0.805 \pm 0.005$	$0.721 \pm 0.010$	$0.733 \pm 0.017$	$0.710 \pm 0.014$	9.93
	FaceSwap	$62.48 \pm 2.74$	$0.675 \pm 0.014$	$0.623 \pm 0.033$	$0.626 \pm 0.025$	$0.620 \pm 0.044$	7.51
	NeuralTextures	$58.40 \pm 2.93$	$0.622 \pm 0.029$	$0.581 \pm 0.053$	$0.584 \pm 0.020$	$0.578 \pm 0.085$	8.42
*KNN*							
	Deepfakes	$76.71 \pm 2.16$	$0.831 \pm 0.017$	$0.763 \pm 0.026$	$0.775 \pm 0.017$	$0.752 \pm 0.039$	0.00
	Face2Face	$59.76 \pm 1.62$	$0.635 \pm 0.017$	$0.572 \pm 0.035$	$0.610 \pm 0.010$	$0.539 \pm 0.056$	0.00
	FaceShifter	$71.76 \pm 2.20$	$0.777 \pm 0.024$	$0.702 \pm 0.028$	$0.742 \pm 0.019$	$0.667 \pm 0.038$	0.00
	FaceSwap	$61.62 \pm 0.46$	$0.653 \pm 0.021$	$0.609 \pm 0.024$	$0.621 \pm 0.011$	$0.598 \pm 0.056$	0.00
	NeuralTextures	$53.23 \pm 1.24$	$0.542 \pm 0.021$	$0.512 \pm 0.036$	$0.535 \pm 0.010$	$0.491 \pm 0.057$	0.00
*Logistic*							
	Deepfakes	$72.10 \pm 0.52$	$0.792 \pm 0.020$	$0.720 \pm 0.013$	$0.723 \pm 0.020$	$0.718 \pm 0.044$	0.60
	Face2Face	$63.12 \pm 1.66$	$0.679 \pm 0.023$	$0.623 \pm 0.011$	$0.638 \pm 0.023$	$0.608 \pm 0.014$	0.34
	FaceShifter	$71.99 \pm 1.37$	$0.800 \pm 0.011$	$0.715 \pm 0.008$	$0.728 \pm 0.026$	$0.703 \pm 0.016$	0.49
	FaceSwap	$61.19 \pm 0.42$	$0.654 \pm 0.008$	$0.611 \pm 0.008$	$0.613 \pm 0.009$	$0.609 \pm 0.023$	0.29
	NeuralTextures	$58.40 \pm 4.47$	$0.621 \pm 0.058$	$0.573 \pm 0.056$	$0.588 \pm 0.043$	$0.560 \pm 0.069$	0.43
*MLP*							
	Deepfakes	$74.09 \pm 3.67$	$0.812 \pm 0.040$	$0.739 \pm 0.047$	$0.744 \pm 0.026$	$0.735 \pm 0.077$	15.90
	Face2Face	$60.39 \pm 1.92$	$0.646 \pm 0.038$	$0.601 \pm 0.014$	$0.605 \pm 0.023$	$0.597 \pm 0.011$	18.33
	FaceShifter	$70.85 \pm 5.87$	$0.774 \pm 0.050$	$0.706 \pm 0.059$	$0.712 \pm 0.061$	$0.700 \pm 0.062$	19.11
	FaceSwap	$60.65 \pm 1.88$	$0.647 \pm 0.015$	$0.603 \pm 0.011$	$0.609 \pm 0.023$	$0.597 \pm 0.003$	22.44
	NeuralTextures	$55.22 \pm 2.98$	$0.568 \pm 0.042$	$0.555 \pm 0.042$	$0.551 \pm 0.027$	$0.559 \pm 0.059$	20.56
*RandomForest*							
	Deepfakes	$73.90 \pm 2.65$	$0.820 \pm 0.030$	$0.738 \pm 0.023$	$0.740 \pm 0.035$	$0.737 \pm 0.022$	3.40
	Face2Face	$64.32 \pm 1.89$	$0.701 \pm 0.020$	$0.633 \pm 0.026$	$0.652 \pm 0.016$	$0.616 \pm 0.038$	3.28
	FaceShifter	$73.40 \pm 0.46$	$0.814 \pm 0.004$	$0.727 \pm 0.002$	$0.746 \pm 0.013$	$0.710 \pm 0.013$	3.29
	FaceSwap	$63.85 \pm 0.94$	$0.693 \pm 0.000$	$0.635 \pm 0.012$	$0.641 \pm 0.012$	$0.629 \pm 0.023$	3.27
	NeuralTextures	$59.64 \pm 1.24$	$0.632 \pm 0.035$	$0.588 \pm 0.021$	$0.600 \pm 0.016$	$0.577 \pm 0.043$	3.45
*SVM-RBF*							
	Deepfakes	$76.58 \pm 1.33$	$0.838 \pm 0.016$	$0.767 \pm 0.013$	$0.763 \pm 0.022$	$0.772 \pm 0.029$	12.08
	Face2Face	$64.89 \pm 1.48$	$0.704 \pm 0.017$	$0.638 \pm 0.024$	$0.658 \pm 0.008$	$0.619 \pm 0.038$	15.15
	FaceShifter	$75.06 \pm 1.75$	$0.828 \pm 0.014$	$0.742 \pm 0.012$	$0.770 \pm 0.032$	$0.716 \pm 0.009$	12.66
	FaceSwap	$64.66 \pm 0.92$	$0.701 \pm 0.012$	$0.647 \pm 0.017$	$0.647 \pm 0.030$	$0.648 \pm 0.062$	13.65
	NeuralTextures	$58.91 \pm 4.09$	$0.624 \pm 0.043$	$0.580 \pm 0.053$	$0.592 \pm 0.039$	$0.567 \pm 0.067$	15.71
*XGBoost*							
	Deepfakes	$74.99 \pm 1.83$	$0.831 \pm 0.023$	$0.751 \pm 0.018$	$0.748 \pm 0.020$	$0.753 \pm 0.018$	0.23
	Face2Face	$61.59 \pm 2.72$	$0.671 \pm 0.019$	$0.615 \pm 0.019$	$0.616 \pm 0.034$	$0.614 \pm 0.016$	0.28
	FaceShifter	$73.49 \pm 0.55$	$0.814 \pm 0.019$	$0.730 \pm 0.001$	$0.744 \pm 0.016$	$0.717 \pm 0.014$	0.33
	FaceSwap	$63.06 \pm 1.77$	$0.679 \pm 0.003$	$0.628 \pm 0.020$	$0.633 \pm 0.018$	$0.623 \pm 0.024$	0.28
	NeuralTextures	$57.55 \pm 3.17$	$0.601 \pm 0.034$	$0.576 \pm 0.036$	$0.575 \pm 0.031$	$0.576 \pm 0.045$	0.25
**Average**		**65.09**	**0.701**	**0.646**	−	−	−

Method: kbest+lasso; Values shown as Mean ± 95% Confidence Interval; FaceForensics++ C40 compression level.

**Table 18:** Performance Results for rfe Feature Selection (FF++ c40 dataset).

**Classifier**	**Manipulation**	**Acc (%)**	**AUC**	**F1**	**Precision**	**Recall**	**Time (s)**
*AdaBoost*							
	Deepfakes	$70.38 \pm 0.21$	$0.774 \pm 0.012$	$0.708 \pm 0.009$	$0.698 \pm 0.016$	$0.718 \pm 0.035$	1.69
	Face2Face	$62.28 \pm 2.42$	$0.673 \pm 0.022$	$0.621 \pm 0.039$	$0.624 \pm 0.017$	$0.619 \pm 0.065$	1.62
	FaceShifter	$69.24 \pm 4.32$	$0.760 \pm 0.031$	$0.692 \pm 0.046$	$0.692 \pm 0.042$	$0.692 \pm 0.058$	1.70
	FaceSwap	$62.25 \pm 3.04$	$0.669 \pm 0.034$	$0.617 \pm 0.035$	$0.626 \pm 0.028$	$0.609 \pm 0.041$	1.65
	NeuralTextures	$58.50 \pm 1.92$	$0.621 \pm 0.012$	$0.580 \pm 0.039$	$0.586 \pm 0.011$	$0.574 \pm 0.067$	1.38
*DecisionTree*							
	Deepfakes	$60.92 \pm 3.48$	$0.609 \pm 0.035$	$0.608 \pm 0.033$	$0.610 \pm 0.037$	$0.607 \pm 0.036$	0.32
	Face2Face	$55.82 \pm 1.90$	$0.558 \pm 0.019$	$0.557 \pm 0.028$	$0.558 \pm 0.018$	$0.557 \pm 0.043$	0.32
	FaceShifter	$59.75 \pm 7.01$	$0.598 \pm 0.070$	$0.599 \pm 0.073$	$0.596 \pm 0.068$	$0.602 \pm 0.077$	0.32
	FaceSwap	$56.31 \pm 1.90$	$0.563 \pm 0.019$	$0.561 \pm 0.027$	$0.564 \pm 0.019$	$0.558 \pm 0.042$	0.32
	NeuralTextures	$53.22 \pm 4.78$	$0.532 \pm 0.048$	$0.534 \pm 0.066$	$0.531 \pm 0.045$	$0.537 \pm 0.087$	0.28
*ExtraTrees*							
	Deepfakes	$74.48 \pm 1.04$	$0.822 \pm 0.020$	$0.742 \pm 0.013$	$0.750 \pm 0.006$	$0.734 \pm 0.019$	0.87
	Face2Face	$64.94 \pm 1.92$	$0.710 \pm 0.027$	$0.646 \pm 0.017$	$0.652 \pm 0.024$	$0.640 \pm 0.022$	0.94
	FaceShifter	$72.76 \pm 4.46$	$0.803 \pm 0.053$	$0.723 \pm 0.049$	$0.736 \pm 0.040$	$0.710 \pm 0.059$	0.88
	FaceSwap	$66.37 \pm 2.00$	$0.725 \pm 0.009$	$0.658 \pm 0.017$	$0.670 \pm 0.031$	$0.645 \pm 0.031$	0.93
	NeuralTextures	$59.35 \pm 1.01$	$0.634 \pm 0.017$	$0.588 \pm 0.003$	$0.596 \pm 0.015$	$0.580 \pm 0.015$	0.99
*GradientBoosting*							
	Deepfakes	$73.22 \pm 1.57$	$0.810 \pm 0.013$	$0.735 \pm 0.016$	$0.727 \pm 0.014$	$0.743 \pm 0.019$	8.86
	Face2Face	$64.50 \pm 2.22$	$0.697 \pm 0.026$	$0.642 \pm 0.031$	$0.648 \pm 0.016$	$0.636 \pm 0.045$	8.54
	FaceShifter	$72.41 \pm 4.62$	$0.794 \pm 0.047$	$0.721 \pm 0.047$	$0.730 \pm 0.048$	$0.712 \pm 0.049$	8.88
	FaceSwap	$65.47 \pm 1.88$	$0.709 \pm 0.030$	$0.657 \pm 0.015$	$0.653 \pm 0.022$	$0.661 \pm 0.013$	8.66
	NeuralTextures	$59.33 \pm 1.60$	$0.627 \pm 0.009$	$0.589 \pm 0.029$	$0.595 \pm 0.012$	$0.583 \pm 0.048$	7.25
*KNN*							
	Deepfakes	$69.53 \pm 0.76$	$0.753 \pm 0.007$	$0.703 \pm 0.008$	$0.686 \pm 0.018$	$0.720 \pm 0.031$	0.00
	Face2Face	$58.38 \pm 2.62$	$0.617 \pm 0.022$	$0.571 \pm 0.027$	$0.589 \pm 0.028$	$0.553 \pm 0.027$	0.00
	FaceShifter	$66.78 \pm 3.95$	$0.717 \pm 0.053$	$0.652 \pm 0.037$	$0.684 \pm 0.048$	$0.624 \pm 0.031$	0.00
	FaceSwap	$61.40 \pm 1.97$	$0.651 \pm 0.020$	$0.609 \pm 0.011$	$0.618 \pm 0.031$	$0.601 \pm 0.033$	0.00
	NeuralTextures	$54.98 \pm 2.49$	$0.565 \pm 0.023$	$0.535 \pm 0.027$	$0.553 \pm 0.026$	$0.518 \pm 0.029$	0.00
*Logistic*							
	Deepfakes	$72.36 \pm 1.78$	$0.799 \pm 0.016$	$0.725 \pm 0.019$	$0.721 \pm 0.016$	$0.728 \pm 0.024$	0.44
	Face2Face	$64.47 \pm 2.41$	$0.696 \pm 0.041$	$0.643 \pm 0.023$	$0.646 \pm 0.027$	$0.640 \pm 0.029$	0.42
	FaceShifter	$72.20 \pm 6.47$	$0.793 \pm 0.074$	$0.719 \pm 0.063$	$0.727 \pm 0.071$	$0.712 \pm 0.057$	0.28
	FaceSwap	$63.85 \pm 2.09$	$0.691 \pm 0.020$	$0.642 \pm 0.021$	$0.636 \pm 0.021$	$0.648 \pm 0.026$	0.37
	NeuralTextures	$59.15 \pm 1.28$	$0.627 \pm 0.013$	$0.581 \pm 0.018$	$0.596 \pm 0.024$	$0.568 \pm 0.052$	0.36
*MLP*							
	Deepfakes	$70.57 \pm 4.62$	$0.771 \pm 0.046$	$0.706 \pm 0.047$	$0.705 \pm 0.044$	$0.708 \pm 0.051$	14.61
	Face2Face	$61.86 \pm 3.15$	$0.655 \pm 0.030$	$0.620 \pm 0.025$	$0.618 \pm 0.037$	$0.622 \pm 0.027$	17.70
	FaceShifter	$69.85 \pm 5.52$	$0.755 \pm 0.065$	$0.701 \pm 0.054$	$0.696 \pm 0.055$	$0.706 \pm 0.054$	15.91
	FaceSwap	$62.11 \pm 2.16$	$0.663 \pm 0.024$	$0.618 \pm 0.038$	$0.623 \pm 0.012$	$0.614 \pm 0.065$	20.00
	NeuralTextures	$56.26 \pm 1.55$	$0.584 \pm 0.014$	$0.557 \pm 0.021$	$0.564 \pm 0.019$	$0.550 \pm 0.045$	19.16
*RandomForest*							
	Deepfakes	$73.49 \pm 0.80$	$0.812 \pm 0.018$	$0.734 \pm 0.005$	$0.737 \pm 0.014$	$0.731 \pm 0.005$	3.13
	Face2Face	$64.57 \pm 1.79$	$0.700 \pm 0.018$	$0.639 \pm 0.014$	$0.651 \pm 0.022$	$0.627 \pm 0.011$	3.20
	FaceShifter	$72.32 \pm 4.80$	$0.795 \pm 0.056$	$0.716 \pm 0.049$	$0.734 \pm 0.051$	$0.699 \pm 0.047$	3.13
	FaceSwap	$66.23 \pm 1.96$	$0.721 \pm 0.031$	$0.659 \pm 0.016$	$0.666 \pm 0.024$	$0.652 \pm 0.008$	3.26
	NeuralTextures	$58.74 \pm 0.63$	$0.628 \pm 0.009$	$0.577 \pm 0.031$	$0.591 \pm 0.007$	$0.563 \pm 0.064$	2.95
*SVM-RBF*							
	Deepfakes	$74.42 \pm 2.96$	$0.816 \pm 0.012$	$0.745 \pm 0.031$	$0.743 \pm 0.026$	$0.747 \pm 0.036$	11.55
	Face2Face	$65.33 \pm 2.73$	$0.710 \pm 0.023$	$0.653 \pm 0.028$	$0.654 \pm 0.027$	$0.653 \pm 0.031$	13.21
	FaceShifter	$73.10 \pm 7.40$	$0.804 \pm 0.072$	$0.729 \pm 0.070$	$0.735 \pm 0.084$	$0.723 \pm 0.059$	11.84
	FaceSwap	$66.10 \pm 3.06$	$0.721 \pm 0.021$	$0.665 \pm 0.025$	$0.658 \pm 0.042$	$0.672 \pm 0.047$	13.32
	NeuralTextures	$58.84 \pm 1.20$	$0.629 \pm 0.005$	$0.578 \pm 0.005$	$0.592 \pm 0.018$	$0.565 \pm 0.017$	14.46
*XGBoost*							
	Deepfakes	$73.82 \pm 1.69$	$0.819 \pm 0.028$	$0.740 \pm 0.022$	$0.736 \pm 0.009$	$0.744 \pm 0.038$	0.36
	Face2Face	$63.47 \pm 1.50$	$0.690 \pm 0.020$	$0.635 \pm 0.028$	$0.634 \pm 0.006$	$0.635 \pm 0.050$	0.27
	FaceShifter	$72.67 \pm 6.04$	$0.800 \pm 0.075$	$0.725 \pm 0.058$	$0.729 \pm 0.065$	$0.722 \pm 0.054$	0.35
	FaceSwap	$64.91 \pm 1.41$	$0.706 \pm 0.028$	$0.649 \pm 0.009$	$0.650 \pm 0.021$	$0.648 \pm 0.017$	0.27
	NeuralTextures	$58.02 \pm 3.33$	$0.615 \pm 0.038$	$0.581 \pm 0.028$	$0.580 \pm 0.038$	$0.583 \pm 0.046$	0.34
**Average**		**65.03**	**0.700**	**0.648**	−	−	−

Method: rfe; Values shown as Mean ± 95% Confidence Interval; FaceForensics++ C40 compression level.

**Table 19:** Performance Results for rfe+lasso Feature Selection (FF++ c40 dataset).

**Classifier**	**Manipulation**	**Acc (%)**	**AUC**	**F1**	**Precision**	**Recall**	**Time (s)**
*AdaBoost*							
	Deepfakes	$70.38 \pm 0.21$	$0.774 \pm 0.012$	$0.708 \pm 0.009$	$0.698 \pm 0.016$	$0.718 \pm 0.035$	1.69
	Face2Face	$62.28 \pm 2.42$	$0.673 \pm 0.022$	$0.621 \pm 0.039$	$0.624 \pm 0.017$	$0.619 \pm 0.065$	1.62
	FaceShifter	$69.24 \pm 4.32$	$0.760 \pm 0.031$	$0.692 \pm 0.046$	$0.692 \pm 0.042$	$0.692 \pm 0.058$	1.70
	FaceSwap	$62.25 \pm 3.04$	$0.669 \pm 0.034$	$0.617 \pm 0.035$	$0.626 \pm 0.028$	$0.609 \pm 0.041$	1.63
	NeuralTextures	$58.50 \pm 1.92$	$0.621 \pm 0.012$	$0.580 \pm 0.039$	$0.586 \pm 0.011$	$0.574 \pm 0.067$	1.38
*DecisionTree*							
	Deepfakes	$60.92 \pm 3.48$	$0.609 \pm 0.035$	$0.608 \pm 0.033$	$0.610 \pm 0.037$	$0.607 \pm 0.036$	0.31
	Face2Face	$55.82 \pm 1.90$	$0.558 \pm 0.019$	$0.557 \pm 0.028$	$0.558 \pm 0.018$	$0.557 \pm 0.043$	0.32
	FaceShifter	$59.75 \pm 7.01$	$0.598 \pm 0.070$	$0.599 \pm 0.073$	$0.596 \pm 0.068$	$0.602 \pm 0.077$	0.32
	FaceSwap	$56.31 \pm 1.90$	$0.563 \pm 0.019$	$0.561 \pm 0.027$	$0.564 \pm 0.019$	$0.558 \pm 0.042$	0.32
	NeuralTextures	$53.22 \pm 4.78$	$0.532 \pm 0.048$	$0.534 \pm 0.066$	$0.531 \pm 0.045$	$0.537 \pm 0.087$	0.28
*ExtraTrees*							
	Deepfakes	$74.48 \pm 1.04$	$0.822 \pm 0.020$	$0.742 \pm 0.013$	$0.750 \pm 0.006$	$0.734 \pm 0.019$	0.87
	Face2Face	$64.94 \pm 1.92$	$0.710 \pm 0.027$	$0.646 \pm 0.017$	$0.652 \pm 0.024$	$0.640 \pm 0.022$	0.94
	FaceShifter	$72.76 \pm 4.46$	$0.803 \pm 0.053$	$0.723 \pm 0.049$	$0.736 \pm 0.040$	$0.710 \pm 0.059$	0.88
	FaceSwap	$66.37 \pm 2.00$	$0.725 \pm 0.009$	$0.658 \pm 0.017$	$0.670 \pm 0.031$	$0.645 \pm 0.031$	0.94
	NeuralTextures	$59.35 \pm 1.01$	$0.634 \pm 0.017$	$0.588 \pm 0.003$	$0.596 \pm 0.015$	$0.580 \pm 0.015$	0.99
*GradientBoosting*							
	Deepfakes	$73.22 \pm 1.57$	$0.810 \pm 0.013$	$0.735 \pm 0.016$	$0.727 \pm 0.014$	$0.743 \pm 0.019$	8.88
	Face2Face	$64.50 \pm 2.22$	$0.697 \pm 0.026$	$0.642 \pm 0.031$	$0.648 \pm 0.016$	$0.636 \pm 0.045$	8.55
	FaceShifter	$72.41 \pm 4.62$	$0.794 \pm 0.047$	$0.721 \pm 0.047$	$0.730 \pm 0.048$	$0.712 \pm 0.049$	8.88
	FaceSwap	$65.47 \pm 1.88$	$0.709 \pm 0.030$	$0.657 \pm 0.015$	$0.653 \pm 0.022$	$0.661 \pm 0.013$	8.67
	NeuralTextures	$59.33 \pm 1.60$	$0.627 \pm 0.009$	$0.589 \pm 0.029$	$0.595 \pm 0.012$	$0.583 \pm 0.048$	7.34
*KNN*							
	Deepfakes	$69.53 \pm 0.76$	$0.753 \pm 0.007$	$0.703 \pm 0.008$	$0.686 \pm 0.018$	$0.720 \pm 0.031$	0.00
	Face2Face	$58.38 \pm 2.62$	$0.617 \pm 0.022$	$0.571 \pm 0.027$	$0.589 \pm 0.028$	$0.553 \pm 0.027$	0.00
	FaceShifter	$66.78 \pm 3.95$	$0.717 \pm 0.053$	$0.652 \pm 0.037$	$0.684 \pm 0.048$	$0.624 \pm 0.031$	0.00
	FaceSwap	$61.40 \pm 1.97$	$0.651 \pm 0.020$	$0.609 \pm 0.011$	$0.618 \pm 0.031$	$0.601 \pm 0.033$	0.00
	NeuralTextures	$54.98 \pm 2.49$	$0.565 \pm 0.023$	$0.535 \pm 0.027$	$0.553 \pm 0.026$	$0.518 \pm 0.029$	0.00
*Logistic*							
	Deepfakes	$72.36 \pm 1.78$	$0.799 \pm 0.016$	$0.725 \pm 0.019$	$0.721 \pm 0.016$	$0.728 \pm 0.024$	0.27
	Face2Face	$64.47 \pm 2.41$	$0.696 \pm 0.041$	$0.643 \pm 0.023$	$0.646 \pm 0.027$	$0.640 \pm 0.029$	0.50
	FaceShifter	$72.20 \pm 6.47$	$0.793 \pm 0.074$	$0.719 \pm 0.063$	$0.727 \pm 0.071$	$0.712 \pm 0.057$	0.34
	FaceSwap	$63.85 \pm 2.09$	$0.691 \pm 0.020$	$0.642 \pm 0.021$	$0.636 \pm 0.021$	$0.648 \pm 0.026$	0.42
	NeuralTextures	$59.15 \pm 1.28$	$0.627 \pm 0.013$	$0.581 \pm 0.018$	$0.596 \pm 0.024$	$0.568 \pm 0.052$	0.52
*MLP*							
	Deepfakes	$70.39 \pm 3.89$	$0.769 \pm 0.045$	$0.705 \pm 0.044$	$0.702 \pm 0.033$	$0.709 \pm 0.057$	15.02
	Face2Face	$62.02 \pm 2.38$	$0.659 \pm 0.030$	$0.623 \pm 0.020$	$0.619 \pm 0.028$	$0.627 \pm 0.026$	17.17
	FaceShifter	$69.40 \pm 7.14$	$0.752 \pm 0.073$	$0.695 \pm 0.069$	$0.693 \pm 0.075$	$0.698 \pm 0.064$	17.26
	FaceSwap	$62.08 \pm 3.04$	$0.661 \pm 0.016$	$0.619 \pm 0.044$	$0.622 \pm 0.022$	$0.615 \pm 0.066$	20.09
	NeuralTextures	$56.16 \pm 0.72$	$0.585 \pm 0.021$	$0.556 \pm 0.029$	$0.562 \pm 0.009$	$0.551 \pm 0.061$	18.60
*RandomForest*							
	Deepfakes	$73.49 \pm 0.80$	$0.812 \pm 0.018$	$0.734 \pm 0.005$	$0.737 \pm 0.014$	$0.731 \pm 0.005$	3.15
	Face2Face	$64.57 \pm 1.79$	$0.700 \pm 0.018$	$0.639 \pm 0.014$	$0.651 \pm 0.022$	$0.627 \pm 0.011$	3.22
	FaceShifter	$72.32 \pm 4.80$	$0.795 \pm 0.056$	$0.716 \pm 0.049$	$0.734 \pm 0.051$	$0.699 \pm 0.047$	3.14
	FaceSwap	$66.23 \pm 1.96$	$0.721 \pm 0.031$	$0.659 \pm 0.016$	$0.666 \pm 0.024$	$0.652 \pm 0.008$	3.26
	NeuralTextures	$58.74 \pm 0.63$	$0.628 \pm 0.009$	$0.577 \pm 0.031$	$0.591 \pm 0.007$	$0.563 \pm 0.064$	2.99
*SVM-RBF*							
	Deepfakes	$74.42 \pm 2.96$	$0.816 \pm 0.012$	$0.745 \pm 0.031$	$0.743 \pm 0.026$	$0.747 \pm 0.036$	11.57
	Face2Face	$65.33 \pm 2.73$	$0.710 \pm 0.023$	$0.653 \pm 0.028$	$0.654 \pm 0.027$	$0.653 \pm 0.031$	13.34
	FaceShifter	$73.10 \pm 7.40$	$0.804 \pm 0.072$	$0.729 \pm 0.070$	$0.735 \pm 0.084$	$0.723 \pm 0.059$	11.34
	FaceSwap	$66.10 \pm 3.06$	$0.721 \pm 0.021$	$0.665 \pm 0.025$	$0.658 \pm 0.042$	$0.672 \pm 0.047$	13.28
	NeuralTextures	$58.84 \pm 1.20$	$0.629 \pm 0.005$	$0.578 \pm 0.005$	$0.592 \pm 0.018$	$0.565 \pm 0.017$	14.06
*XGBoost*							
	Deepfakes	$73.82 \pm 1.69$	$0.819 \pm 0.028$	$0.740 \pm 0.022$	$0.736 \pm 0.009$	$0.744 \pm 0.038$	0.37
	Face2Face	$63.47 \pm 1.50$	$0.690 \pm 0.020$	$0.635 \pm 0.028$	$0.634 \pm 0.006$	$0.635 \pm 0.050$	0.35
	FaceShifter	$72.67 \pm 6.04$	$0.800 \pm 0.075$	$0.725 \pm 0.058$	$0.729 \pm 0.065$	$0.722 \pm 0.054$	0.31
	FaceSwap	$64.91 \pm 1.41$	$0.706 \pm 0.028$	$0.649 \pm 0.009$	$0.650 \pm 0.021$	$0.648 \pm 0.017$	0.36
	NeuralTextures	$58.02 \pm 3.33$	$0.615 \pm 0.038$	$0.581 \pm 0.028$	$0.580 \pm 0.038$	$0.583 \pm 0.046$	0.57
**Average**		**65.01**	**0.700**	**0.648**	−	−	−

Method: rfe+lasso; Values shown as Mean ± 95% Confidence Interval; FaceForensics++ C40 compression level.

**Table 20:** Performance Results for ga Feature Selection (FF++ C23 dataset).

**Classifier**	**Manipulation**	**Acc (%)**	**AUC**	**F1**	**Precision**	**Recall**	**Time (s)**
*AdaBoost*							
	DeepFakes	$78.05 \pm 0.98$	$0.864 \pm 0.009$	$0.781 \pm 0.004$	$0.780 \pm 0.029$	$0.781 \pm 0.026$	92.01
	Face2Face	$78.05 \pm 0.98$	$0.864 \pm 0.009$	$0.781 \pm 0.004$	$0.780 \pm 0.029$	$0.781 \pm 0.026$	92.01
	FaceShifter	$77.27 \pm 1.52$	$0.853 \pm 0.018$	$0.768 \pm 0.022$	$0.784 \pm 0.013$	$0.753 \pm 0.044$	112.46
	FaceSwap	$74.12 \pm 3.51$	$0.819 \pm 0.014$	$0.775 \pm 0.033$	$0.753 \pm 0.026$	$0.798 \pm 0.041$	33.73
	NeuralTextures	$72.09 \pm 1.26$	$0.789 \pm 0.013$	$0.756 \pm 0.006$	$0.737 \pm 0.029$	$0.777 \pm 0.034$	104.91
*DecisionTree*							
	DeepFakes	$74.21 \pm 1.59$	$0.742 \pm 0.016$	$0.742 \pm 0.016$	$0.743 \pm 0.016$	$0.740 \pm 0.017$	25.46
	Face2Face	$74.21 \pm 1.59$	$0.742 \pm 0.016$	$0.742 \pm 0.016$	$0.743 \pm 0.016$	$0.740 \pm 0.017$	25.46
	FaceShifter	$72.41 \pm 1.02$	$0.724 \pm 0.010$	$0.724 \pm 0.005$	$0.725 \pm 0.019$	$0.722 \pm 0.011$	33.78
	FaceSwap	$64.82 \pm 1.98$	$0.643 \pm 0.021$	$0.685 \pm 0.015$	$0.685 \pm 0.021$	$0.685 \pm 0.010$	7.28
	NeuralTextures	$65.77 \pm 1.18$	$0.654 \pm 0.011$	$0.692 \pm 0.013$	$0.694 \pm 0.008$	$0.689 \pm 0.020$	29.37
*ExtraTrees*							
	DeepFakes	$92.67 \pm 0.38$	$0.980 \pm 0.002$	$0.926 \pm 0.004$	$0.939 \pm 0.009$	$0.913 \pm 0.011$	7.22
	Face2Face	$92.67 \pm 0.38$	$0.980 \pm 0.002$	$0.926 \pm 0.004$	$0.939 \pm 0.009$	$0.913 \pm 0.011$	7.22
	FaceShifter	$88.56 \pm 1.04$	$0.954 \pm 0.006$	$0.885 \pm 0.011$	$0.891 \pm 0.007$	$0.879 \pm 0.016$	12.06
	FaceSwap	$80.73 \pm 1.39$	$0.897 \pm 0.011$	$0.838 \pm 0.008$	$0.789 \pm 0.024$	$0.894 \pm 0.014$	3.01
	NeuralTextures	$75.43 \pm 0.76$	$0.836 \pm 0.009$	$0.797 \pm 0.005$	$0.738 \pm 0.010$	$0.866 \pm 0.008$	11.76
*GradientBoosting*							
	DeepFakes	$85.45 \pm 1.08$	$0.933 \pm 0.005$	$0.854 \pm 0.010$	$0.859 \pm 0.015$	$0.848 \pm 0.007$	501.12
	Face2Face	$85.45 \pm 1.08$	$0.933 \pm 0.005$	$0.854 \pm 0.010$	$0.859 \pm 0.015$	$0.848 \pm 0.007$	501.12
	FaceShifter	$84.18 \pm 1.24$	$0.919 \pm 0.011$	$0.838 \pm 0.012$	$0.859 \pm 0.015$	$0.818 \pm 0.011$	607.99
	FaceSwap	$79.80 \pm 2.96$	$0.881 \pm 0.025$	$0.825 \pm 0.026$	$0.800 \pm 0.026$	$0.851 \pm 0.034$	180.94
	NeuralTextures	$75.75 \pm 0.59$	$0.837 \pm 0.009$	$0.790 \pm 0.006$	$0.763 \pm 0.005$	$0.819 \pm 0.014$	567.53
*KNN*							
	DeepFakes	$94.45 \pm 0.65$	$0.987 \pm 0.003$	$0.944 \pm 0.006$	$0.946 \pm 0.008$	$0.943 \pm 0.005$	0.03
	Face2Face	$94.45 \pm 0.65$	$0.987 \pm 0.003$	$0.944 \pm 0.006$	$0.946 \pm 0.008$	$0.943 \pm 0.005$	0.03
	FaceShifter	$91.16 \pm 0.76$	$0.971 \pm 0.005$	$0.908 \pm 0.008$	$0.946 \pm 0.009$	$0.874 \pm 0.012$	0.05
	FaceSwap	$75.03 \pm 1.71$	$0.824 \pm 0.015$	$0.785 \pm 0.011$	$0.756 \pm 0.022$	$0.817 \pm 0.003$	0.01
	NeuralTextures	$62.58 \pm 0.66$	$0.686 \pm 0.001$	$0.631 \pm 0.005$	$0.700 \pm 0.009$	$0.574 \pm 0.003$	0.05
*Logistic*							
	DeepFakes	$90.12 \pm 0.97$	$0.965 \pm 0.005$	$0.902 \pm 0.009$	$0.899 \pm 0.017$	$0.904 \pm 0.012$	1.87
	Face2Face	$90.12 \pm 0.97$	$0.965 \pm 0.005$	$0.902 \pm 0.009$	$0.899 \pm 0.017$	$0.904 \pm 0.012$	1.87
	FaceShifter	$89.77 \pm 1.03$	$0.958 \pm 0.003$	$0.897 \pm 0.010$	$0.901 \pm 0.010$	$0.894 \pm 0.011$	5.89
	FaceSwap	$83.44 \pm 2.11$	$0.910 \pm 0.019$	$0.852 \pm 0.018$	$0.852 \pm 0.031$	$0.851 \pm 0.030$	4.59
	NeuralTextures	$81.81 \pm 1.10$	$0.893 \pm 0.006$	$0.838 \pm 0.011$	$0.829 \pm 0.004$	$0.848 \pm 0.019$	4.95
*MLP*							
	DeepFakes	$96.82 \pm 0.42$	$0.993 \pm 0.001$	$0.968 \pm 0.004$	$0.971 \pm 0.011$	$0.965 \pm 0.003$	11.35
	Face2Face	$96.82 \pm 0.42$	$0.993 \pm 0.001$	$0.968 \pm 0.004$	$0.971 \pm 0.011$	$0.965 \pm 0.003$	11.35
	FaceShifter	$95.58 \pm 0.81$	$0.989 \pm 0.000$	$0.956 \pm 0.008$	$0.958 \pm 0.004$	$0.953 \pm 0.015$	17.99
	FaceSwap	$89.29 \pm 1.74$	$0.954 \pm 0.012$	$0.905 \pm 0.017$	$0.900 \pm 0.010$	$0.909 \pm 0.032$	5.35
	NeuralTextures	$88.49 \pm 1.27$	$0.950 \pm 0.004$	$0.897 \pm 0.011$	$0.895 \pm 0.018$	$0.898 \pm 0.016$	19.23
*RandomForest*							
	DeepFakes	$91.11 \pm 0.35$	$0.972 \pm 0.002$	$0.910 \pm 0.003$	$0.919 \pm 0.012$	$0.901 \pm 0.007$	42.09
	Face2Face	$91.11 \pm 0.35$	$0.972 \pm 0.002$	$0.910 \pm 0.003$	$0.919 \pm 0.012$	$0.901 \pm 0.007$	42.09
	FaceShifter	$87.35 \pm 0.61$	$0.946 \pm 0.006$	$0.872 \pm 0.007$	$0.884 \pm 0.001$	$0.860 \pm 0.014$	55.42
	FaceSwap	$79.82 \pm 2.68$	$0.884 \pm 0.014$	$0.830 \pm 0.017$	$0.782 \pm 0.039$	$0.885 \pm 0.018$	14.21
	NeuralTextures	$75.81 \pm 1.44$	$0.842 \pm 0.016$	$0.799 \pm 0.011$	$0.743 \pm 0.015$	$0.865 \pm 0.012$	50.39
*SVM-RBF*							
	DeepFakes	$95.75 \pm 0.48$	$0.992 \pm 0.001$	$0.957 \pm 0.005$	$0.964 \pm 0.008$	$0.951 \pm 0.003$	664.90
	Face2Face	$95.75 \pm 0.48$	$0.992 \pm 0.001$	$0.957 \pm 0.005$	$0.964 \pm 0.008$	$0.951 \pm 0.003$	664.90
	FaceShifter	$94.84 \pm 0.92$	$0.985 \pm 0.004$	$0.948 \pm 0.010$	$0.949 \pm 0.000$	$0.948 \pm 0.020$	1171.27
	FaceSwap	$87.15 \pm 1.13$	$0.946 \pm 0.011$	$0.889 \pm 0.009$	$0.862 \pm 0.019$	$0.917 \pm 0.019$	89.01
	NeuralTextures	$85.63 \pm 0.71$	$0.930 \pm 0.004$	$0.874 \pm 0.007$	$0.855 \pm 0.001$	$0.894 \pm 0.014$	968.27
*XGBoost*							
	DeepFakes	$94.16 \pm 0.71$	$0.986 \pm 0.002$	$0.941 \pm 0.007$	$0.946 \pm 0.009$	$0.937 \pm 0.005$	26.86
	Face2Face	$94.16 \pm 0.71$	$0.986 \pm 0.002$	$0.941 \pm 0.007$	$0.946 \pm 0.009$	$0.937 \pm 0.005$	26.86
	FaceShifter	$92.11 \pm 0.88$	$0.977 \pm 0.006$	$0.921 \pm 0.010$	$0.926 \pm 0.005$	$0.915 \pm 0.018$	6.34
	FaceSwap	$84.84 \pm 2.81$	$0.929 \pm 0.010$	$0.867 \pm 0.025$	$0.852 \pm 0.030$	$0.881 \pm 0.030$	4.37
	NeuralTextures	$82.44 \pm 0.76$	$0.904 \pm 0.009$	$0.845 \pm 0.006$	$0.829 \pm 0.010$	$0.862 \pm 0.010$	6.19
**Average**		**84.47**	**0.902**	**0.855**	−	−	−

Method: ga; Values shown as Mean ± 95% Confidence Interval; FaceForensics++ C23 compression level.

**Table 21:** Performance Results for ga+lasso Feature Selection (FF++ c23 dataset).

**Classifier**	**Manipulation**	**Acc (%)**	**AUC**	**F1**	**Precision**	**Recall**	**Time (s)**
*AdaBoost*							
	DeepFakes	$78.45 \pm 2.55$	$0.867 \pm 0.022$	$0.782 \pm 0.024$	$0.790 \pm 0.033$	$0.775 \pm 0.023$	66.51
	Face2Face	$78.45 \pm 2.55$	$0.867 \pm 0.022$	$0.782 \pm 0.024$	$0.790 \pm 0.033$	$0.775 \pm 0.023$	66.51
	FaceShifter	$77.00 \pm 0.64$	$0.851 \pm 0.015$	$0.767 \pm 0.009$	$0.777 \pm 0.028$	$0.759 \pm 0.040$	82.20
	FaceSwap	$74.58 \pm 3.37$	$0.823 \pm 0.033$	$0.775 \pm 0.034$	$0.766 \pm 0.026$	$0.785 \pm 0.053$	21.08
	NeuralTextures	$72.02 \pm 1.09$	$0.790 \pm 0.009$	$0.755 \pm 0.004$	$0.738 \pm 0.032$	$0.773 \pm 0.041$	76.99
*DecisionTree*							
	DeepFakes	$73.88 \pm 1.85$	$0.739 \pm 0.019$	$0.739 \pm 0.017$	$0.738 \pm 0.021$	$0.740 \pm 0.013$	17.81
	Face2Face	$73.88 \pm 1.85$	$0.739 \pm 0.019$	$0.739 \pm 0.017$	$0.738 \pm 0.021$	$0.740 \pm 0.013$	17.81
	FaceShifter	$72.58 \pm 2.64$	$0.726 \pm 0.026$	$0.724 \pm 0.028$	$0.729 \pm 0.025$	$0.720 \pm 0.031$	25.32
	FaceSwap	$65.48 \pm 1.98$	$0.651 \pm 0.022$	$0.690 \pm 0.014$	$0.692 \pm 0.024$	$0.688 \pm 0.010$	4.80
	NeuralTextures	$65.46 \pm 0.69$	$0.650 \pm 0.006$	$0.690 \pm 0.010$	$0.690 \pm 0.002$	$0.690 \pm 0.018$	21.47
*ExtraTrees*							
	DeepFakes	$92.81 \pm 0.95$	$0.980 \pm 0.001$	$0.927 \pm 0.010$	$0.941 \pm 0.010$	$0.914 \pm 0.009$	6.21
	Face2Face	$92.81 \pm 0.95$	$0.980 \pm 0.001$	$0.927 \pm 0.010$	$0.941 \pm 0.010$	$0.914 \pm 0.009$	6.21
	FaceShifter	$88.39 \pm 0.71$	$0.953 \pm 0.006$	$0.883 \pm 0.007$	$0.889 \pm 0.009$	$0.877 \pm 0.012$	10.06
	FaceSwap	$81.50 \pm 1.81$	$0.898 \pm 0.004$	$0.844 \pm 0.018$	$0.797 \pm 0.009$	$0.898 \pm 0.037$	2.23
	NeuralTextures	$75.53 \pm 0.65$	$0.836 \pm 0.005$	$0.797 \pm 0.003$	$0.740 \pm 0.014$	$0.863 \pm 0.019$	10.08
*GradientBoosting*							
	DeepFakes	$85.11 \pm 1.02$	$0.932 \pm 0.006$	$0.851 \pm 0.010$	$0.853 \pm 0.015$	$0.848 \pm 0.010$	363.93
	Face2Face	$85.11 \pm 1.02$	$0.932 \pm 0.006$	$0.851 \pm 0.010$	$0.853 \pm 0.015$	$0.848 \pm 0.010$	363.93
	FaceShifter	$84.09 \pm 1.22$	$0.919 \pm 0.015$	$0.837 \pm 0.013$	$0.857 \pm 0.011$	$0.819 \pm 0.015$	444.96
	FaceSwap	$80.01 \pm 2.82$	$0.882 \pm 0.034$	$0.826 \pm 0.026$	$0.802 \pm 0.020$	$0.852 \pm 0.032$	113.54
	NeuralTextures	$75.81 \pm 0.75$	$0.836 \pm 0.008$	$0.791 \pm 0.008$	$0.763 \pm 0.003$	$0.821 \pm 0.016$	415.93
*KNN*							
	DeepFakes	$95.03 \pm 0.59$	$0.989 \pm 0.003$	$0.950 \pm 0.006$	$0.952 \pm 0.009$	$0.949 \pm 0.004$	0.02
	Face2Face	$95.03 \pm 0.59$	$0.989 \pm 0.003$	$0.950 \pm 0.006$	$0.952 \pm 0.009$	$0.949 \pm 0.004$	0.02
	FaceShifter	$91.39 \pm 0.68$	$0.973 \pm 0.005$	$0.911 \pm 0.008$	$0.946 \pm 0.006$	$0.879 \pm 0.016$	0.03
	FaceSwap	$76.34 \pm 1.82$	$0.838 \pm 0.014$	$0.795 \pm 0.009$	$0.771 \pm 0.032$	$0.821 \pm 0.017$	0.01
	NeuralTextures	$63.27 \pm 0.84$	$0.691 \pm 0.004$	$0.638 \pm 0.007$	$0.707 \pm 0.013$	$0.581 \pm 0.011$	0.04
*Logistic*							
	DeepFakes	$90.02 \pm 0.84$	$0.964 \pm 0.005$	$0.900 \pm 0.007$	$0.899 \pm 0.020$	$0.902 \pm 0.007$	1.55
	Face2Face	$90.02 \pm 0.84$	$0.964 \pm 0.005$	$0.900 \pm 0.007$	$0.899 \pm 0.020$	$0.902 \pm 0.007$	1.55
	FaceShifter	$89.67 \pm 0.23$	$0.959 \pm 0.003$	$0.896 \pm 0.002$	$0.901 \pm 0.002$	$0.891 \pm 0.004$	4.84
	FaceSwap	$84.52 \pm 1.26$	$0.919 \pm 0.012$	$0.862 \pm 0.014$	$0.859 \pm 0.022$	$0.865 \pm 0.039$	2.76
	NeuralTextures	$81.65 \pm 1.31$	$0.893 \pm 0.006$	$0.837 \pm 0.013$	$0.828 \pm 0.005$	$0.846 \pm 0.023$	4.30
*MLP*							
	DeepFakes	$96.58 \pm 0.19$	$0.993 \pm 0.002$	$0.966 \pm 0.002$	$0.968 \pm 0.009$	$0.964 \pm 0.006$	7.83
	Face2Face	$96.58 \pm 0.19$	$0.993 \pm 0.002$	$0.966 \pm 0.002$	$0.968 \pm 0.009$	$0.964 \pm 0.006$	7.83
	FaceShifter	$95.35 \pm 0.71$	$0.988 \pm 0.002$	$0.953 \pm 0.007$	$0.957 \pm 0.002$	$0.950 \pm 0.013$	15.06
	FaceSwap	$88.93 \pm 2.31$	$0.951 \pm 0.012$	$0.901 \pm 0.020$	$0.898 \pm 0.030$	$0.905 \pm 0.021$	5.35
	NeuralTextures	$88.22 \pm 1.71$	$0.948 \pm 0.006$	$0.894 \pm 0.015$	$0.892 \pm 0.018$	$0.897 \pm 0.019$	16.17
*RandomForest*							
	DeepFakes	$91.32 \pm 0.52$	$0.973 \pm 0.000$	$0.912 \pm 0.005$	$0.925 \pm 0.014$	$0.900 \pm 0.016$	36.28
	Face2Face	$91.32 \pm 0.52$	$0.973 \pm 0.000$	$0.912 \pm 0.005$	$0.925 \pm 0.014$	$0.900 \pm 0.016$	36.28
	FaceShifter	$87.39 \pm 0.76$	$0.946 \pm 0.007$	$0.873 \pm 0.009$	$0.882 \pm 0.007$	$0.864 \pm 0.020$	46.72
	FaceSwap	$80.21 \pm 0.52$	$0.885 \pm 0.006$	$0.833 \pm 0.009$	$0.787 \pm 0.013$	$0.886 \pm 0.036$	11.11
	NeuralTextures	$76.29 \pm 1.66$	$0.845 \pm 0.016$	$0.802 \pm 0.012$	$0.749 \pm 0.019$	$0.863 \pm 0.011$	42.95
*SVM-RBF*							
	DeepFakes	$95.78 \pm 0.46$	$0.992 \pm 0.001$	$0.958 \pm 0.005$	$0.964 \pm 0.006$	$0.951 \pm 0.003$	469.06
	Face2Face	$95.78 \pm 0.46$	$0.992 \pm 0.001$	$0.958 \pm 0.005$	$0.964 \pm 0.006$	$0.951 \pm 0.003$	469.06
	FaceShifter	$94.87 \pm 1.21$	$0.985 \pm 0.004$	$0.949 \pm 0.013$	$0.948 \pm 0.002$	$0.950 \pm 0.023$	839.90
	FaceSwap	$87.53 \pm 1.72$	$0.949 \pm 0.014$	$0.891 \pm 0.015$	$0.869 \pm 0.024$	$0.914 \pm 0.023$	54.52
	NeuralTextures	$85.61 \pm 1.15$	$0.931 \pm 0.006$	$0.874 \pm 0.012$	$0.854 \pm 0.007$	$0.894 \pm 0.024$	724.48
*XGBoost*							
	DeepFakes	$93.93 \pm 0.18$	$0.986 \pm 0.002$	$0.939 \pm 0.002$	$0.944 \pm 0.006$	$0.934 \pm 0.008$	12.43
	Face2Face	$93.93 \pm 0.18$	$0.986 \pm 0.002$	$0.939 \pm 0.002$	$0.944 \pm 0.006$	$0.934 \pm 0.008$	12.43
	FaceShifter	$92.30 \pm 1.59$	$0.977 \pm 0.010$	$0.922 \pm 0.017$	$0.931 \pm 0.007$	$0.914 \pm 0.027$	4.37
	FaceSwap	$84.71 \pm 1.46$	$0.928 \pm 0.018$	$0.865 \pm 0.012$	$0.851 \pm 0.017$	$0.881 \pm 0.009$	2.46
	NeuralTextures	$82.76 \pm 1.00$	$0.906 \pm 0.006$	$0.848 \pm 0.009$	$0.833 \pm 0.010$	$0.864 \pm 0.012$	4.06
**Average**		**84.59**	**0.903**	**0.855**	−	−	−

Method: ga+lasso; Values shown as Mean ± 95% Confidence Interval; FaceForensics++ C23 compression level.

**Table 22:** Performance Results for extratrees Feature Selection (FF++ c23 dataset).

**Classifier**	**Manipulation**	**Acc (%)**	**AUC**	**F1**	**Precision**	**Recall**	**Time (s)**
*AdaBoost*							
	DeepFakes	$77.62 \pm 0.85$	$0.861 \pm 0.003$	$0.773 \pm 0.004$	$0.783 \pm 0.030$	$0.764 \pm 0.031$	5.61
	Face2Face	$77.62 \pm 0.85$	$0.861 \pm 0.003$	$0.773 \pm 0.004$	$0.783 \pm 0.030$	$0.764 \pm 0.031$	5.61
	FaceShifter	$76.40 \pm 1.29$	$0.844 \pm 0.016$	$0.758 \pm 0.022$	$0.778 \pm 0.023$	$0.740 \pm 0.055$	6.77
	FaceSwap	$73.71 \pm 1.18$	$0.809 \pm 0.015$	$0.769 \pm 0.012$	$0.756 \pm 0.031$	$0.782 \pm 0.048$	2.03
	NeuralTextures	$71.79 \pm 0.89$	$0.785 \pm 0.018$	$0.752 \pm 0.002$	$0.736 \pm 0.019$	$0.770 \pm 0.019$	6.39
*DecisionTree*							
	DeepFakes	$75.83 \pm 0.76$	$0.758 \pm 0.008$	$0.759 \pm 0.010$	$0.758 \pm 0.011$	$0.759 \pm 0.025$	1.29
	Face2Face	$75.83 \pm 0.76$	$0.758 \pm 0.008$	$0.759 \pm 0.010$	$0.758 \pm 0.011$	$0.759 \pm 0.025$	1.29
	FaceShifter	$74.08 \pm 0.84$	$0.741 \pm 0.008$	$0.742 \pm 0.010$	$0.740 \pm 0.009$	$0.743 \pm 0.019$	1.59
	FaceSwap	$66.93 \pm 3.23$	$0.666 \pm 0.037$	$0.702 \pm 0.021$	$0.707 \pm 0.043$	$0.697 \pm 0.024$	0.41
	NeuralTextures	$67.35 \pm 2.50$	$0.670 \pm 0.025$	$0.705 \pm 0.024$	$0.709 \pm 0.021$	$0.702 \pm 0.028$	1.41
*ExtraTrees*							
	DeepFakes	$89.92 \pm 0.28$	$0.965 \pm 0.003$	$0.897 \pm 0.001$	$0.915 \pm 0.017$	$0.880 \pm 0.014$	1.56
	Face2Face	$89.92 \pm 0.28$	$0.965 \pm 0.003$	$0.897 \pm 0.001$	$0.915 \pm 0.017$	$0.880 \pm 0.014$	1.56
	FaceShifter	$87.69 \pm 0.29$	$0.949 \pm 0.004$	$0.874 \pm 0.002$	$0.895 \pm 0.011$	$0.854 \pm 0.008$	2.24
	FaceSwap	$80.09 \pm 0.38$	$0.884 \pm 0.014$	$0.826 \pm 0.007$	$0.807 \pm 0.010$	$0.846 \pm 0.025$	0.72
	NeuralTextures	$80.13 \pm 1.13$	$0.885 \pm 0.011$	$0.827 \pm 0.009$	$0.801 \pm 0.017$	$0.855 \pm 0.015$	2.28
*GradientBoosting*							
	DeepFakes	$82.82 \pm 0.31$	$0.912 \pm 0.007$	$0.827 \pm 0.003$	$0.832 \pm 0.015$	$0.822 \pm 0.017$	30.33
	Face2Face	$82.82 \pm 0.31$	$0.912 \pm 0.007$	$0.827 \pm 0.003$	$0.832 \pm 0.015$	$0.822 \pm 0.017$	30.33
	FaceShifter	$81.23 \pm 0.61$	$0.896 \pm 0.009$	$0.807 \pm 0.010$	$0.829 \pm 0.007$	$0.787 \pm 0.026$	36.41
	FaceSwap	$77.29 \pm 1.07$	$0.851 \pm 0.016$	$0.801 \pm 0.009$	$0.783 \pm 0.014$	$0.820 \pm 0.014$	10.69
	NeuralTextures	$74.71 \pm 1.69$	$0.823 \pm 0.013$	$0.780 \pm 0.016$	$0.756 \pm 0.013$	$0.805 \pm 0.020$	34.38
*KNN*							
	DeepFakes	$97.27 \pm 0.27$	$0.995 \pm 0.002$	$0.972 \pm 0.003$	$0.984 \pm 0.006$	$0.961 \pm 0.006$	0.00
	Face2Face	$97.27 \pm 0.27$	$0.995 \pm 0.002$	$0.972 \pm 0.003$	$0.984 \pm 0.006$	$0.961 \pm 0.006$	0.00
	FaceShifter	$94.59 \pm 0.87$	$0.986 \pm 0.002$	$0.945 \pm 0.009$	$0.965 \pm 0.009$	$0.925 \pm 0.015$	0.00
	FaceSwap	$85.29 \pm 0.70$	$0.922 \pm 0.008$	$0.870 \pm 0.007$	$0.860 \pm 0.012$	$0.879 \pm 0.019$	0.00
	NeuralTextures	$79.16 \pm 1.40$	$0.872 \pm 0.016$	$0.803 \pm 0.014$	$0.848 \pm 0.011$	$0.762 \pm 0.016$	0.00
*Logistic*							
	DeepFakes	$80.88 \pm 0.80$	$0.891 \pm 0.004$	$0.809 \pm 0.003$	$0.808 \pm 0.026$	$0.810 \pm 0.022$	0.20
	Face2Face	$80.88 \pm 0.80$	$0.891 \pm 0.004$	$0.809 \pm 0.003$	$0.808 \pm 0.026$	$0.810 \pm 0.022$	0.20
	FaceShifter	$79.12 \pm 0.96$	$0.864 \pm 0.008$	$0.787 \pm 0.010$	$0.802 \pm 0.010$	$0.773 \pm 0.013$	1.11
	FaceSwap	$75.97 \pm 2.16$	$0.834 \pm 0.026$	$0.789 \pm 0.015$	$0.774 \pm 0.033$	$0.804 \pm 0.025$	0.72
	NeuralTextures	$73.46 \pm 0.86$	$0.804 \pm 0.006$	$0.767 \pm 0.009$	$0.750 \pm 0.003$	$0.786 \pm 0.016$	0.98
*MLP*							
	DeepFakes	$90.76 \pm 0.89$	$0.968 \pm 0.001$	$0.908 \pm 0.010$	$0.907 \pm 0.016$	$0.908 \pm 0.026$	9.76
	Face2Face	$90.76 \pm 0.89$	$0.968 \pm 0.001$	$0.908 \pm 0.010$	$0.907 \pm 0.016$	$0.908 \pm 0.026$	9.76
	FaceShifter	$88.21 \pm 0.46$	$0.951 \pm 0.002$	$0.882 \pm 0.005$	$0.884 \pm 0.005$	$0.880 \pm 0.007$	33.12
	FaceSwap	$81.39 \pm 1.27$	$0.890 \pm 0.024$	$0.834 \pm 0.008$	$0.833 \pm 0.031$	$0.835 \pm 0.027$	9.45
	NeuralTextures	$79.26 \pm 0.41$	$0.873 \pm 0.012$	$0.813 \pm 0.005$	$0.817 \pm 0.004$	$0.809 \pm 0.013$	42.80
*RandomForest*							
	DeepFakes	$88.07 \pm 0.82$	$0.954 \pm 0.003$	$0.879 \pm 0.007$	$0.892 \pm 0.019$	$0.867 \pm 0.008$	10.69
	Face2Face	$88.07 \pm 0.82$	$0.954 \pm 0.003$	$0.879 \pm 0.007$	$0.892 \pm 0.019$	$0.867 \pm 0.008$	10.69
	FaceShifter	$86.04 \pm 0.79$	$0.938 \pm 0.004$	$0.857 \pm 0.009$	$0.877 \pm 0.007$	$0.839 \pm 0.019$	14.10
	FaceSwap	$79.15 \pm 2.52$	$0.869 \pm 0.017$	$0.819 \pm 0.019$	$0.795 \pm 0.032$	$0.845 \pm 0.016$	3.62
	NeuralTextures	$79.01 \pm 1.20$	$0.870 \pm 0.013$	$0.817 \pm 0.011$	$0.792 \pm 0.016$	$0.845 \pm 0.023$	12.83
*SVM-RBF*							
	DeepFakes	$92.40 \pm 0.87$	$0.976 \pm 0.003$	$0.924 \pm 0.008$	$0.930 \pm 0.018$	$0.918 \pm 0.002$	24.61
	Face2Face	$92.40 \pm 0.87$	$0.976 \pm 0.003$	$0.924 \pm 0.008$	$0.930 \pm 0.018$	$0.918 \pm 0.002$	24.61
	FaceShifter	$89.88 \pm 0.95$	$0.961 \pm 0.005$	$0.897 \pm 0.011$	$0.916 \pm 0.004$	$0.878 \pm 0.025$	61.18
	FaceSwap	$82.97 \pm 1.74$	$0.904 \pm 0.022$	$0.851 \pm 0.014$	$0.834 \pm 0.030$	$0.868 \pm 0.030$	7.47
	NeuralTextures	$82.24 \pm 0.48$	$0.898 \pm 0.012$	$0.843 \pm 0.005$	$0.828 \pm 0.006$	$0.859 \pm 0.011$	74.81
*XGBoost*							
	DeepFakes	$90.17 \pm 0.84$	$0.966 \pm 0.005$	$0.901 \pm 0.008$	$0.906 \pm 0.016$	$0.897 \pm 0.009$	0.65
	Face2Face	$90.17 \pm 0.84$	$0.966 \pm 0.005$	$0.901 \pm 0.008$	$0.906 \pm 0.016$	$0.897 \pm 0.009$	0.65
	FaceShifter	$87.84 \pm 1.78$	$0.950 \pm 0.008$	$0.877 \pm 0.020$	$0.889 \pm 0.009$	$0.865 \pm 0.032$	0.38
	FaceSwap	$80.18 \pm 3.40$	$0.885 \pm 0.028$	$0.824 \pm 0.029$	$0.816 \pm 0.036$	$0.832 \pm 0.034$	0.26
	NeuralTextures	$80.64 \pm 1.81$	$0.884 \pm 0.016$	$0.828 \pm 0.015$	$0.819 \pm 0.020$	$0.838 \pm 0.010$	0.32
**Average**		**82.55**	**0.889**	**0.835**	−	−	−

Method: extratrees; Values shown as Mean ± 95% Confidence Interval; FaceForensics++ C23 compression level.

**Table 23:** Performance Results for extratrees+lasso Feature Selection (FF++ c23 dataset).

**Classifier**	**Manipulation**	**Acc (%)**	**AUC**	**F1**	**Precision**	**Recall**	**Time (s)**
*AdaBoost*							
	DeepFakes	$77.41 \pm 0.85$	$0.861 \pm 0.003$	$0.771 \pm 0.005$	$0.781 \pm 0.030$	$0.762 \pm 0.032$	5.26
	Face2Face	$77.41 \pm 0.85$	$0.861 \pm 0.003$	$0.771 \pm 0.005$	$0.781 \pm 0.030$	$0.762 \pm 0.032$	5.26
	FaceShifter	$76.84 \pm 0.95$	$0.847 \pm 0.011$	$0.762 \pm 0.011$	$0.784 \pm 0.024$	$0.742 \pm 0.031$	6.33
	FaceSwap	$73.50 \pm 2.91$	$0.811 \pm 0.021$	$0.768 \pm 0.033$	$0.751 \pm 0.013$	$0.786 \pm 0.061$	1.90
	NeuralTextures	$71.63 \pm 1.06$	$0.784 \pm 0.018$	$0.751 \pm 0.006$	$0.734 \pm 0.018$	$0.770 \pm 0.017$	5.87
*DecisionTree*							
	DeepFakes	$75.65 \pm 1.37$	$0.757 \pm 0.014$	$0.757 \pm 0.017$	$0.755 \pm 0.008$	$0.760 \pm 0.028$	1.22
	Face2Face	$75.65 \pm 1.37$	$0.757 \pm 0.014$	$0.757 \pm 0.017$	$0.755 \pm 0.008$	$0.760 \pm 0.028$	1.22
	FaceShifter	$74.10 \pm 0.53$	$0.741 \pm 0.005$	$0.741 \pm 0.006$	$0.741 \pm 0.005$	$0.742 \pm 0.010$	1.49
	FaceSwap	$66.69 \pm 0.38$	$0.663 \pm 0.004$	$0.701 \pm 0.010$	$0.703 \pm 0.010$	$0.700 \pm 0.028$	0.36
	NeuralTextures	$67.39 \pm 0.53$	$0.670 \pm 0.005$	$0.707 \pm 0.006$	$0.707 \pm 0.004$	$0.706 \pm 0.010$	1.30
*ExtraTrees*							
	DeepFakes	$89.67 \pm 1.01$	$0.964 \pm 0.004$	$0.895 \pm 0.010$	$0.912 \pm 0.022$	$0.878 \pm 0.016$	1.44
	Face2Face	$89.67 \pm 1.01$	$0.964 \pm 0.004$	$0.895 \pm 0.010$	$0.912 \pm 0.022$	$0.878 \pm 0.016$	1.44
	FaceShifter	$87.64 \pm 0.29$	$0.949 \pm 0.002$	$0.873 \pm 0.004$	$0.897 \pm 0.006$	$0.851 \pm 0.011$	2.06
	FaceSwap	$79.84 \pm 2.03$	$0.883 \pm 0.012$	$0.824 \pm 0.023$	$0.804 \pm 0.014$	$0.845 \pm 0.054$	0.68
	NeuralTextures	$80.00 \pm 0.71$	$0.885 \pm 0.009$	$0.826 \pm 0.009$	$0.800 \pm 0.003$	$0.854 \pm 0.022$	2.10
*GradientBoosting*							
	DeepFakes	$83.03 \pm 0.60$	$0.913 \pm 0.003$	$0.829 \pm 0.007$	$0.835 \pm 0.009$	$0.823 \pm 0.015$	28.38
	Face2Face	$83.03 \pm 0.60$	$0.913 \pm 0.003$	$0.829 \pm 0.007$	$0.835 \pm 0.009$	$0.823 \pm 0.015$	28.38
	FaceShifter	$81.66 \pm 0.40$	$0.897 \pm 0.007$	$0.812 \pm 0.007$	$0.834 \pm 0.010$	$0.792 \pm 0.020$	34.05
	FaceSwap	$77.32 \pm 1.53$	$0.851 \pm 0.024$	$0.802 \pm 0.013$	$0.784 \pm 0.022$	$0.821 \pm 0.025$	9.98
	NeuralTextures	$74.78 \pm 1.49$	$0.823 \pm 0.014$	$0.780 \pm 0.013$	$0.757 \pm 0.012$	$0.805 \pm 0.014$	31.63
*KNN*							
	DeepFakes	$97.05 \pm 0.17$	$0.995 \pm 0.003$	$0.970 \pm 0.002$	$0.983 \pm 0.005$	$0.958 \pm 0.008$	0.00
	Face2Face	$97.05 \pm 0.17$	$0.995 \pm 0.003$	$0.970 \pm 0.002$	$0.983 \pm 0.005$	$0.958 \pm 0.008$	0.00
	FaceShifter	$94.54 \pm 0.75$	$0.985 \pm 0.001$	$0.944 \pm 0.008$	$0.965 \pm 0.008$	$0.925 \pm 0.015$	0.00
	FaceSwap	$84.91 \pm 1.45$	$0.919 \pm 0.010$	$0.866 \pm 0.010$	$0.858 \pm 0.027$	$0.874 \pm 0.010$	0.00
	NeuralTextures	$79.31 \pm 2.31$	$0.872 \pm 0.023$	$0.804 \pm 0.023$	$0.849 \pm 0.018$	$0.764 \pm 0.028$	0.00
*Logistic*							
	DeepFakes	$80.86 \pm 0.43$	$0.890 \pm 0.004$	$0.809 \pm 0.003$	$0.808 \pm 0.021$	$0.810 \pm 0.024$	0.19
	Face2Face	$80.86 \pm 0.43$	$0.890 \pm 0.004$	$0.809 \pm 0.003$	$0.808 \pm 0.021$	$0.810 \pm 0.024$	0.19
	FaceShifter	$79.12 \pm 0.86$	$0.864 \pm 0.008$	$0.787 \pm 0.009$	$0.802 \pm 0.010$	$0.773 \pm 0.012$	0.82
	FaceSwap	$75.98 \pm 2.83$	$0.834 \pm 0.025$	$0.789 \pm 0.020$	$0.775 \pm 0.038$	$0.804 \pm 0.018$	0.82
	NeuralTextures	$73.50 \pm 0.64$	$0.804 \pm 0.006$	$0.768 \pm 0.007$	$0.749 \pm 0.002$	$0.787 \pm 0.013$	0.88
*MLP*							
	DeepFakes	$90.51 \pm 0.53$	$0.966 \pm 0.004$	$0.905 \pm 0.006$	$0.907 \pm 0.011$	$0.903 \pm 0.018$	10.79
	Face2Face	$90.51 \pm 0.53$	$0.966 \pm 0.004$	$0.905 \pm 0.006$	$0.907 \pm 0.011$	$0.903 \pm 0.018$	10.79
	FaceShifter	$87.92 \pm 0.27$	$0.947 \pm 0.003$	$0.879 \pm 0.001$	$0.881 \pm 0.016$	$0.877 \pm 0.014$	34.04
	FaceSwap	$80.43 \pm 0.57$	$0.881 \pm 0.005$	$0.826 \pm 0.004$	$0.822 \pm 0.010$	$0.829 \pm 0.003$	10.28
	NeuralTextures	$79.19 \pm 1.04$	$0.873 \pm 0.013$	$0.812 \pm 0.011$	$0.816 \pm 0.005$	$0.808 \pm 0.020$	40.62
*RandomForest*							
	DeepFakes	$87.96 \pm 0.75$	$0.953 \pm 0.002$	$0.878 \pm 0.007$	$0.891 \pm 0.020$	$0.865 \pm 0.015$	9.32
	Face2Face	$87.96 \pm 0.75$	$0.953 \pm 0.002$	$0.878 \pm 0.007$	$0.891 \pm 0.020$	$0.865 \pm 0.015$	9.32
	FaceShifter	$86.18 \pm 0.63$	$0.938 \pm 0.005$	$0.859 \pm 0.007$	$0.877 \pm 0.002$	$0.842 \pm 0.013$	12.08
	FaceSwap	$79.05 \pm 1.62$	$0.869 \pm 0.021$	$0.820 \pm 0.015$	$0.789 \pm 0.020$	$0.852 \pm 0.032$	3.10
	NeuralTextures	$79.02 \pm 1.45$	$0.871 \pm 0.016$	$0.818 \pm 0.014$	$0.791 \pm 0.014$	$0.846 \pm 0.024$	11.09
*SVM-RBF*							
	DeepFakes	$92.18 \pm 0.91$	$0.975 \pm 0.003$	$0.921 \pm 0.008$	$0.929 \pm 0.026$	$0.914 \pm 0.011$	27.97
	Face2Face	$92.18 \pm 0.91$	$0.975 \pm 0.003$	$0.921 \pm 0.008$	$0.929 \pm 0.026$	$0.914 \pm 0.011$	27.97
	FaceShifter	$89.72 \pm 1.04$	$0.960 \pm 0.005$	$0.895 \pm 0.012$	$0.914 \pm 0.006$	$0.877 \pm 0.029$	69.96
	FaceSwap	$82.69 \pm 1.83$	$0.901 \pm 0.025$	$0.848 \pm 0.014$	$0.830 \pm 0.030$	$0.867 \pm 0.026$	8.39
	NeuralTextures	$82.03 \pm 1.22$	$0.895 \pm 0.012$	$0.841 \pm 0.011$	$0.827 \pm 0.010$	$0.856 \pm 0.016$	85.28
*XGBoost*							
	DeepFakes	$89.81 \pm 0.48$	$0.964 \pm 0.002$	$0.898 \pm 0.003$	$0.902 \pm 0.019$	$0.893 \pm 0.014$	1.06
	Face2Face	$89.81 \pm 0.48$	$0.964 \pm 0.002$	$0.898 \pm 0.003$	$0.902 \pm 0.019$	$0.893 \pm 0.014$	1.06
	FaceShifter	$87.86 \pm 0.86$	$0.950 \pm 0.005$	$0.877 \pm 0.011$	$0.891 \pm 0.005$	$0.863 \pm 0.024$	0.34
	FaceSwap	$80.91 \pm 2.49$	$0.884 \pm 0.020$	$0.830 \pm 0.020$	$0.824 \pm 0.033$	$0.837 \pm 0.023$	0.26
	NeuralTextures	$80.34 \pm 1.46$	$0.881 \pm 0.011$	$0.825 \pm 0.015$	$0.816 \pm 0.008$	$0.835 \pm 0.023$	0.25
**Average**		**82.45**	**0.888**	**0.834**	−	−	−

Method: extratrees+lasso. Values shown as Mean ± 95% Confidence Interval. FaceForensics++ C23 compression level.

**Table 24:** Performance Results for kbest Feature Selection (FF++ c23 dataset).

**Classifier**	**Manipulation**	**Acc (%)**	**AUC**	**F1**	**Precision**	**Recall**	**Time (s)**
*AdaBoost*							
	DeepFakes	$77.34 \pm 0.19$	$0.857 \pm 0.003$	$0.771 \pm 0.005$	$0.779 \pm 0.009$	$0.764 \pm 0.018$	5.65
	Face2Face	$77.34 \pm 0.19$	$0.857 \pm 0.003$	$0.771 \pm 0.005$	$0.779 \pm 0.009$	$0.764 \pm 0.018$	5.65
	FaceShifter	$75.08 \pm 0.90$	$0.830 \pm 0.006$	$0.748 \pm 0.002$	$0.758 \pm 0.031$	$0.739 \pm 0.033$	6.53
	FaceSwap	$71.75 \pm 0.48$	$0.785 \pm 0.011$	$0.751 \pm 0.006$	$0.740 \pm 0.003$	$0.761 \pm 0.011$	1.95
	NeuralTextures	$71.26 \pm 0.58$	$0.779 \pm 0.019$	$0.747 \pm 0.013$	$0.732 \pm 0.012$	$0.763 \pm 0.038$	5.96
*DecisionTree*							
	DeepFakes	$74.84 \pm 1.30$	$0.748 \pm 0.013$	$0.748 \pm 0.017$	$0.749 \pm 0.006$	$0.748 \pm 0.031$	1.26
	Face2Face	$74.84 \pm 1.30$	$0.748 \pm 0.013$	$0.748 \pm 0.017$	$0.749 \pm 0.006$	$0.748 \pm 0.031$	1.26
	FaceShifter	$73.81 \pm 0.72$	$0.738 \pm 0.007$	$0.737 \pm 0.006$	$0.740 \pm 0.010$	$0.735 \pm 0.002$	1.51
	FaceSwap	$64.93 \pm 1.38$	$0.645 \pm 0.010$	$0.686 \pm 0.024$	$0.686 \pm 0.011$	$0.685 \pm 0.052$	0.37
	NeuralTextures	$66.48 \pm 1.41$	$0.660 \pm 0.014$	$0.699 \pm 0.014$	$0.699 \pm 0.010$	$0.700 \pm 0.019$	1.31
*ExtraTrees*							
	DeepFakes	$88.24 \pm 0.15$	$0.955 \pm 0.006$	$0.880 \pm 0.003$	$0.900 \pm 0.014$	$0.860 \pm 0.016$	1.63
	Face2Face	$88.24 \pm 0.15$	$0.955 \pm 0.006$	$0.880 \pm 0.003$	$0.900 \pm 0.014$	$0.860 \pm 0.016$	1.63
	FaceShifter	$85.42 \pm 0.53$	$0.934 \pm 0.004$	$0.851 \pm 0.004$	$0.871 \pm 0.013$	$0.831 \pm 0.007$	2.27
	FaceSwap	$77.95 \pm 2.11$	$0.863 \pm 0.016$	$0.809 \pm 0.013$	$0.783 \pm 0.034$	$0.838 \pm 0.020$	0.74
	NeuralTextures	$79.10 \pm 2.37$	$0.874 \pm 0.023$	$0.820 \pm 0.020$	$0.788 \pm 0.024$	$0.855 \pm 0.020$	2.29
*GradientBoosting*							
	DeepFakes	$82.00 \pm 0.75$	$0.904 \pm 0.007$	$0.818 \pm 0.007$	$0.828 \pm 0.016$	$0.808 \pm 0.018$	30.13
	Face2Face	$82.00 \pm 0.75$	$0.904 \pm 0.007$	$0.818 \pm 0.007$	$0.828 \pm 0.016$	$0.808 \pm 0.018$	30.13
	FaceShifter	$79.57 \pm 0.37$	$0.878 \pm 0.003$	$0.791 \pm 0.005$	$0.812 \pm 0.004$	$0.771 \pm 0.011$	35.14
	FaceSwap	$74.97 \pm 2.64$	$0.825 \pm 0.023$	$0.783 \pm 0.019$	$0.760 \pm 0.031$	$0.807 \pm 0.014$	10.24
	NeuralTextures	$73.82 \pm 1.13$	$0.813 \pm 0.015$	$0.773 \pm 0.012$	$0.747 \pm 0.004$	$0.800 \pm 0.022$	32.09
*KNN*							
	DeepFakes	$96.31 \pm 0.73$	$0.992 \pm 0.001$	$0.962 \pm 0.008$	$0.978 \pm 0.001$	$0.948 \pm 0.015$	0.00
	Face2Face	$96.31 \pm 0.73$	$0.992 \pm 0.001$	$0.962 \pm 0.008$	$0.978 \pm 0.001$	$0.948 \pm 0.015$	0.00
	FaceShifter	$92.99 \pm 0.96$	$0.978 \pm 0.003$	$0.928 \pm 0.010$	$0.952 \pm 0.014$	$0.905 \pm 0.006$	0.00
	FaceSwap	$80.91 \pm 2.64$	$0.886 \pm 0.021$	$0.833 \pm 0.021$	$0.814 \pm 0.033$	$0.854 \pm 0.020$	0.00
	NeuralTextures	$76.81 \pm 2.22$	$0.845 \pm 0.026$	$0.780 \pm 0.025$	$0.825 \pm 0.014$	$0.740 \pm 0.038$	0.00
*Logistic*							
	DeepFakes	$79.97 \pm 0.62$	$0.880 \pm 0.010$	$0.799 \pm 0.011$	$0.802 \pm 0.011$	$0.797 \pm 0.032$	0.20
	Face2Face	$79.97 \pm 0.62$	$0.880 \pm 0.010$	$0.799 \pm 0.011$	$0.802 \pm 0.011$	$0.797 \pm 0.032$	0.20
	FaceShifter	$77.38 \pm 0.91$	$0.850 \pm 0.006$	$0.769 \pm 0.009$	$0.786 \pm 0.014$	$0.753 \pm 0.013$	0.97
	FaceSwap	$73.63 \pm 2.94$	$0.803 \pm 0.028$	$0.770 \pm 0.021$	$0.750 \pm 0.036$	$0.792 \pm 0.008$	0.50
	NeuralTextures	$72.66 \pm 0.42$	$0.795 \pm 0.011$	$0.761 \pm 0.005$	$0.741 \pm 0.001$	$0.783 \pm 0.010$	0.78
*MLP*							
	DeepFakes	$89.43 \pm 1.08$	$0.957 \pm 0.002$	$0.894 \pm 0.010$	$0.895 \pm 0.017$	$0.894 \pm 0.003$	12.54
	Face2Face	$89.43 \pm 1.08$	$0.957 \pm 0.002$	$0.894 \pm 0.010$	$0.895 \pm 0.017$	$0.894 \pm 0.003$	12.54
	FaceShifter	$86.24 \pm 0.47$	$0.933 \pm 0.006$	$0.862 \pm 0.004$	$0.866 \pm 0.007$	$0.857 \pm 0.002$	37.27
	FaceSwap	$77.56 \pm 1.77$	$0.856 \pm 0.015$	$0.801 \pm 0.013$	$0.793 \pm 0.025$	$0.810 \pm 0.011$	10.46
	NeuralTextures	$77.49 \pm 1.03$	$0.850 \pm 0.014$	$0.797 \pm 0.009$	$0.801 \pm 0.009$	$0.792 \pm 0.009$	37.03
*RandomForest*							
	DeepFakes	$86.70 \pm 0.84$	$0.943 \pm 0.004$	$0.865 \pm 0.009$	$0.881 \pm 0.014$	$0.849 \pm 0.015$	10.79
	Face2Face	$86.70 \pm 0.84$	$0.943 \pm 0.004$	$0.865 \pm 0.009$	$0.881 \pm 0.014$	$0.849 \pm 0.015$	10.79
	FaceShifter	$84.18 \pm 0.51$	$0.923 \pm 0.005$	$0.838 \pm 0.007$	$0.859 \pm 0.006$	$0.819 \pm 0.019$	13.50
	FaceSwap	$76.81 \pm 2.26$	$0.847 \pm 0.021$	$0.802 \pm 0.015$	$0.768 \pm 0.033$	$0.839 \pm 0.026$	3.46
	NeuralTextures	$78.04 \pm 1.71$	$0.861 \pm 0.014$	$0.810 \pm 0.016$	$0.782 \pm 0.017$	$0.839 \pm 0.026$	11.99
*SVM-RBF*							
	DeepFakes	$91.02 \pm 1.08$	$0.969 \pm 0.005$	$0.909 \pm 0.011$	$0.919 \pm 0.015$	$0.899 \pm 0.014$	26.48
	Face2Face	$91.02 \pm 1.08$	$0.969 \pm 0.005$	$0.909 \pm 0.011$	$0.919 \pm 0.015$	$0.899 \pm 0.014$	26.48
	FaceShifter	$87.96 \pm 0.38$	$0.947 \pm 0.005$	$0.877 \pm 0.003$	$0.898 \pm 0.009$	$0.857 \pm 0.003$	67.07
	FaceSwap	$79.82 \pm 2.09$	$0.874 \pm 0.030$	$0.825 \pm 0.015$	$0.800 \pm 0.028$	$0.852 \pm 0.011$	8.29
	NeuralTextures	$79.82 \pm 1.16$	$0.875 \pm 0.018$	$0.822 \pm 0.012$	$0.806 \pm 0.005$	$0.840 \pm 0.023$	79.83
*XGBoost*							
	DeepFakes	$88.89 \pm 1.47$	$0.959 \pm 0.008$	$0.888 \pm 0.016$	$0.898 \pm 0.013$	$0.877 \pm 0.023$	1.68
	Face2Face	$88.89 \pm 1.47$	$0.959 \pm 0.008$	$0.888 \pm 0.016$	$0.898 \pm 0.013$	$0.877 \pm 0.023$	1.68
	FaceShifter	$86.26 \pm 0.72$	$0.938 \pm 0.004$	$0.861 \pm 0.006$	$0.872 \pm 0.016$	$0.850 \pm 0.005$	0.33
	FaceSwap	$78.36 \pm 1.84$	$0.861 \pm 0.018$	$0.809 \pm 0.014$	$0.796 \pm 0.031$	$0.823 \pm 0.029$	0.29
	NeuralTextures	$78.61 \pm 2.95$	$0.869 \pm 0.022$	$0.811 \pm 0.025$	$0.798 \pm 0.030$	$0.824 \pm 0.019$	0.32
**Average**		**80.98**	**0.875**	**0.820**	−	−	−

Method: kbest; Values shown as Mean ± 95% Confidence Interval; FaceForensics++ C23 compression level.

**Table 25:** Performance Results for kbest+lasso Feature Selection (FF++ c23 dataset).

**Classifier**	**Manipulation**	**Acc (%)**	**AUC**	**F1**	**Precision**	**Recall**	**Time (s)**
*AdaBoost*							
	DeepFakes	$77.25 \pm 0.22$	$0.856 \pm 0.005$	$0.770 \pm 0.004$	$0.778 \pm 0.009$	$0.763 \pm 0.016$	5.22
	Face2Face	$77.25 \pm 0.22$	$0.856 \pm 0.005$	$0.770 \pm 0.004$	$0.778 \pm 0.009$	$0.763 \pm 0.016$	5.22
	FaceShifter	$74.97 \pm 1.36$	$0.830 \pm 0.008$	$0.747 \pm 0.003$	$0.756 \pm 0.035$	$0.737 \pm 0.028$	6.08
	FaceSwap	$71.65 \pm 0.90$	$0.785 \pm 0.012$	$0.751 \pm 0.002$	$0.736 \pm 0.019$	$0.767 \pm 0.016$	1.78
	NeuralTextures	$71.26 \pm 0.58$	$0.779 \pm 0.019$	$0.747 \pm 0.013$	$0.732 \pm 0.012$	$0.763 \pm 0.038$	5.95
*DecisionTree*							
	DeepFakes	$74.66 \pm 1.37$	$0.747 \pm 0.014$	$0.746 \pm 0.017$	$0.748 \pm 0.007$	$0.745 \pm 0.028$	1.18
	Face2Face	$74.66 \pm 1.37$	$0.747 \pm 0.014$	$0.746 \pm 0.017$	$0.748 \pm 0.007$	$0.745 \pm 0.028$	1.18
	FaceShifter	$73.07 \pm 1.07$	$0.731 \pm 0.011$	$0.730 \pm 0.014$	$0.733 \pm 0.010$	$0.727 \pm 0.026$	1.40
	FaceSwap	$64.77 \pm 0.60$	$0.643 \pm 0.009$	$0.684 \pm 0.004$	$0.685 \pm 0.014$	$0.682 \pm 0.019$	0.34
	NeuralTextures	$66.33 \pm 0.77$	$0.659 \pm 0.009$	$0.697 \pm 0.004$	$0.699 \pm 0.012$	$0.695 \pm 0.005$	1.31
*ExtraTrees*							
	DeepFakes	$87.96 \pm 0.85$	$0.953 \pm 0.007$	$0.877 \pm 0.006$	$0.898 \pm 0.030$	$0.857 \pm 0.017$	1.47
	Face2Face	$87.96 \pm 0.85$	$0.953 \pm 0.007$	$0.877 \pm 0.006$	$0.898 \pm 0.030$	$0.857 \pm 0.017$	1.47
	FaceShifter	$85.26 \pm 0.54$	$0.932 \pm 0.008$	$0.849 \pm 0.005$	$0.870 \pm 0.008$	$0.830 \pm 0.006$	2.10
	FaceSwap	$77.92 \pm 1.40$	$0.860 \pm 0.031$	$0.809 \pm 0.009$	$0.781 \pm 0.021$	$0.840 \pm 0.011$	0.69
	NeuralTextures	$79.05 \pm 2.22$	$0.875 \pm 0.024$	$0.819 \pm 0.018$	$0.788 \pm 0.023$	$0.854 \pm 0.019$	2.29
*GradientBoosting*							
	DeepFakes	$81.81 \pm 1.12$	$0.903 \pm 0.006$	$0.816 \pm 0.012$	$0.826 \pm 0.015$	$0.806 \pm 0.023$	28.04
	Face2Face	$81.81 \pm 1.12$	$0.903 \pm 0.006$	$0.816 \pm 0.012$	$0.826 \pm 0.015$	$0.806 \pm 0.023$	28.04
	FaceShifter	$79.57 \pm 0.51$	$0.878 \pm 0.003$	$0.790 \pm 0.008$	$0.811 \pm 0.010$	$0.771 \pm 0.020$	32.62
	FaceSwap	$75.31 \pm 1.81$	$0.825 \pm 0.022$	$0.785 \pm 0.009$	$0.764 \pm 0.033$	$0.807 \pm 0.024$	9.36
	NeuralTextures	$73.65 \pm 0.71$	$0.812 \pm 0.014$	$0.771 \pm 0.008$	$0.746 \pm 0.003$	$0.798 \pm 0.017$	31.88
*KNN*							
	DeepFakes	$96.07 \pm 0.72$	$0.991 \pm 0.002$	$0.960 \pm 0.008$	$0.975 \pm 0.002$	$0.945 \pm 0.014$	0.00
	Face2Face	$96.07 \pm 0.72$	$0.991 \pm 0.002$	$0.960 \pm 0.008$	$0.975 \pm 0.002$	$0.945 \pm 0.014$	0.00
	FaceShifter	$92.64 \pm 0.67$	$0.977 \pm 0.006$	$0.925 \pm 0.007$	$0.948 \pm 0.008$	$0.903 \pm 0.008$	0.00
	FaceSwap	$80.79 \pm 2.95$	$0.884 \pm 0.021$	$0.832 \pm 0.023$	$0.814 \pm 0.034$	$0.851 \pm 0.013$	0.00
	NeuralTextures	$76.83 \pm 2.21$	$0.845 \pm 0.026$	$0.781 \pm 0.024$	$0.824 \pm 0.013$	$0.742 \pm 0.036$	0.00
*Logistic*							
	DeepFakes	$80.00 \pm 0.65$	$0.880 \pm 0.010$	$0.799 \pm 0.012$	$0.802 \pm 0.009$	$0.797 \pm 0.032$	0.25
	Face2Face	$80.00 \pm 0.65$	$0.880 \pm 0.010$	$0.799 \pm 0.012$	$0.802 \pm 0.009$	$0.797 \pm 0.032$	0.25
	FaceShifter	$77.33 \pm 0.72$	$0.850 \pm 0.006$	$0.768 \pm 0.008$	$0.786 \pm 0.010$	$0.752 \pm 0.013$	0.86
	FaceSwap	$73.59 \pm 3.21$	$0.803 \pm 0.027$	$0.770 \pm 0.023$	$0.750 \pm 0.039$	$0.792 \pm 0.006$	0.84
	NeuralTextures	$72.66 \pm 0.42$	$0.795 \pm 0.011$	$0.761 \pm 0.005$	$0.741 \pm 0.001$	$0.783 \pm 0.010$	0.90
*MLP*							
	DeepFakes	$89.29 \pm 1.13$	$0.956 \pm 0.004$	$0.893 \pm 0.011$	$0.892 \pm 0.016$	$0.894 \pm 0.017$	13.27
	Face2Face	$89.29 \pm 1.13$	$0.956 \pm 0.004$	$0.893 \pm 0.011$	$0.892 \pm 0.016$	$0.894 \pm 0.017$	13.27
	FaceShifter	$85.48 \pm 1.76$	$0.928 \pm 0.014$	$0.854 \pm 0.016$	$0.858 \pm 0.024$	$0.850 \pm 0.010$	40.06
	FaceSwap	$76.86 \pm 1.27$	$0.844 \pm 0.015$	$0.794 \pm 0.005$	$0.789 \pm 0.028$	$0.799 \pm 0.019$	11.16
	NeuralTextures	$77.32 \pm 1.49$	$0.849 \pm 0.018$	$0.795 \pm 0.011$	$0.801 \pm 0.032$	$0.789 \pm 0.031$	35.82
*RandomForest*							
	DeepFakes	$86.58 \pm 0.54$	$0.941 \pm 0.004$	$0.863 \pm 0.006$	$0.880 \pm 0.013$	$0.847 \pm 0.019$	9.24
	Face2Face	$86.58 \pm 0.54$	$0.941 \pm 0.004$	$0.863 \pm 0.006$	$0.880 \pm 0.013$	$0.847 \pm 0.019$	9.24
	FaceShifter	$83.86 \pm 0.57$	$0.921 \pm 0.007$	$0.835 \pm 0.004$	$0.853 \pm 0.012$	$0.818 \pm 0.003$	11.56
	FaceSwap	$76.61 \pm 2.81$	$0.847 \pm 0.034$	$0.798 \pm 0.023$	$0.770 \pm 0.033$	$0.829 \pm 0.036$	3.03
	NeuralTextures	$77.92 \pm 1.31$	$0.861 \pm 0.014$	$0.809 \pm 0.013$	$0.781 \pm 0.012$	$0.838 \pm 0.026$	12.22
*SVM-RBF*							
	DeepFakes	$90.69 \pm 0.74$	$0.967 \pm 0.002$	$0.906 \pm 0.007$	$0.916 \pm 0.009$	$0.896 \pm 0.010$	29.80
	Face2Face	$90.69 \pm 0.74$	$0.967 \pm 0.002$	$0.906 \pm 0.007$	$0.916 \pm 0.009$	$0.896 \pm 0.010$	29.80
	FaceShifter	$87.62 \pm 0.98$	$0.944 \pm 0.007$	$0.874 \pm 0.010$	$0.894 \pm 0.013$	$0.854 \pm 0.008$	74.63
	FaceSwap	$79.67 \pm 3.51$	$0.873 \pm 0.035$	$0.824 \pm 0.027$	$0.797 \pm 0.039$	$0.853 \pm 0.019$	8.81
	NeuralTextures	$79.71 \pm 1.07$	$0.875 \pm 0.017$	$0.821 \pm 0.012$	$0.805 \pm 0.002$	$0.838 \pm 0.023$	79.55
*XGBoost*							
	DeepFakes	$88.62 \pm 1.19$	$0.957 \pm 0.007$	$0.885 \pm 0.011$	$0.894 \pm 0.019$	$0.877 \pm 0.012$	0.89
	Face2Face	$88.62 \pm 1.19$	$0.957 \pm 0.007$	$0.885 \pm 0.011$	$0.894 \pm 0.019$	$0.877 \pm 0.012$	0.89
	FaceShifter	$85.88 \pm 0.23$	$0.936 \pm 0.004$	$0.857 \pm 0.002$	$0.868 \pm 0.008$	$0.847 \pm 0.005$	0.44
	FaceSwap	$78.17 \pm 2.45$	$0.859 \pm 0.016$	$0.810 \pm 0.019$	$0.789 \pm 0.032$	$0.831 \pm 0.025$	0.18
	NeuralTextures	$78.55 \pm 2.90$	$0.867 \pm 0.020$	$0.810 \pm 0.024$	$0.799 \pm 0.031$	$0.822 \pm 0.017$	0.33
**Average**		**80.80**	**0.873**	**0.819**	−	−	−

Method: kbest+lasso; Values shown as Mean ± 95% Confidence Interval; FaceForensics++ C23 compression level.

**Table 26:** Performance Results for rfe Feature Selection (FF++ c23 dataset).

**Classifier**	**Manipulation**	**Acc (%)**	**AUC**	**F1**	**Precision**	**Recall**	**Time (s)**
*AdaBoost*							
	DeepFakes	$76.92 \pm 2.26$	$0.849 \pm 0.020$	$0.770 \pm 0.016$	$0.767 \pm 0.039$	$0.775 \pm 0.019$	4.57
	Face2Face	$76.92 \pm 2.26$	$0.849 \pm 0.020$	$0.770 \pm 0.016$	$0.767 \pm 0.039$	$0.775 \pm 0.019$	4.57
	FaceShifter	$74.99 \pm 1.71$	$0.828 \pm 0.025$	$0.744 \pm 0.020$	$0.763 \pm 0.022$	$0.726 \pm 0.035$	5.44
	FaceSwap	$73.13 \pm 3.49$	$0.803 \pm 0.041$	$0.765 \pm 0.039$	$0.747 \pm 0.021$	$0.784 \pm 0.069$	1.75
	NeuralTextures	$67.92 \pm 2.59$	$0.742 \pm 0.040$	$0.722 \pm 0.025$	$0.697 \pm 0.038$	$0.750 \pm 0.068$	4.57
*DecisionTree*							
	DeepFakes	$71.09 \pm 1.51$	$0.711 \pm 0.015$	$0.710 \pm 0.015$	$0.712 \pm 0.016$	$0.708 \pm 0.014$	0.99
	Face2Face	$71.09 \pm 1.51$	$0.711 \pm 0.015$	$0.710 \pm 0.015$	$0.712 \pm 0.016$	$0.708 \pm 0.014$	0.99
	FaceShifter	$69.27 \pm 3.04$	$0.693 \pm 0.030$	$0.694 \pm 0.026$	$0.692 \pm 0.035$	$0.695 \pm 0.017$	1.22
	FaceSwap	$65.36 \pm 2.74$	$0.650 \pm 0.029$	$0.687 \pm 0.025$	$0.693 \pm 0.030$	$0.681 \pm 0.036$	0.33
	NeuralTextures	$60.73 \pm 7.05$	$0.602 \pm 0.073$	$0.648 \pm 0.060$	$0.646 \pm 0.066$	$0.650 \pm 0.054$	0.98
*ExtraTrees*							
	DeepFakes	$86.87 \pm 1.98$	$0.944 \pm 0.018$	$0.866 \pm 0.021$	$0.881 \pm 0.018$	$0.852 \pm 0.027$	1.67
	Face2Face	$86.87 \pm 1.98$	$0.944 \pm 0.018$	$0.866 \pm 0.021$	$0.881 \pm 0.018$	$0.852 \pm 0.027$	1.67
	FaceShifter	$83.48 \pm 1.75$	$0.915 \pm 0.020$	$0.833 \pm 0.019$	$0.844 \pm 0.017$	$0.822 \pm 0.025$	2.38
	FaceSwap	$78.81 \pm 3.17$	$0.872 \pm 0.026$	$0.821 \pm 0.025$	$0.778 \pm 0.032$	$0.868 \pm 0.019$	0.77
	NeuralTextures	$72.91 \pm 2.81$	$0.804 \pm 0.033$	$0.774 \pm 0.016$	$0.723 \pm 0.038$	$0.834 \pm 0.026$	2.44
*GradientBoosting*							
	DeepFakes	$80.96 \pm 1.59$	$0.893 \pm 0.018$	$0.809 \pm 0.014$	$0.811 \pm 0.023$	$0.808 \pm 0.015$	24.65
	Face2Face	$80.96 \pm 1.59$	$0.893 \pm 0.018$	$0.809 \pm 0.014$	$0.811 \pm 0.023$	$0.808 \pm 0.015$	24.65
	FaceShifter	$79.01 \pm 1.91$	$0.873 \pm 0.021$	$0.785 \pm 0.019$	$0.806 \pm 0.026$	$0.765 \pm 0.023$	29.25
	FaceSwap	$76.78 \pm 0.98$	$0.848 \pm 0.016$	$0.801 \pm 0.006$	$0.768 \pm 0.020$	$0.836 \pm 0.024$	9.14
	NeuralTextures	$70.87 \pm 3.85$	$0.780 \pm 0.041$	$0.753 \pm 0.028$	$0.714 \pm 0.046$	$0.796 \pm 0.048$	24.39
*KNN*							
	DeepFakes	$90.21 \pm 3.21$	$0.961 \pm 0.020$	$0.901 \pm 0.032$	$0.915 \pm 0.041$	$0.886 \pm 0.025$	0.00
	Face2Face	$90.21 \pm 3.21$	$0.961 \pm 0.020$	$0.901 \pm 0.032$	$0.915 \pm 0.041$	$0.886 \pm 0.025$	0.00
	FaceShifter	$85.17 \pm 2.16$	$0.928 \pm 0.015$	$0.844 \pm 0.025$	$0.889 \pm 0.012$	$0.804 \pm 0.037$	0.00
	FaceSwap	$78.89 \pm 3.20$	$0.856 \pm 0.048$	$0.812 \pm 0.028$	$0.809 \pm 0.032$	$0.814 \pm 0.028$	0.00
	NeuralTextures	$65.82 \pm 2.05$	$0.713 \pm 0.023$	$0.679 \pm 0.027$	$0.712 \pm 0.009$	$0.648 \pm 0.041$	0.00
*Logistic*							
	DeepFakes	$81.00 \pm 2.45$	$0.891 \pm 0.020$	$0.810 \pm 0.025$	$0.809 \pm 0.024$	$0.811 \pm 0.028$	0.13
	Face2Face	$81.00 \pm 2.45$	$0.891 \pm 0.020$	$0.810 \pm 0.025$	$0.809 \pm 0.024$	$0.811 \pm 0.028$	0.13
	FaceShifter	$79.60 \pm 1.44$	$0.875 \pm 0.021$	$0.792 \pm 0.015$	$0.809 \pm 0.016$	$0.775 \pm 0.018$	0.58
	FaceSwap	$76.52 \pm 1.87$	$0.843 \pm 0.007$	$0.793 \pm 0.011$	$0.781 \pm 0.031$	$0.806 \pm 0.014$	0.67
	NeuralTextures	$71.59 \pm 2.24$	$0.781 \pm 0.025$	$0.752 \pm 0.020$	$0.731 \pm 0.019$	$0.774 \pm 0.021$	0.54
*MLP*							
	DeepFakes	$84.65 \pm 3.21$	$0.920 \pm 0.026$	$0.848 \pm 0.032$	$0.840 \pm 0.029$	$0.855 \pm 0.036$	13.43
	Face2Face	$84.65 \pm 3.21$	$0.920 \pm 0.026$	$0.848 \pm 0.032$	$0.840 \pm 0.029$	$0.855 \pm 0.036$	13.43
	FaceShifter	$82.10 \pm 4.03$	$0.896 \pm 0.038$	$0.822 \pm 0.040$	$0.818 \pm 0.041$	$0.826 \pm 0.040$	32.85
	FaceSwap	$78.05 \pm 1.28$	$0.858 \pm 0.006$	$0.807 \pm 0.008$	$0.794 \pm 0.023$	$0.819 \pm 0.016$	9.43
	NeuralTextures	$70.81 \pm 3.33$	$0.769 \pm 0.034$	$0.739 \pm 0.029$	$0.735 \pm 0.034$	$0.744 \pm 0.035$	35.81
*RandomForest*							
	DeepFakes	$85.33 \pm 0.99$	$0.931 \pm 0.020$	$0.851 \pm 0.010$	$0.862 \pm 0.013$	$0.841 \pm 0.012$	9.04
	Face2Face	$85.33 \pm 0.99$	$0.931 \pm 0.020$	$0.851 \pm 0.010$	$0.862 \pm 0.013$	$0.841 \pm 0.012$	9.04
	FaceShifter	$82.36 \pm 1.26$	$0.903 \pm 0.018$	$0.821 \pm 0.012$	$0.833 \pm 0.020$	$0.810 \pm 0.015$	11.22
	FaceSwap	$77.56 \pm 2.15$	$0.859 \pm 0.021$	$0.810 \pm 0.017$	$0.769 \pm 0.022$	$0.856 \pm 0.021$	3.18
	NeuralTextures	$72.35 \pm 2.91$	$0.797 \pm 0.039$	$0.768 \pm 0.013$	$0.721 \pm 0.044$	$0.822 \pm 0.028$	9.73
*SVM-RBF*							
	DeepFakes	$86.98 \pm 2.54$	$0.941 \pm 0.019$	$0.869 \pm 0.025$	$0.875 \pm 0.027$	$0.863 \pm 0.023$	28.43
	Face2Face	$86.98 \pm 2.54$	$0.941 \pm 0.019$	$0.869 \pm 0.025$	$0.875 \pm 0.027$	$0.863 \pm 0.023$	28.43
	FaceShifter	$84.69 \pm 3.42$	$0.921 \pm 0.026$	$0.845 \pm 0.034$	$0.854 \pm 0.036$	$0.837 \pm 0.033$	71.39
	FaceSwap	$80.16 \pm 0.48$	$0.884 \pm 0.011$	$0.826 \pm 0.008$	$0.809 \pm 0.017$	$0.844 \pm 0.032$	7.71
	NeuralTextures	$73.98 \pm 2.49$	$0.813 \pm 0.030$	$0.776 \pm 0.019$	$0.746 \pm 0.025$	$0.808 \pm 0.014$	83.84
*XGBoost*							
	DeepFakes	$86.41 \pm 2.42$	$0.939 \pm 0.020$	$0.864 \pm 0.022$	$0.864 \pm 0.034$	$0.864 \pm 0.010$	1.22
	Face2Face	$86.41 \pm 2.42$	$0.939 \pm 0.020$	$0.864 \pm 0.022$	$0.864 \pm 0.034$	$0.864 \pm 0.010$	1.22
	FaceShifter	$84.35 \pm 2.78$	$0.921 \pm 0.023$	$0.842 \pm 0.027$	$0.850 \pm 0.037$	$0.834 \pm 0.025$	0.46
	FaceSwap	$80.60 \pm 1.11$	$0.884 \pm 0.008$	$0.830 \pm 0.009$	$0.812 \pm 0.027$	$0.850 \pm 0.035$	0.31
	NeuralTextures	$73.36 \pm 3.62$	$0.808 \pm 0.034$	$0.767 \pm 0.028$	$0.748 \pm 0.038$	$0.786 \pm 0.021$	0.43
**Average**		**78.64**	**0.854**	**0.799**	−	−	−

Method: rfe; Values shown as Mean ± 95% Confidence Interval; FaceForensics++ C23 compression level.

**Table 27:** Performance Results for rfe+lasso Feature Selection (FF++ c23 dataset).

**Classifier**	**Manipulation**	**Acc (%)**	**AUC**	**F1**	**Precision**	**Recall**	**Time (s)**
*AdaBoost*							
	DeepFakes	$76.92 \pm 2.26$	$0.849 \pm 0.020$	$0.770 \pm 0.016$	$0.767 \pm 0.039$	$0.775 \pm 0.019$	4.56
	Face2Face	$76.92 \pm 2.26$	$0.849 \pm 0.020$	$0.770 \pm 0.016$	$0.767 \pm 0.039$	$0.775 \pm 0.019$	4.56
	FaceShifter	$74.99 \pm 1.71$	$0.828 \pm 0.025$	$0.744 \pm 0.020$	$0.763 \pm 0.022$	$0.726 \pm 0.035$	5.45
	FaceSwap	$73.13 \pm 3.49$	$0.803 \pm 0.041$	$0.765 \pm 0.039$	$0.747 \pm 0.021$	$0.784 \pm 0.069$	1.75
	NeuralTextures	$67.92 \pm 2.59$	$0.742 \pm 0.040$	$0.722 \pm 0.025$	$0.697 \pm 0.038$	$0.750 \pm 0.068$	4.59
*DecisionTree*							
	DeepFakes	$71.09 \pm 1.51$	$0.711 \pm 0.015$	$0.710 \pm 0.015$	$0.712 \pm 0.016$	$0.708 \pm 0.014$	0.97
	Face2Face	$71.09 \pm 1.51$	$0.711 \pm 0.015$	$0.710 \pm 0.015$	$0.712 \pm 0.016$	$0.708 \pm 0.014$	0.97
	FaceShifter	$69.27 \pm 3.04$	$0.693 \pm 0.030$	$0.694 \pm 0.026$	$0.692 \pm 0.035$	$0.695 \pm 0.017$	1.22
	FaceSwap	$65.36 \pm 2.74$	$0.650 \pm 0.029$	$0.687 \pm 0.025$	$0.693 \pm 0.030$	$0.681 \pm 0.036$	0.33
	NeuralTextures	$60.73 \pm 7.05$	$0.602 \pm 0.073$	$0.648 \pm 0.060$	$0.646 \pm 0.066$	$0.650 \pm 0.054$	0.98
*ExtraTrees*							
	DeepFakes	$86.87 \pm 1.98$	$0.944 \pm 0.018$	$0.866 \pm 0.021$	$0.881 \pm 0.018$	$0.852 \pm 0.027$	1.65
	Face2Face	$86.87 \pm 1.98$	$0.944 \pm 0.018$	$0.866 \pm 0.021$	$0.881 \pm 0.018$	$0.852 \pm 0.027$	1.65
	FaceShifter	$83.48 \pm 1.75$	$0.915 \pm 0.020$	$0.833 \pm 0.019$	$0.844 \pm 0.017$	$0.822 \pm 0.025$	2.38
	FaceSwap	$78.81 \pm 3.17$	$0.872 \pm 0.026$	$0.821 \pm 0.025$	$0.778 \pm 0.032$	$0.868 \pm 0.019$	0.77
	NeuralTextures	$72.91 \pm 2.81$	$0.804 \pm 0.033$	$0.774 \pm 0.016$	$0.723 \pm 0.038$	$0.834 \pm 0.026$	2.45
*GradientBoosting*							
	DeepFakes	$80.96 \pm 1.59$	$0.893 \pm 0.018$	$0.809 \pm 0.014$	$0.811 \pm 0.023$	$0.808 \pm 0.015$	24.58
	Face2Face	$80.96 \pm 1.59$	$0.893 \pm 0.018$	$0.809 \pm 0.014$	$0.811 \pm 0.023$	$0.808 \pm 0.015$	24.58
	FaceShifter	$79.01 \pm 1.91$	$0.873 \pm 0.021$	$0.785 \pm 0.019$	$0.806 \pm 0.026$	$0.765 \pm 0.023$	29.24
	FaceSwap	$76.78 \pm 0.98$	$0.848 \pm 0.016$	$0.801 \pm 0.006$	$0.768 \pm 0.020$	$0.836 \pm 0.024$	9.14
	NeuralTextures	$70.87 \pm 3.85$	$0.780 \pm 0.041$	$0.753 \pm 0.028$	$0.714 \pm 0.046$	$0.796 \pm 0.048$	24.37
*KNN*							
	DeepFakes	$90.21 \pm 3.21$	$0.961 \pm 0.020$	$0.901 \pm 0.032$	$0.915 \pm 0.041$	$0.886 \pm 0.025$	0.00
	Face2Face	$90.21 \pm 3.21$	$0.961 \pm 0.020$	$0.901 \pm 0.032$	$0.915 \pm 0.041$	$0.886 \pm 0.025$	0.00
	FaceShifter	$85.17 \pm 2.16$	$0.928 \pm 0.015$	$0.844 \pm 0.025$	$0.889 \pm 0.012$	$0.804 \pm 0.037$	0.00
	FaceSwap	$78.89 \pm 3.20$	$0.856 \pm 0.048$	$0.812 \pm 0.028$	$0.809 \pm 0.032$	$0.814 \pm 0.028$	0.00
	NeuralTextures	$65.82 \pm 2.05$	$0.713 \pm 0.023$	$0.679 \pm 0.027$	$0.712 \pm 0.009$	$0.648 \pm 0.041$	0.00
*Logistic*							
	DeepFakes	$81.00 \pm 2.45$	$0.891 \pm 0.020$	$0.810 \pm 0.025$	$0.809 \pm 0.024$	$0.811 \pm 0.028$	0.11
	Face2Face	$81.00 \pm 2.45$	$0.891 \pm 0.020$	$0.810 \pm 0.025$	$0.809 \pm 0.024$	$0.811 \pm 0.028$	0.11
	FaceShifter	$79.60 \pm 1.44$	$0.875 \pm 0.021$	$0.792 \pm 0.015$	$0.809 \pm 0.016$	$0.775 \pm 0.018$	0.66
	FaceSwap	$76.52 \pm 1.87$	$0.843 \pm 0.007$	$0.793 \pm 0.011$	$0.781 \pm 0.031$	$0.806 \pm 0.014$	0.65
	NeuralTextures	$71.59 \pm 2.24$	$0.781 \pm 0.025$	$0.752 \pm 0.020$	$0.731 \pm 0.019$	$0.774 \pm 0.021$	0.52
*MLP*							
	DeepFakes	$84.65 \pm 3.21$	$0.920 \pm 0.026$	$0.848 \pm 0.032$	$0.840 \pm 0.029$	$0.855 \pm 0.036$	13.58
	Face2Face	$84.65 \pm 3.21$	$0.920 \pm 0.026$	$0.848 \pm 0.032$	$0.840 \pm 0.029$	$0.855 \pm 0.036$	13.58
	FaceShifter	$81.86 \pm 4.38$	$0.894 \pm 0.034$	$0.820 \pm 0.041$	$0.816 \pm 0.054$	$0.823 \pm 0.033$	40.83
	FaceSwap	$77.79 \pm 0.42$	$0.857 \pm 0.003$	$0.804 \pm 0.008$	$0.792 \pm 0.027$	$0.816 \pm 0.043$	10.66
	NeuralTextures	$70.51 \pm 2.42$	$0.766 \pm 0.031$	$0.735 \pm 0.014$	$0.736 \pm 0.043$	$0.735 \pm 0.037$	35.52
*RandomForest*							
	DeepFakes	$85.33 \pm 0.99$	$0.931 \pm 0.020$	$0.851 \pm 0.010$	$0.862 \pm 0.013$	$0.841 \pm 0.012$	8.99
	Face2Face	$85.33 \pm 0.99$	$0.931 \pm 0.020$	$0.851 \pm 0.010$	$0.862 \pm 0.013$	$0.841 \pm 0.012$	8.99
	FaceShifter	$82.36 \pm 1.26$	$0.903 \pm 0.018$	$0.821 \pm 0.012$	$0.833 \pm 0.020$	$0.810 \pm 0.015$	11.24
	FaceSwap	$77.56 \pm 2.15$	$0.859 \pm 0.021$	$0.810 \pm 0.017$	$0.769 \pm 0.022$	$0.856 \pm 0.021$	3.18
	NeuralTextures	$72.35 \pm 2.91$	$0.797 \pm 0.039$	$0.768 \pm 0.013$	$0.721 \pm 0.044$	$0.822 \pm 0.028$	9.69
*SVM-RBF*							
	DeepFakes	$86.98 \pm 2.54$	$0.941 \pm 0.019$	$0.869 \pm 0.025$	$0.875 \pm 0.027$	$0.863 \pm 0.023$	28.77
	Face2Face	$86.98 \pm 2.54$	$0.941 \pm 0.019$	$0.869 \pm 0.025$	$0.875 \pm 0.027$	$0.863 \pm 0.023$	28.77
	FaceShifter	$84.69 \pm 3.42$	$0.921 \pm 0.026$	$0.845 \pm 0.034$	$0.854 \pm 0.036$	$0.837 \pm 0.033$	72.62
	FaceSwap	$80.16 \pm 0.48$	$0.884 \pm 0.011$	$0.826 \pm 0.008$	$0.809 \pm 0.017$	$0.844 \pm 0.032$	7.59
	NeuralTextures	$73.98 \pm 2.49$	$0.813 \pm 0.030$	$0.776 \pm 0.019$	$0.746 \pm 0.025$	$0.808 \pm 0.014$	84.95
*XGBoost*							
	DeepFakes	$86.41 \pm 2.42$	$0.939 \pm 0.020$	$0.864 \pm 0.022$	$0.864 \pm 0.034$	$0.864 \pm 0.010$	1.30
	Face2Face	$86.41 \pm 2.42$	$0.939 \pm 0.020$	$0.864 \pm 0.022$	$0.864 \pm 0.034$	$0.864 \pm 0.010$	1.30
	FaceShifter	$84.35 \pm 2.78$	$0.921 \pm 0.023$	$0.842 \pm 0.027$	$0.850 \pm 0.037$	$0.834 \pm 0.025$	0.41
	FaceSwap	$80.60 \pm 1.11$	$0.884 \pm 0.008$	$0.830 \pm 0.009$	$0.812 \pm 0.027$	$0.850 \pm 0.035$	0.39
	NeuralTextures	$73.36 \pm 3.62$	$0.808 \pm 0.034$	$0.767 \pm 0.028$	$0.748 \pm 0.038$	$0.786 \pm 0.021$	0.31
**Average**		**78.62**	**0.853**	**0.799**	−	−	−

Method: rfe+lasso. Values shown as Mean ± 95% Confidence Interval. FaceForensics++ C23 compression level.

**Table 28:** Performance Results for ga Feature Selection (Celeb DF dataset).

**Classifier**	**Manipulation**	**Acc (%)**	**AUC**	**F1**	**Precision**	**Recall**	**Time (s)**
*AdaBoost*							
	CelebDF	$73.20 \pm 1.97$	$0.811 \pm 0.020$	$0.729 \pm 0.020$	$0.737 \pm 0.027$	$0.722 \pm 0.031$	168.27
	CelebDFC40	$65.86 \pm 1.45$	$0.718 \pm 0.015$	$0.639 \pm 0.030$	$0.678 \pm 0.004$	$0.604 \pm 0.053$	176.94
*DecisionTree*							
	CelebDF	$71.93 \pm 0.79$	$0.720 \pm 0.009$	$0.726 \pm 0.006$	$0.710 \pm 0.014$	$0.742 \pm 0.015$	56.08
	CelebDFC40	$48.40 \pm 0.00$	$0.482 \pm 0.000$	$0.487 \pm 0.000$	$0.484 \pm 0.000$	$0.489 \pm 0.000$	49.73
*ExtraTrees*							
	CelebDF	$81.69 \pm 0.13$	$0.900 \pm 0.007$	$0.816 \pm 0.001$	$0.819 \pm 0.010$	$0.813 \pm 0.012$	20.97
	CelebDFC40	$48.40 \pm 0.00$	$0.482 \pm 0.000$	$0.487 \pm 0.000$	$0.484 \pm 0.000$	$0.489 \pm 0.000$	21.65
*GradientBoosting*							
	CelebDF	$78.91 \pm 1.57$	$0.869 \pm 0.004$	$0.787 \pm 0.012$	$0.796 \pm 0.028$	$0.778 \pm 0.011$	914.19
	CelebDFC40	$68.87 \pm 2.23$	$0.753 \pm 0.010$	$0.670 \pm 0.027$	$0.712 \pm 0.021$	$0.634 \pm 0.031$	954.98
*KNN*							
	CelebDF	$77.20 \pm 0.60$	$0.839 \pm 0.002$	$0.762 \pm 0.008$	$0.796 \pm 0.002$	$0.731 \pm 0.013$	0.07
	CelebDFC40	$49.20 \pm 0.43$	$0.491 \pm 0.006$	$0.405 \pm 0.006$	$0.489 \pm 0.006$	$0.346 \pm 0.007$	0.07
*Logistic*							
	CelebDF	$82.78 \pm 1.64$	$0.905 \pm 0.013$	$0.831 \pm 0.015$	$0.816 \pm 0.021$	$0.847 \pm 0.017$	6.03
	CelebDFC40	$68.68 \pm 1.39$	$0.751 \pm 0.005$	$0.674 \pm 0.015$	$0.702 \pm 0.015$	$0.648 \pm 0.015$	4.97
*MLP*							
	CelebDF	$88.71 \pm 0.64$	$0.945 \pm 0.010$	$0.889 \pm 0.006$	$0.875 \pm 0.013$	$0.903 \pm 0.011$	31.54
	CelebDFC40	$48.36 \pm 0.99$	$0.516 \pm 0.016$	$0.484 \pm 0.003$	$0.484 \pm 0.009$	$0.485 \pm 0.013$	34.02
*RandomForest*							
	CelebDF	$81.36 \pm 1.89$	$0.895 \pm 0.003$	$0.814 \pm 0.021$	$0.810 \pm 0.011$	$0.818 \pm 0.033$	91.63
	CelebDFC40	$48.40 \pm 0.00$	$0.569 \pm 0.011$	$0.487 \pm 0.000$	$0.484 \pm 0.000$	$0.489 \pm 0.000$	93.36
*SVM-RBF*							
	CelebDF	$87.59 \pm 0.47$	$0.939 \pm 0.006$	$0.877 \pm 0.005$	$0.869 \pm 0.005$	$0.885 \pm 0.004$	2798.83
	CelebDFC40	$63.83 \pm 0.93$	$0.708 \pm 0.002$	$0.601 \pm 0.011$	$0.670 \pm 0.011$	$0.545 \pm 0.011$	4003.71
*XGBoost*							
	CelebDF	$85.46 \pm 0.62$	$0.929 \pm 0.007$	$0.856 \pm 0.007$	$0.847 \pm 0.004$	$0.865 \pm 0.009$	8.13
	CelebDFC40	$50.92 \pm 0.55$	$0.574 \pm 0.008$	$0.498 \pm 0.002$	$0.510 \pm 0.006$	$0.487 \pm 0.002$	7.26
**Average**		**68.49**	**0.740**	**0.676**	−	−	−

Method: ga.

Values shown as Mean ± 95% Confidence Interval.

**Table 29:** Performance Results for ga+lasso Feature Selection (Celeb DF dataset).

**Classifier**	**Manipulation**	**Acc (%)**	**AUC**	**F1**	**Precision**	**Recall**	**Time (s)**
*AdaBoost*							
	CelebDF	$73.62 \pm 1.43$	$0.811 \pm 0.021$	$0.735 \pm 0.024$	$0.738 \pm 0.017$	$0.732 \pm 0.057$	117.50
	CelebDFC40	$64.97 \pm 2.88$	$0.708 \pm 0.030$	$0.633 \pm 0.035$	$0.665 \pm 0.027$	$0.604 \pm 0.043$	128.64
*DecisionTree*							
	CelebDF	$71.21 \pm 0.17$	$0.713 \pm 0.002$	$0.717 \pm 0.005$	$0.706 \pm 0.005$	$0.728 \pm 0.015$	36.48
	CelebDFC40	$48.40 \pm 0.00$	$0.482 \pm 0.000$	$0.487 \pm 0.000$	$0.484 \pm 0.000$	$0.489 \pm 0.000$	36.11
*ExtraTrees*							
	CelebDF	$82.11 \pm 1.29$	$0.902 \pm 0.006$	$0.821 \pm 0.010$	$0.822 \pm 0.022$	$0.819 \pm 0.006$	17.14
	CelebDFC40	$48.40 \pm 0.00$	$0.482 \pm 0.000$	$0.487 \pm 0.000$	$0.484 \pm 0.000$	$0.489 \pm 0.000$	18.26
*GradientBoosting*							
	CelebDF	$78.94 \pm 1.91$	$0.869 \pm 0.001$	$0.786 \pm 0.019$	$0.798 \pm 0.021$	$0.774 \pm 0.019$	637.09
	CelebDFC40	$68.98 \pm 2.28$	$0.755 \pm 0.015$	$0.672 \pm 0.020$	$0.713 \pm 0.032$	$0.636 \pm 0.010$	701.34
*KNN*							
	CelebDF	$77.94 \pm 1.00$	$0.844 \pm 0.007$	$0.771 \pm 0.008$	$0.801 \pm 0.017$	$0.743 \pm 0.002$	0.05
	CelebDFC40	$49.63 \pm 1.36$	$0.495 \pm 0.016$	$0.412 \pm 0.022$	$0.495 \pm 0.019$	$0.354 \pm 0.023$	0.05
*Logistic*							
	CelebDF	$82.84 \pm 1.41$	$0.904 \pm 0.014$	$0.831 \pm 0.015$	$0.817 \pm 0.011$	$0.846 \pm 0.020$	3.76
	CelebDFC40	$68.61 \pm 0.48$	$0.751 \pm 0.003$	$0.674 \pm 0.005$	$0.701 \pm 0.005$	$0.648 \pm 0.006$	4.19
*MLP*							
	CelebDF	$88.20 \pm 0.08$	$0.945 \pm 0.003$	$0.884 \pm 0.001$	$0.869 \pm 0.004$	$0.900 \pm 0.004$	22.15
	CelebDFC40	$48.24 \pm 0.48$	$0.519 \pm 0.022$	$0.486 \pm 0.003$	$0.483 \pm 0.005$	$0.490 \pm 0.003$	34.27
*RandomForest*							
	CelebDF	$82.01 \pm 1.68$	$0.899 \pm 0.004$	$0.821 \pm 0.015$	$0.815 \pm 0.022$	$0.828 \pm 0.008$	76.39
	CelebDFC40	$48.40 \pm 0.00$	$0.568 \pm 0.005$	$0.487 \pm 0.000$	$0.484 \pm 0.000$	$0.489 \pm 0.000$	78.87
*SVM-RBF*							
	CelebDF	$87.34 \pm 1.04$	$0.940 \pm 0.006$	$0.875 \pm 0.009$	$0.867 \pm 0.015$	$0.882 \pm 0.005$	1967.35
	CelebDFC40	$64.09 \pm 0.92$	$0.707 \pm 0.005$	$0.606 \pm 0.008$	$0.672 \pm 0.013$	$0.551 \pm 0.006$	2995.40
*XGBoost*							
	CelebDF	$85.62 \pm 1.40$	$0.931 \pm 0.007$	$0.858 \pm 0.013$	$0.849 \pm 0.020$	$0.867 \pm 0.005$	5.01
	CelebDFC40	$51.41 \pm 0.91$	$0.578 \pm 0.007$	$0.499 \pm 0.012$	$0.515 \pm 0.010$	$0.483 \pm 0.014$	4.92
**Average**		**68.55**	**0.740**	**0.677**	−	−	−

Method: ga+lasso.

Values shown as Mean ± 95% Confidence Interval.

**Table 30:** Performance Results for extratrees Feature Selection (Celeb DF dataset).

**Classifier**	**Manipulation**	**Acc (%)**	**AUC**	**F1**	**Precision**	**Recall**	**Time (s)**
*AdaBoost*							
	CelebDF	$71.67 \pm 0.00$	$0.794 \pm 0.000$	$0.715 \pm 0.000$	$0.719 \pm 0.000$	$0.711 \pm 0.000$	10.50
	CelebDFC40	$67.10 \pm 0.00$	$0.722 \pm 0.000$	$0.656 \pm 0.000$	$0.687 \pm 0.000$	$0.628 \pm 0.000$	10.37
*DecisionTree*							
	CelebDF	$73.53 \pm 0.00$	$0.736 \pm 0.000$	$0.741 \pm 0.000$	$0.726 \pm 0.000$	$0.755 \pm 0.000$	2.91
	CelebDFC40	$48.40 \pm 0.00$	$0.482 \pm 0.000$	$0.487 \pm 0.000$	$0.484 \pm 0.000$	$0.489 \pm 0.000$	2.68
*ExtraTrees*							
	CelebDF	$82.93 \pm 0.00$	$0.907 \pm 0.000$	$0.829 \pm 0.000$	$0.830 \pm 0.000$	$0.828 \pm 0.000$	3.66
	CelebDFC40	$48.40 \pm 0.00$	$0.482 \pm 0.000$	$0.487 \pm 0.000$	$0.484 \pm 0.000$	$0.489 \pm 0.000$	4.02
*GradientBoosting*							
	CelebDF	$77.77 \pm 0.00$	$0.850 \pm 0.000$	$0.774 \pm 0.000$	$0.788 \pm 0.000$	$0.759 \pm 0.000$	56.87
	CelebDFC40	$68.37 \pm 0.00$	$0.752 \pm 0.000$	$0.664 \pm 0.000$	$0.709 \pm 0.000$	$0.624 \pm 0.000$	56.17
*KNN*							
	CelebDF	$84.10 \pm 0.00$	$0.903 \pm 0.000$	$0.837 \pm 0.000$	$0.858 \pm 0.000$	$0.817 \pm 0.000$	0.00
	CelebDFC40	$54.90 \pm 0.00$	$0.574 \pm 0.000$	$0.495 \pm 0.000$	$0.562 \pm 0.000$	$0.442 \pm 0.000$	0.00
*Logistic*							
	CelebDF	$75.33 \pm 0.00$	$0.822 \pm 0.000$	$0.748 \pm 0.000$	$0.765 \pm 0.000$	$0.732 \pm 0.000$	0.87
	CelebDFC40	$68.50 \pm 0.00$	$0.751 \pm 0.000$	$0.667 \pm 0.000$	$0.707 \pm 0.000$	$0.631 \pm 0.000$	0.82
*MLP*							
	CelebDF	$82.70 \pm 1.23$	$0.907 \pm 0.007$	$0.830 \pm 0.011$	$0.816 \pm 0.019$	$0.844 \pm 0.016$	38.93
	CelebDFC40	$57.02 \pm 3.12$	$0.602 \pm 0.057$	$0.534 \pm 0.057$	$0.583 \pm 0.028$	$0.493 \pm 0.077$	44.27
*RandomForest*							
	CelebDF	$82.73 \pm 0.00$	$0.900 \pm 0.000$	$0.829 \pm 0.000$	$0.822 \pm 0.000$	$0.836 \pm 0.000$	24.28
	CelebDFC40	$48.40 \pm 0.00$	$0.580 \pm 0.000$	$0.487 \pm 0.000$	$0.484 \pm 0.000$	$0.489 \pm 0.000$	23.63
*SVM-RBF*							
	CelebDF	$85.03 \pm 0.00$	$0.919 \pm 0.000$	$0.847 \pm 0.000$	$0.868 \pm 0.000$	$0.827 \pm 0.000$	179.34
	CelebDFC40	$67.33 \pm 0.00$	$0.736 \pm 0.000$	$0.633 \pm 0.000$	$0.723 \pm 0.000$	$0.563 \pm 0.000$	284.71
*XGBoost*							
	CelebDF	$84.10 \pm 0.00$	$0.907 \pm 0.000$	$0.841 \pm 0.000$	$0.843 \pm 0.000$	$0.839 \pm 0.000$	0.43
	CelebDFC40	$58.13 \pm 0.00$	$0.615 \pm 0.000$	$0.542 \pm 0.000$	$0.598 \pm 0.000$	$0.495 \pm 0.000$	0.34
**Average**		**69.32**	**0.747**	**0.682**	−	−	−

Method: extratrees.

Values shown as Mean ± 95% Confidence Interval.

**Table 31:** Performance Results for extratrees+lasso Feature Selection (Celeb DF dataset).

**Classifier**	**Manipulation**	**Acc (%)**	**AUC**	**F1**	**Precision**	**Recall**	**Time (s)**
*AdaBoost*							
	CelebDF	$72.10 \pm 0.00$	$0.796 \pm 0.000$	$0.717 \pm 0.000$	$0.728 \pm 0.000$	$0.706 \pm 0.000$	10.07
	CelebDFC40	$67.10 \pm 0.00$	$0.722 \pm 0.000$	$0.656 \pm 0.000$	$0.687 \pm 0.000$	$0.628 \pm 0.000$	9.30
*DecisionTree*							
	CelebDF	$73.17 \pm 0.00$	$0.733 \pm 0.000$	$0.735 \pm 0.000$	$0.726 \pm 0.000$	$0.745 \pm 0.000$	2.77
	CelebDFC40	$48.40 \pm 0.00$	$0.482 \pm 0.000$	$0.487 \pm 0.000$	$0.484 \pm 0.000$	$0.489 \pm 0.000$	2.29
*ExtraTrees*							
	CelebDF	$83.27 \pm 0.00$	$0.907 \pm 0.000$	$0.832 \pm 0.000$	$0.836 \pm 0.000$	$0.827 \pm 0.000$	3.37
	CelebDFC40	$48.40 \pm 0.00$	$0.482 \pm 0.000$	$0.487 \pm 0.000$	$0.484 \pm 0.000$	$0.489 \pm 0.000$	3.67
*GradientBoosting*							
	CelebDF	$77.80 \pm 0.00$	$0.852 \pm 0.000$	$0.774 \pm 0.000$	$0.788 \pm 0.000$	$0.760 \pm 0.000$	54.54
	CelebDFC40	$68.07 \pm 0.00$	$0.749 \pm 0.000$	$0.662 \pm 0.000$	$0.703 \pm 0.000$	$0.625 \pm 0.000$	50.37
*KNN*							
	CelebDF	$83.67 \pm 0.00$	$0.904 \pm 0.000$	$0.833 \pm 0.000$	$0.851 \pm 0.000$	$0.817 \pm 0.000$	0.00
	CelebDFC40	$55.07 \pm 0.00$	$0.574 \pm 0.000$	$0.497 \pm 0.000$	$0.564 \pm 0.000$	$0.445 \pm 0.000$	0.00
*Logistic*							
	CelebDF	$75.10 \pm 0.00$	$0.823 \pm 0.000$	$0.745 \pm 0.000$	$0.763 \pm 0.000$	$0.729 \pm 0.000$	1.11
	CelebDFC40	$68.50 \pm 0.00$	$0.751 \pm 0.000$	$0.666 \pm 0.000$	$0.708 \pm 0.000$	$0.629 \pm 0.000$	0.79
*MLP*							
	CelebDF	$83.84 \pm 0.70$	$0.907 \pm 0.008$	$0.840 \pm 0.006$	$0.832 \pm 0.013$	$0.848 \pm 0.008$	47.10
	CelebDFC40	$58.72 \pm 0.53$	$0.625 \pm 0.011$	$0.548 \pm 0.011$	$0.606 \pm 0.010$	$0.500 \pm 0.023$	36.27
*RandomForest*							
	CelebDF	$82.63 \pm 0.00$	$0.902 \pm 0.000$	$0.828 \pm 0.000$	$0.821 \pm 0.000$	$0.834 \pm 0.000$	20.82
	CelebDFC40	$48.40 \pm 0.00$	$0.580 \pm 0.000$	$0.487 \pm 0.000$	$0.484 \pm 0.000$	$0.489 \pm 0.000$	20.11
*SVM-RBF*							
	CelebDF	$85.10 \pm 0.00$	$0.917 \pm 0.000$	$0.848 \pm 0.000$	$0.867 \pm 0.000$	$0.829 \pm 0.000$	173.71
	CelebDFC40	$66.93 \pm 0.00$	$0.731 \pm 0.000$	$0.627 \pm 0.000$	$0.719 \pm 0.000$	$0.557 \pm 0.000$	301.67
*XGBoost*							
	CelebDF	$83.60 \pm 0.00$	$0.909 \pm 0.000$	$0.836 \pm 0.000$	$0.838 \pm 0.000$	$0.833 \pm 0.000$	0.27
	CelebDFC40	$57.20 \pm 0.00$	$0.622 \pm 0.000$	$0.537 \pm 0.000$	$0.585 \pm 0.000$	$0.497 \pm 0.000$	0.28
**Average**		**69.35**	**0.748**	**0.682**	−	−	−

Method: extratrees+lasso.

Values shown as Mean ± 95% Confidence Interval.

**Table 32:** Performance Results for kbest Feature Selection (Celeb DF dataset).

**Classifier**	**Manipulation**	**Acc (%)**	**AUC**	**F1**	**Precision**	**Recall**	**Time (s)**
*AdaBoost*							
	CelebDF	$72.04 \pm 0.19$	$0.783 \pm 0.003$	$0.717 \pm 0.003$	$0.726 \pm 0.001$	$0.709 \pm 0.005$	10.15
	CelebDFC40	$66.69 \pm 0.19$	$0.712 \pm 0.004$	$0.636 \pm 0.008$	$0.700 \pm 0.014$	$0.583 \pm 0.023$	10.33
*DecisionTree*							
	CelebDF	$71.50 \pm 2.15$	$0.715 \pm 0.022$	$0.721 \pm 0.025$	$0.706 \pm 0.015$	$0.737 \pm 0.034$	2.65
	CelebDFC40	$48.40 \pm 0.00$	$0.482 \pm 0.000$	$0.487 \pm 0.000$	$0.484 \pm 0.000$	$0.489 \pm 0.000$	2.61
*ExtraTrees*							
	CelebDF	$81.31 \pm 0.62$	$0.893 \pm 0.004$	$0.813 \pm 0.006$	$0.814 \pm 0.007$	$0.812 \pm 0.005$	3.67
	CelebDFC40	$48.40 \pm 0.00$	$0.482 \pm 0.000$	$0.487 \pm 0.000$	$0.484 \pm 0.000$	$0.489 \pm 0.000$	4.06
*GradientBoosting*							
	CelebDF	$75.58 \pm 0.62$	$0.826 \pm 0.002$	$0.751 \pm 0.009$	$0.766 \pm 0.000$	$0.737 \pm 0.017$	55.04
	CelebDFC40	$67.99 \pm 0.46$	$0.744 \pm 0.005$	$0.655 \pm 0.006$	$0.710 \pm 0.004$	$0.608 \pm 0.007$	56.30
*KNN*							
	CelebDF	$82.86 \pm 1.67$	$0.894 \pm 0.002$	$0.824 \pm 0.017$	$0.845 \pm 0.019$	$0.805 \pm 0.015$	0.00
	CelebDFC40	$54.31 \pm 1.27$	$0.564 \pm 0.009$	$0.475 \pm 0.023$	$0.558 \pm 0.016$	$0.413 \pm 0.029$	0.00
*Logistic*							
	CelebDF	$74.30 \pm 1.29$	$0.808 \pm 0.007$	$0.739 \pm 0.015$	$0.750 \pm 0.009$	$0.728 \pm 0.022$	0.86
	CelebDFC40	$66.37 \pm 0.82$	$0.723 \pm 0.002$	$0.638 \pm 0.010$	$0.691 \pm 0.008$	$0.593 \pm 0.011$	0.90
*MLP*							
	CelebDF	$81.63 \pm 1.19$	$0.887 \pm 0.011$	$0.818 \pm 0.014$	$0.812 \pm 0.007$	$0.823 \pm 0.026$	42.97
	CelebDFC40	$56.69 \pm 3.73$	$0.593 \pm 0.039$	$0.546 \pm 0.036$	$0.574 \pm 0.042$	$0.521 \pm 0.030$	39.25
*RandomForest*							
	CelebDF	$80.89 \pm 0.48$	$0.884 \pm 0.004$	$0.810 \pm 0.002$	$0.806 \pm 0.012$	$0.814 \pm 0.008$	23.14
	CelebDFC40	$48.36 \pm 0.10$	$0.577 \pm 0.001$	$0.487 \pm 0.000$	$0.484 \pm 0.001$	$0.489 \pm 0.000$	24.07
*SVM-RBF*							
	CelebDF	$82.32 \pm 0.53$	$0.892 \pm 0.003$	$0.819 \pm 0.008$	$0.841 \pm 0.003$	$0.797 \pm 0.017$	184.78
	CelebDFC40	$66.13 \pm 0.58$	$0.733 \pm 0.001$	$0.604 \pm 0.009$	$0.728 \pm 0.007$	$0.516 \pm 0.013$	302.01
*XGBoost*							
	CelebDF	$81.83 \pm 0.29$	$0.894 \pm 0.004$	$0.819 \pm 0.001$	$0.817 \pm 0.009$	$0.820 \pm 0.008$	0.36
	CelebDFC40	$58.00 \pm 1.66$	$0.615 \pm 0.011$	$0.538 \pm 0.027$	$0.598 \pm 0.018$	$0.490 \pm 0.037$	0.42
**Average**		**68.28**	**0.735**	**0.669**	−	−	−

Method: kbest *Values shown as Mean* ± *95% Confidence Interval*.

**Table 33:** Performance Results for kbest+lasso Feature Selection (Celeb DF dataset).

**Classifier**	**Manipulation**	**Acc (%)**	**AUC**	**F1**	**Precision**	**Recall**	**Time (s)**
*AdaBoost*							
	CelebDF	$71.56 \pm 1.24$	$0.780 \pm 0.010$	$0.712 \pm 0.015$	$0.722 \pm 0.009$	$0.702 \pm 0.020$	9.74
	CelebDFC40	$66.60 \pm 0.57$	$0.712 \pm 0.004$	$0.635 \pm 0.003$	$0.700 \pm 0.018$	$0.582 \pm 0.017$	9.38
*DecisionTree*							
	CelebDF	$71.02 \pm 1.39$	$0.711 \pm 0.013$	$0.718 \pm 0.008$	$0.700 \pm 0.020$	$0.736 \pm 0.005$	2.54
	CelebDFC40	$48.40 \pm 0.00$	$0.482 \pm 0.000$	$0.487 \pm 0.000$	$0.484 \pm 0.000$	$0.489 \pm 0.000$	2.35
*ExtraTrees*							
	CelebDF	$81.77 \pm 0.43$	$0.892 \pm 0.006$	$0.817 \pm 0.003$	$0.820 \pm 0.010$	$0.814 \pm 0.005$	3.39
	CelebDFC40	$48.40 \pm 0.00$	$0.482 \pm 0.000$	$0.487 \pm 0.000$	$0.484 \pm 0.000$	$0.489 \pm 0.000$	3.75
*GradientBoosting*							
	CelebDF	$75.41 \pm 0.91$	$0.827 \pm 0.002$	$0.750 \pm 0.011$	$0.763 \pm 0.006$	$0.737 \pm 0.015$	52.80
	CelebDFC40	$68.00 \pm 0.44$	$0.744 \pm 0.006$	$0.655 \pm 0.003$	$0.710 \pm 0.007$	$0.608 \pm 0.003$	51.02
*KNN*							
	CelebDF	$83.20 \pm 0.00$	$0.895 \pm 0.006$	$0.827 \pm 0.001$	$0.854 \pm 0.005$	$0.802 \pm 0.007$	0.00
	CelebDFC40	$54.76 \pm 2.63$	$0.565 \pm 0.021$	$0.486 \pm 0.032$	$0.562 \pm 0.034$	$0.428 \pm 0.031$	0.00
*Logistic*							
	CelebDF	$74.32 \pm 1.24$	$0.808 \pm 0.007$	$0.739 \pm 0.015$	$0.750 \pm 0.008$	$0.729 \pm 0.021$	0.78
	CelebDFC40	$66.39 \pm 0.42$	$0.723 \pm 0.002$	$0.638 \pm 0.005$	$0.691 \pm 0.005$	$0.592 \pm 0.006$	0.90
*MLP*							
	CelebDF	$80.28 \pm 1.76$	$0.877 \pm 0.009$	$0.806 \pm 0.018$	$0.793 \pm 0.017$	$0.820 \pm 0.024$	46.77
	CelebDFC40	$58.80 \pm 2.23$	$0.618 \pm 0.018$	$0.549 \pm 0.037$	$0.607 \pm 0.030$	$0.501 \pm 0.060$	35.11
*RandomForest*							
	CelebDF	$80.59 \pm 0.33$	$0.882 \pm 0.003$	$0.807 \pm 0.002$	$0.804 \pm 0.006$	$0.810 \pm 0.002$	19.74
	CelebDFC40	$48.40 \pm 0.00$	$0.577 \pm 0.005$	$0.487 \pm 0.000$	$0.484 \pm 0.000$	$0.489 \pm 0.000$	21.06
*SVM-RBF*							
	CelebDF	$81.98 \pm 1.77$	$0.889 \pm 0.006$	$0.816 \pm 0.018$	$0.835 \pm 0.020$	$0.797 \pm 0.015$	184.24
	CelebDFC40	$65.96 \pm 0.77$	$0.733 \pm 0.004$	$0.600 \pm 0.009$	$0.728 \pm 0.011$	$0.510 \pm 0.008$	300.22
*XGBoost*							
	CelebDF	$81.32 \pm 0.67$	$0.888 \pm 0.003$	$0.814 \pm 0.008$	$0.812 \pm 0.003$	$0.816 \pm 0.013$	0.31
	CelebDFC40	$57.77 \pm 1.74$	$0.617 \pm 0.005$	$0.532 \pm 0.012$	$0.597 \pm 0.025$	$0.480 \pm 0.008$	0.30
**Average**		**68.25**	**0.735**	**0.668**	−	−	−

Method: kbest+lasso.

Values shown as Mean ± 95% Confidence Interval.

**Table 34:** Performance Results for rfe Feature Selection (Celeb DF dataset).

**Classifier**	**Manipulation**	**Acc (%)**	**AUC**	**F1**	**Precision**	**Recall**	**Time (s)**
*AdaBoost*							
	CelebDF	$71.27 \pm 0.00$	$0.788 \pm 0.000$	$0.713 \pm 0.000$	$0.712 \pm 0.000$	$0.715 \pm 0.000$	7.25
	CelebDFC40	$62.00 \pm 0.00$	$0.674 \pm 0.000$	$0.609 \pm 0.000$	$0.627 \pm 0.000$	$0.592 \pm 0.000$	6.33
*DecisionTree*							
	CelebDF	$66.57 \pm 0.00$	$0.666 \pm 0.000$	$0.674 \pm 0.000$	$0.657 \pm 0.000$	$0.693 \pm 0.000$	1.74
	CelebDFC40	$48.40 \pm 0.00$	$0.482 \pm 0.000$	$0.487 \pm 0.000$	$0.484 \pm 0.000$	$0.489 \pm 0.000$	1.57
*ExtraTrees*							
	CelebDF	$78.10 \pm 0.00$	$0.872 \pm 0.000$	$0.781 \pm 0.000$	$0.782 \pm 0.000$	$0.779 \pm 0.000$	3.85
	CelebDFC40	$48.40 \pm 0.00$	$0.482 \pm 0.000$	$0.487 \pm 0.000$	$0.484 \pm 0.000$	$0.489 \pm 0.000$	4.19
*GradientBoosting*							
	CelebDF	$74.20 \pm 0.00$	$0.820 \pm 0.000$	$0.738 \pm 0.000$	$0.750 \pm 0.000$	$0.726 \pm 0.000$	38.78
	CelebDFC40	$64.30 \pm 0.00$	$0.697 \pm 0.000$	$0.623 \pm 0.000$	$0.660 \pm 0.000$	$0.591 \pm 0.000$	34.09
*KNN*							
	CelebDF	$75.30 \pm 0.00$	$0.822 \pm 0.000$	$0.744 \pm 0.000$	$0.772 \pm 0.000$	$0.719 \pm 0.000$	0.00
	CelebDFC40	$53.33 \pm 0.00$	$0.562 \pm 0.000$	$0.492 \pm 0.000$	$0.540 \pm 0.000$	$0.451 \pm 0.000$	0.00
*Logistic*							
	CelebDF	$71.30 \pm 0.00$	$0.790 \pm 0.000$	$0.712 \pm 0.000$	$0.715 \pm 0.000$	$0.709 \pm 0.000$	0.48
	CelebDFC40	$64.73 \pm 0.00$	$0.697 \pm 0.000$	$0.631 \pm 0.000$	$0.662 \pm 0.000$	$0.603 \pm 0.000$	0.27
*MLP*							
	CelebDF	$75.09 \pm 1.28$	$0.825 \pm 0.005$	$0.755 \pm 0.016$	$0.742 \pm 0.024$	$0.769 \pm 0.042$	37.74
	CelebDFC40	$55.17 \pm 0.80$	$0.577 \pm 0.015$	$0.540 \pm 0.013$	$0.554 \pm 0.007$	$0.527 \pm 0.019$	36.13
*RandomForest*							
	CelebDF	$78.27 \pm 0.00$	$0.868 \pm 0.000$	$0.784 \pm 0.000$	$0.780 \pm 0.000$	$0.787 \pm 0.000$	15.88
	CelebDFC40	$48.40 \pm 0.00$	$0.557 \pm 0.000$	$0.487 \pm 0.000$	$0.484 \pm 0.000$	$0.489 \pm 0.000$	14.90
*SVM-RBF*							
	CelebDF	$76.23 \pm 0.00$	$0.840 \pm 0.000$	$0.760 \pm 0.000$	$0.768 \pm 0.000$	$0.751 \pm 0.000$	243.45
	CelebDFC40	$62.73 \pm 0.00$	$0.674 \pm 0.000$	$0.597 \pm 0.000$	$0.650 \pm 0.000$	$0.553 \pm 0.000$	330.42
*XGBoost*							
	CelebDF	$77.70 \pm 0.00$	$0.856 \pm 0.000$	$0.778 \pm 0.000$	$0.775 \pm 0.000$	$0.781 \pm 0.000$	0.43
	CelebDFC40	$57.63 \pm 0.00$	$0.609 \pm 0.000$	$0.553 \pm 0.000$	$0.585 \pm 0.000$	$0.525 \pm 0.000$	0.47
**Average**		**65.46**	**0.708**	**0.647**	−	−	−

Method: rfe.

Values shown as Mean ± 95% Confidence Interval.

**Table 35:** Performance Results for rfe+lasso Feature Selection (Celeb DF dataset).

**Classifier**	**Manipulation**	**Acc (%)**	**AUC**	**F1**	**Precision**	**Recall**	**Time (s)**
*AdaBoost*							
	CelebDF	$71.27 \pm 0.00$	$0.788 \pm 0.000$	$0.713 \pm 0.000$	$0.712 \pm 0.000$	$0.715 \pm 0.000$	7.18
	CelebDFC40	$62.00 \pm 0.00$	$0.674 \pm 0.000$	$0.609 \pm 0.000$	$0.627 \pm 0.000$	$0.592 \pm 0.000$	6.31
*DecisionTree*							
	CelebDF	$66.57 \pm 0.00$	$0.666 \pm 0.000$	$0.674 \pm 0.000$	$0.657 \pm 0.000$	$0.693 \pm 0.000$	1.74
	CelebDFC40	$48.40 \pm 0.00$	$0.482 \pm 0.000$	$0.487 \pm 0.000$	$0.484 \pm 0.000$	$0.489 \pm 0.000$	1.57
*ExtraTrees*							
	CelebDF	$78.10 \pm 0.00$	$0.872 \pm 0.000$	$0.781 \pm 0.000$	$0.782 \pm 0.000$	$0.779 \pm 0.000$	3.83
	CelebDFC40	$48.40 \pm 0.00$	$0.482 \pm 0.000$	$0.487 \pm 0.000$	$0.484 \pm 0.000$	$0.489 \pm 0.000$	4.21
*GradientBoosting*							
	CelebDF	$74.20 \pm 0.00$	$0.820 \pm 0.000$	$0.738 \pm 0.000$	$0.750 \pm 0.000$	$0.726 \pm 0.000$	38.70
	CelebDFC40	$64.30 \pm 0.00$	$0.697 \pm 0.000$	$0.623 \pm 0.000$	$0.660 \pm 0.000$	$0.591 \pm 0.000$	34.06
*KNN*							
	CelebDF	$75.30 \pm 0.00$	$0.822 \pm 0.000$	$0.744 \pm 0.000$	$0.772 \pm 0.000$	$0.719 \pm 0.000$	0.00
	CelebDFC40	$53.33 \pm 0.00$	$0.562 \pm 0.000$	$0.492 \pm 0.000$	$0.540 \pm 0.000$	$0.451 \pm 0.000$	0.00
*Logistic*							
	CelebDF	$71.30 \pm 0.00$	$0.790 \pm 0.000$	$0.712 \pm 0.000$	$0.715 \pm 0.000$	$0.709 \pm 0.000$	0.60
	CelebDFC40	$64.73 \pm 0.00$	$0.697 \pm 0.000$	$0.631 \pm 0.000$	$0.662 \pm 0.000$	$0.603 \pm 0.000$	0.46
*MLP*							
	CelebDF	$75.31 \pm 0.97$	$0.829 \pm 0.005$	$0.758 \pm 0.009$	$0.743 \pm 0.028$	$0.775 \pm 0.042$	35.18
	CelebDFC40	$55.36 \pm 1.01$	$0.578 \pm 0.021$	$0.538 \pm 0.029$	$0.557 \pm 0.010$	$0.521 \pm 0.054$	41.15
*RandomForest*							
	CelebDF	$78.27 \pm 0.00$	$0.868 \pm 0.000$	$0.784 \pm 0.000$	$0.780 \pm 0.000$	$0.787 \pm 0.000$	15.82
	CelebDFC40	$48.40 \pm 0.00$	$0.557 \pm 0.000$	$0.487 \pm 0.000$	$0.484 \pm 0.000$	$0.489 \pm 0.000$	14.98
*SVM-RBF*							
	CelebDF	$76.23 \pm 0.00$	$0.840 \pm 0.000$	$0.760 \pm 0.000$	$0.768 \pm 0.000$	$0.751 \pm 0.000$	241.02
	CelebDFC40	$62.73 \pm 0.00$	$0.674 \pm 0.000$	$0.597 \pm 0.000$	$0.650 \pm 0.000$	$0.553 \pm 0.000$	323.60
*XGBoost*							
	CelebDF	$77.70 \pm 0.00$	$0.856 \pm 0.000$	$0.778 \pm 0.000$	$0.775 \pm 0.000$	$0.781 \pm 0.000$	0.36
	CelebDFC40	$57.63 \pm 0.00$	$0.609 \pm 0.000$	$0.553 \pm 0.000$	$0.585 \pm 0.000$	$0.525 \pm 0.000$	0.36
**Average**		**65.48**	**0.708**	**0.647**	−	−	−

Method: rfe+lasso.

Values shown as Mean ± 95% Confidence Interval.

## Discussion

Our results show that increased compression decreases the classification accuracy for both FF++ (C23, C40) and Celeb-DF v2 (C0, C40), indicating a performance efficiency trade-off. The SVMs, random forest, and XGBoost are extremely resilient to compression noise, and the KNN is fair with moderate compression but decreases significantly with heavy compression.

For Celeb-DF v2, the GA-based models perform competitively even with C40 compression, whereas the GA+SVM achieves 78.74 AUC and 71.47% accuracy, which highlights the ability of the GA to preserve semantically informative features. The GA outperforms the GA+LASSO hybrid model, which can overprune meaningful patterns under compression, underscoring the role of feature selection flexibility.

Cross-manipulation testing has difficulty in generalization across compression types of forgeries because the interaction between artifacts and compression noise reduces robustness. Compared with benchmarks, deep learning models (e.g., F3-Net, MfDfD and ViViT) are more accurate with respect to benchmarks but less interpretable and more computationally demanding.

Our strategy optimizes accuracy, interpretability, and efficiency. Ensemble methods combined with GA-selected features provide competitive results on difficult datasets and are still feasible for limited-resource or real-time applications. Additionally, compression-conscious feature selection is a direction for future research to further bridge the gap toward end-to-end deep models.

## Limitations

This study has several limitations that suggest directions for future work. Firstly, feature extraction relies on a ResNet-50 model pretrained on ImageNet, which may not optimally capture manipulation-specific cues inherent to deepfake videos. Secondly, the pipeline focuses on spatial (frame-level) analysis and lacks explicit temporal modeling, which is crucial for detecting inconsistencies in dynamic video content. Thirdly, while the method is evaluated under multiple compression levels, it is not explicitly designed to be compression-aware, raising uncertainty about its robustness with unseen compression schemes or variable bit rates. Finally, although genetic algorithms are used for feature selection, their computational complexity may limit scalability for massive datasets or real-time applications. Future work could also explore adaptive regularization (e.g., Elastic Net, Group LASSO) and alternative models to enhance feature representation.

## Conclusion

This paper proposes a hybrid deepfake detection framework that combines ResNet50-based deep feature extraction with two-stage feature selection via GA and LASSO. The method demonstrates robustness to manipulation variability and compression artifacts. Evaluated on FF++ and Celeb-DF v2, it achieves high within-manipulation accuracy (AUC up to 99.48 under C23 FF++) and strong cross-manipulation generalizability, particularly on FF++.

Unlike purely state-of-the-art end-to-end models, our study emphasizes practical alternatives that maintain competitive accuracy while offering lighter computational requirements. The framework was rigorously validated using multiple random seeds and ten classifiers, demonstrating stable improvements in both accuracy and AUC. GA+LASSO provides a favorable trade-off between feature sparsity and runtime, especially in compressed scenarios.

Future work will explore advanced metaheuristic selection strategies, adaptive multi-modal features, and dynamic selection mechanisms to further enhance robustness against evolving synthetic media attacks while retaining efficiency for resource-constrained applications.

## Data Availability

The datasets analysed in this study are publicly available. The Celeb-DF dataset can be accessed from the official repository at https://github.com/yuezunli/celeb-deepfakeforensics, and the FaceForensics++ dataset is available at https://github.com/ondyari/FaceForensics. No new datasets were generated during the current study.
